# Molecular-oxygen-promoted Cu-catalyzed oxidative direct amidation of nonactivated carboxylic acids with azoles

**DOI:** 10.3762/bjoc.11.233

**Published:** 2015-11-11

**Authors:** Wen Ding, Shaoyu Mai, Qiuling Song

**Affiliations:** 1 Institute of Next Generation Matter Transformation, College of Chemical Engineering at Huaqiao Univeristy, P. R. China; 2 College of Materials Science at Huaqiao University, 668 Jimei Blvd, Xiamen, Fujian, 361021, P. R. China; 3 Beijing National Laboratory for Molecular Sciences, Beijing, 100190, P. R. China

**Keywords:** amidation, azoles, Cu-catalyzed, molecular oxygen, transamidation

## Abstract

A copper-catalyzed oxidative direct formation of amides from nonactivated carboxylic acids and azoles with dioxygen as an activating reagent is reported. The azole amides were produced in good to excellent yields with a broad substrate scope. The mechanistic studies reveal that oxygen plays an essential role in the success of the amidation reactions with copper peroxycarboxylate as the key intermediate. Transamidation occurs smoothly between azole amide and a variety of amines.

## Introduction

**Scheme 1:**
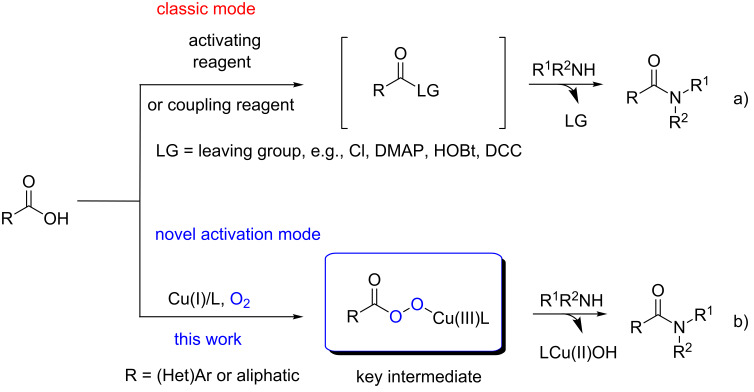
Traditional activating mode and oxidative activation mode of free carboxylic acids in amide formation.

Amides are prevalent scaffolds in numerous compounds ranging from biologically active natural products to pharmaceuticals [1]. As a result, numerous methods have been developed for the formation of amides [2–7]. Despite such advances [8–21], the acylation of amines with free carboxylic acids is still the most common method employed due to the availability of the starting materials and its relatively clean process feature, whereby water is the only byproduct in the transformation [22]. However, preactivation of the free carboxylic acids is always required, and stoichiometric activating reagents or coupling reagents are mandatory in the classic methods of amide formation (Scheme 1a) [23].

In some cases, corrosive byproducts (HCl) are unavoidable and the activating reagent is difficult to remove from the system. Given the growing demand for environmentally benign synthesis [24], it is highly desirable to bring forth a green, sustainable and simple new protocol for the activation of carboxylic acids. Molecular oxygen is an ideal oxidant owing to its negligible cost, availability and sustainability [25–26]. Currently, copper-catalyzed, aerobic, oxidative transformations with dioxygen as the sole oxidant have emerged as a useful and powerful strategy to construct carbon–carbon and carbon–heteroatom bonds [27–29].

It is known that Cu(II) superoxide species are usually reported to be formed in the system, and very recently McDonald and coworkers reported an unusual nucleophilic reactivity of a special Cu(II) superoxide complex, acting as a deformylating reagent in spite of the prevailing electrophilic property common for such a metal-bound superoxide intermediate [30]. Therefore, we envision that the newly discovered nucleophilic reactivity of Cu(II) superoxide species might lead to a novel, free carboxylic acid activation mode for amide formation: a Cu(II) superoxide species could attack the carbonyl group in an acid to afford a copper peroxycarboxylate, which might then serve as the key intermediate to make the subsequent amide formation feasible (Scheme 1b). In our work, azoles have been chosen as the amines due to their special bioactivity [31]. To the best of our knowledge, Cu salt has not yet been used for catalyzed, oxidative direct amide formation. We report the first amidation reaction from carboxylic acids with peroxycarboxylate as the key intermediate, which represents a novel activation mode with molecular oxygen as the activating reagent. Most remarkably, in sharp contrast to previous reports (which used complex N-containing ligands to form copper superoxide), inexpensive and readily available pyridine was employed as both the ligand and base in our case.

## Results and Discussion

Our initial exploration commenced with benzoic acid (**1**) and benzimidazole (**2**) as the model substrates to investigate the copper-catalyzed oxidative direct amidation reaction (Table 1).

**Table 1:** Optimization of the reaction conditions.^a^

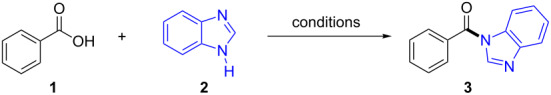						

entry	catalyst (mol %)	atmosphere	ligand (equiv)	temperature	solvent	yield (%)^b^

1	CuCl (10)	O_2_	pyridine (3)	130 °C	*p*-xylene	33
2	CuI (10)	O_2_	pyridine (3)	130 °C	*p*-xylene	79
3	Cu(OAc)_2_ (10)	O_2_	pyridine (3)	130 °C	*p*-xylene	0
4	CuBr (10)	O_2_	pyridine (3)	130 °C	*p*-xylene	97 (85)^c^
5	CuBr_2_ (10)	O_2_	pyridine (3)	130 °C	*p*-xylene	33
6	CuBr (10)	O_2_	pyridine (3)	130 °C	*o*-xylene	48
7	CuBr (10)	O_2_	pyridine (3)	130 °C	DMSO	no product
8	CuBr (10)	O_2_	pyridine (3)	130 °C	DMF	no product
9	CuBr (10)	O_2_	pyridine (3)	130 °C	toluene	4
10	CuBr (10)	O_2_	1, 10-Phen (3)	130 °C	*p*-xylene	no product
11	CuBr (10)	O_2_	2, 2'-bipy (3)	130 °C	*p*-xylene	no product
12	CuBr (10)	O_2_	2-aminopyridine (3)	130 °C	*p*-xylene	no product
13	CuBr (10)	O_2_	Et_3_N (3)	130 °C	*p*-xylene	no product
14	CuBr (10)	O_2_	pyridine (3)	120 °C	*p*-xylene	31
15	CuBr (10)	O_2_	pyridine (0.5)	130 °C	*p*-xylene	39
16	CuBr (10)	O_2_	pyridine (2)	130 °C	*p*-xylene	77
17^d^	CuBr (10)	O_2_	pyridine (3)	130 °C	*p*-xylene	7
18	CuBr (10)	N_2_	pyridine (3)	130 °C	*p*-xylene	no product
19	CuBr (10)	air	pyridine (3)	130 °C	*p*-xylene	53
20	–	O_2_	pyridine (3)	130 °C	*p*-xylene	no product
21	CuBr (10)	O_2_	–	130 °C	*p*-xylene	46

^a^Reaction conditions: all reactions were performed with a mixture of **1** (0.25 mmol), **2** (0.5 mmol), Cu salt (10 mol %), ligand, solvent (1 mL), 16 h, temp., corresponding atmosphere. ^b^GC yield. ^c^Isolated yield. ^d^**1** (0.25 mmol), **2** (0.30 mmol).

The copper salts demonstrated good activity for this novel transformation (Table 1, entries 1–5) and 85% of the desired product was obtained using 10 mol % of CuBr with pyridine (3 equiv) at 130 °C in *p*-xylene (1 mL) under O_2_ in a sealed tube (Table 1, entry 4). Notably, CuBr_2_ only gave 33% of the desired product under similar conditions (Table 1, entry 5), inferring that molecular oxygen was not just acting as an oxidant to convert Cu(I) into Cu(II), but that it might be involved in the reaction. Further screening of solvents and ligands revealed that *p*-xylene and pyridine are the optimal choices. The reaction cannot go to completion and part of the carboxylic acid remains when the amount of benzimidazole is not twice that of the carboxylic acid. The temperature affected this reaction dramatically and when the temperature was decreased to 120 °C, the yield of the desired product **3** was reduced to 31% of the corresponding GC yield (Table 1, entry 14). Copper salt, pyridine and dioxygen were all found to be pivotal to this transformation (Table 1, entries 19–21): without CuBr, no desired product was detected; in the absence of pyridine, the yield of the desired product was reduced to 46% with some unknown compounds generated; when the reactions were conducted under N_2_ atmosphere or air, the desired products were either not detected or reduced to 53%.

**Scheme 2:**
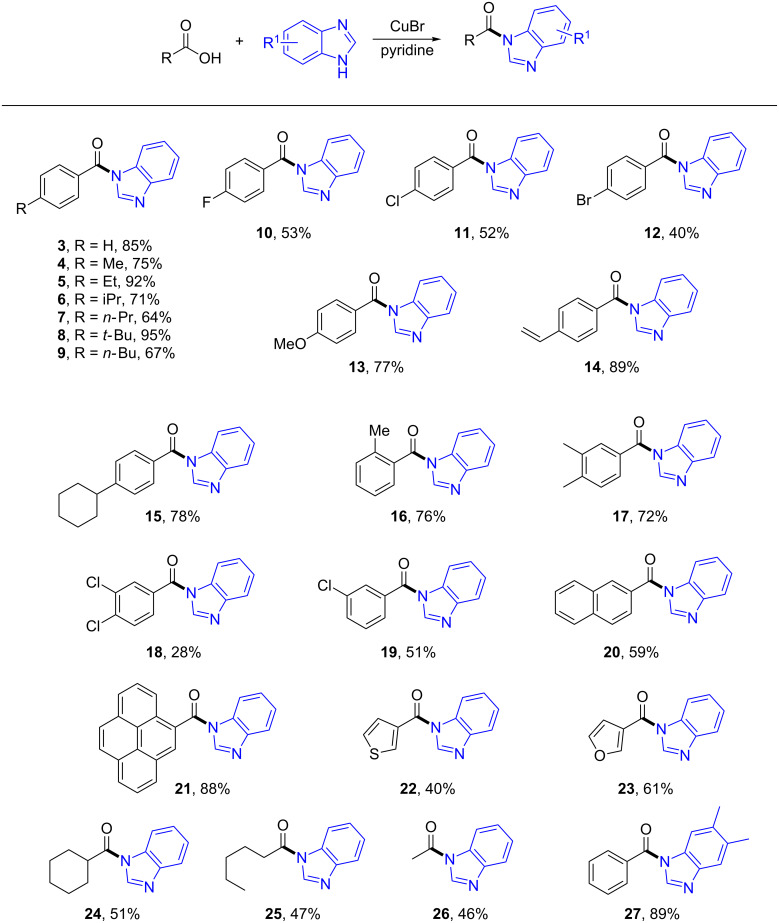
Substrate scope for catalytic, direct amide formation from carboxylic acids and azoles. Reaction conditions: carboxylic acid (0.25 mmol), azole derivative (0.5 mmol), CuBr (10 mol %), pyridine (0.75 mmol), *p*-xylene (1 mL), 130 °C, 20 h, under O_2_ atmosphere in a sealed tube. Isolated yield based on carboxylic acids.

With the optimized conditions in hand, we studied the scope and limitations of the Cu-catalyzed oxidative direct amidation reaction. First, we surveyed different carboxylic acids. To our delight, both aromatic and aliphatic carboxylic acids were competent candidates in this transformation (Scheme 2).

For aromatic carboxylic acids, various substituents on the aromatic ring were tolerable under the standard conditions, which suggests that the reaction has a broad substrate scope. Electron-donating (**4**–**9**, **13**, and **15**–**17**) and electron-withdrawing groups (**10**–**12**, **14**, **18**, and **19**) are also compatible in this reaction. Meanwhile, other aromatic acids such as 2-naphthylcarboxylic acid and pyrene-4-carboxylic acid also worked well under the reaction conditions, providing the desired products **20** and **21** in 59% and 88% yield, respectively. Additionally, heterocyclic acids, such as thiophene- and furan-3-carboxylic acid were well tolerated, providing the amides **22** and **23** in acceptable yields. Gratifyingly, aliphatic carboxylic acids, such as cyclohexanecarboxylic acid, hexanoic acid and acetic acid were all proven to be good substrates in this transformation and the desired products **24**, **25** and **26** were obtained in reasonable yields. Moreover, bis-substituted benzimidazole also worked very well under the standard conditions to give the desired product **27** in 89% yield.

**Scheme 3:**
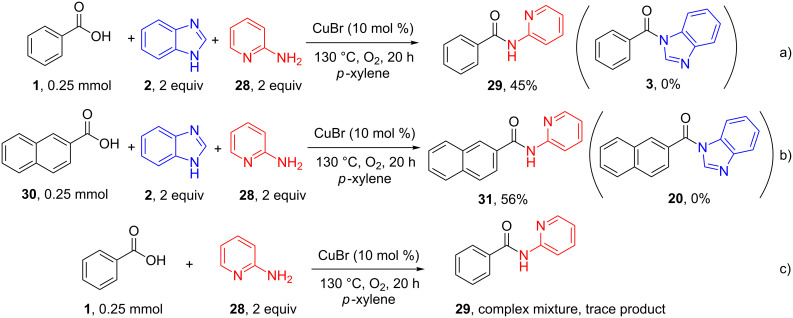
Further investigation into the scope of amine.

The scope of amine was further investigated and it seems that only benzimidazole and its derivatives were good candidates in the above direct amide formation (see Scheme S1 in Supporting Information File 1 for details). However, remarkably, when 2-aminopyridine (**28**) and benzimidazole (**2**) were mixed with benzoic acid under the standard conditions, compound **29** was obtained in 45% yield instead of the predicted amide **3** (Scheme 3a). Compound **3** was not detected in the reaction. Similarly, when 2-naphthoic acid (**30**) was used instead of benzoic acid (**1**), compound **31** was afforded in 56% yield without formation of compound **20** (Scheme 3b). However, when a control experiment was conducted without benzimidazole (**2**) (Scheme 3c), compound **29** was not detected and the reaction resulted in a complex mixture of undesired compounds.

**Scheme 4:**
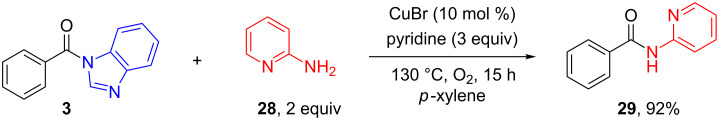
Possible transamidation process.

In order to determine the possibility of transamidation between compound **3** and 2-aminopyridine (**28**), azole amide **3** was mixed with 2-aminopyridine (**28**) under the standard conditions. Surprisingly, the azole amide **3** was transformed into **29** in 92% yield, which clearly demonstrated that transamidation occurred in this process (Scheme 4).

**Scheme 5:**
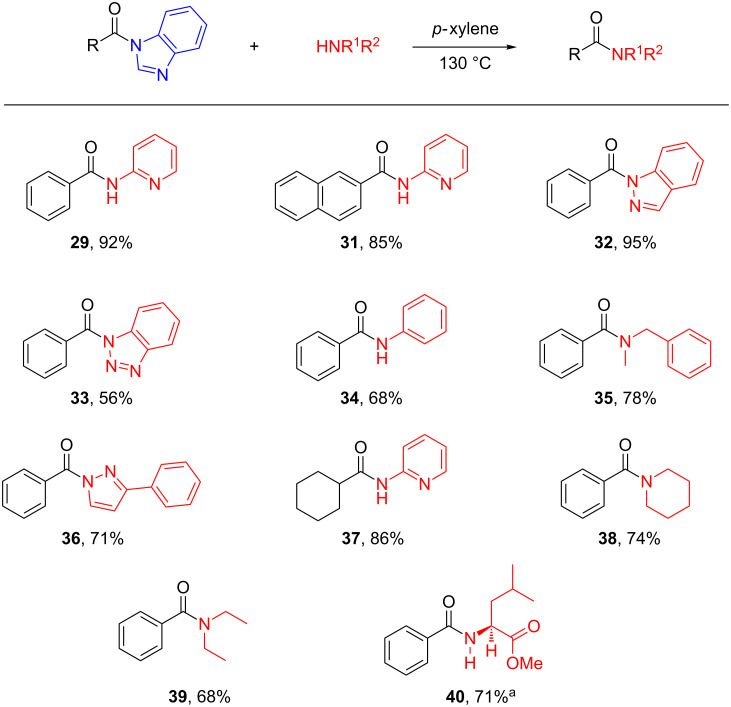
Scope of the amine transamidation from benzimidazole amides. Reaction conditions: benzimidazole amide (0.25 mmol), amine (0.375 mmol), *p*-xylene (1 mL), 130 °C, sealed tube. Isolated yields. ^a^Methyl L-leucinate hydrochloride as the ammonia source and Et_3_N (1.5 equiv) were added, the mixture was stirred at room temperature for 3 min, then the mixture was heated to 130 °C.

Further exploration into a variety of amines suggested that transamidation could be efficiently accomplished in the absence of the copper catalyst, pyridine and the versatile amines, including aromatic (**29**, **31**–**34**, **36** and **37**) and aliphatic (**35**, **38**–**40**), primary (**29**, **31**, **34**, **37** and **40**) and secondary (**32**, **33**, **35**, **36**, **38** and **39**). Even the amino group as a natural product (methyl L-leucinate hydrochloride, **40**) was compatible under the conditions, affording corresponding amides in good to excellent yield (Scheme 5), suggesting the potential utility in complex molecule manipulation. In addition, for the carboxylic acid part, both aromatic and aliphatic (**37**) compounds were competent as well.

**Scheme 6:**
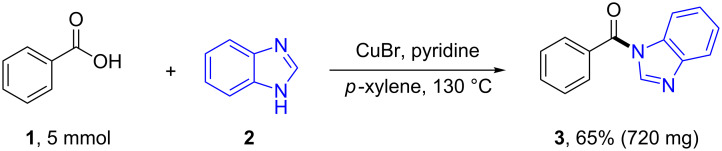
Preparative scale of the reaction.

The amidation reaction could be easily scaled up without significant decrease in the yield (Scheme 6).

**Scheme 7:**
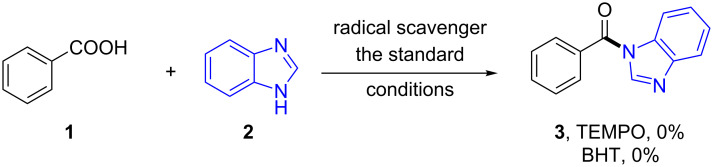
Radical scavenger reaction.

Radical trapping experiments were performed in order to gain further insight into the mechanism of the catalytic direct amide formation from nonactivated carboxylic acids. The reactions were totally inhibited by radical scavengers 2,2,6,6-tetramethyl-1-piperidinyloxy (TEMPO) and BHT (2,6-di-*tert*-butyl-4-methylphenol), suggesting that a radical pathway might be involved in these transformations (Scheme 7).

**Scheme 8:**
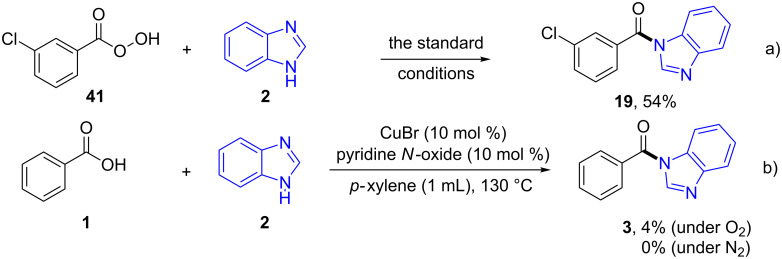
Control reactions.

A careful look at the literature reveals that Cu(II) superoxide species were reported to be formed under our standard conditions and might act as a nucleophile to attack carbonyl groups in certain cases [30], although it was always reported as an electrophile [32]. Thus, a new intermediate, peroxycarboxylate, will be formed in our case. In order to figure out whether peroxycarboxylate acts as our key intermediate, the readily available *m-*CPBA (**41**) was used as substrate under the standard conditions and the corresponding product **19** was obtained in 54% yield (Scheme 8a).

Compared to the isolated yield from *m*-chlorophenylacetic acid (Scheme 2, **19**, 51%), we have reason to believe that the above proposed mechanism is plausible. In addition, pyridine *N*-oxide was applied to the benzoic acid standard conditions in the presence/absence of dioxygen. However, only a trace amount of the desired product **3** was ever detected, thus it was ruled out as a possible pathway (Scheme 8b).

**Scheme 9:**
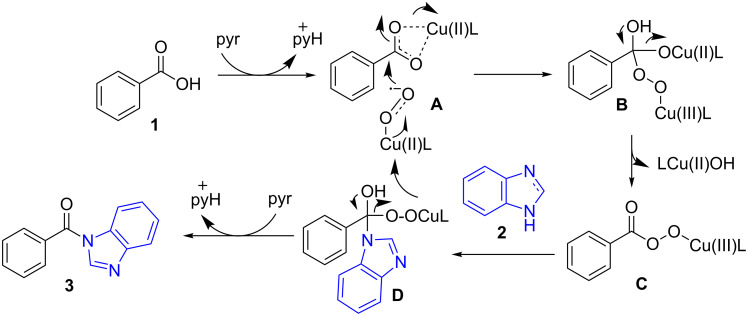
Proposed mechanism.

Based on the above control experiments, we postulated a tentative mechanism (Scheme 9). The copper catalyst was postulated to play dual roles in the initial activation of benzoic acid: (1) it acts as a Lewis acid to activate benzoic acid (**1**), making it vulnerable to nucleophilic attack and (2) it forms Cu(II) superoxide species [30,32–34] to attack activated benzoic acid **A** to afford intermediate **B**. Under the standard conditions, intermediate **B** was switched to intermediate **C**, once again, with the nucleophilic attack from benzimidazole (**2**) and eventually intermediate **C** was converted into the desired **3** via intermediate **D** along with the recycling of the Cu catalyst.

## Conclusion

In conclusion, Cu-catalyzed oxidative direct amidation from nonactivated carboxylic acid with benzimidazoles under dioxygen atmosphere with molecular oxygen as an activating reagent has been described. Azole amides were obtained in good yield in an oxidative protocol with a very broad range of substrates. Subsequent transamidation could be performed using the prepared azole amides and a variety of amines. This reaction has many advantages, in particular the use of an inexpensive copper salt as the catalyst, the use of oxygen as both the sole terminal oxidant and activating reagent, and inexpensive and readily available starting materials. This is the first Cu-catalyzed, direct, amide formation between nonactivated carboxylic acids and benzimidazoles in coupling reagent, traditional activating, reagent-free conditions. A mechanistic study demonstrated that a peroxycarboxylate was the key intermediate. This was the first reported example in such a simple system which could be employed in amide formation. Notably, the transamidation occurred smoothly between the azole amide and a variety of amines, thus providing versatile amides with our new strategy. Therefore, this is a very general method for azole amide formation from versatile carboxylic acids with potential application in organic synthesis.

## Supporting Information

File 1 Experimental procedures, analytical data and NMR spectra.
