# Supplementary material for: Molecular-oxygen-promoted Cu-catalyzed oxidative direct amidation of nonactivated carboxylic acids with azoles
Source: Beilstein J Org Chem. 2015 Nov 11;11:2158–65. doi: 10.3762/bjoc.11.233 (PMC4660991; doi:10.3762/bjoc.11.233)

Supporting Information

for

Molecular-oxygen-promoted Cu-catalyzed oxidative direct amidation of nonactivated carboxylic acids with azoles

Wen Ding[1], Shaoyu Mai[2] and Qiuling Song[1,3*]

Address: [1]Institute of Next Generation Matter Transformation, College of Chemical Engineering at Huaqiao Univeristy, P. R. China, [2]College of Materials Science at Huaqiao University, 668 Jimei Blvd, Xiamen, Fujian, 361021, P. R. China and [3]Beijing National Laboratory for Molecular Sciences, Beijing, 100190, P. R. China

Email: Qiuling Song - qsong@hqu.edu.cn.

* Corresponding author

Experimental procedures, analytical data and NMR spectra

Table of Contents

General information S2

The compatibility of reaction with other amines S2

Experiments with pyridine *N*-oxide as possible intermediate S2

Preparative scale of the reaction S3

Procedure and characterization data for amidation reactions S3

References S9

NMR spectra S10

General information

All experiments were conducted with a sealed pressure vessel. Flash column chromatography was performed over silica gel (200–300 mesh). ^{1}H NMR spectra were recorded on a Bruker AVIII-500M spectrometer, chemical shifts (in ppm) were referenced to $CDCl_3$ (δ = 7.26 ppm) or DMSO-d_6 (δ = 2.54 ppm) as an internal standard. ^{13}C NMR spectra were obtained by using the same NMR spectrometer and were calibrated with $CDCl_3$ (δ = 77.0 ppm) or DMSO-d_6 (δ = 40.45 ppm). Unless otherwise noted, materials obtained from commercial suppliers were used without further purification.

The compatibility of reaction with other amines

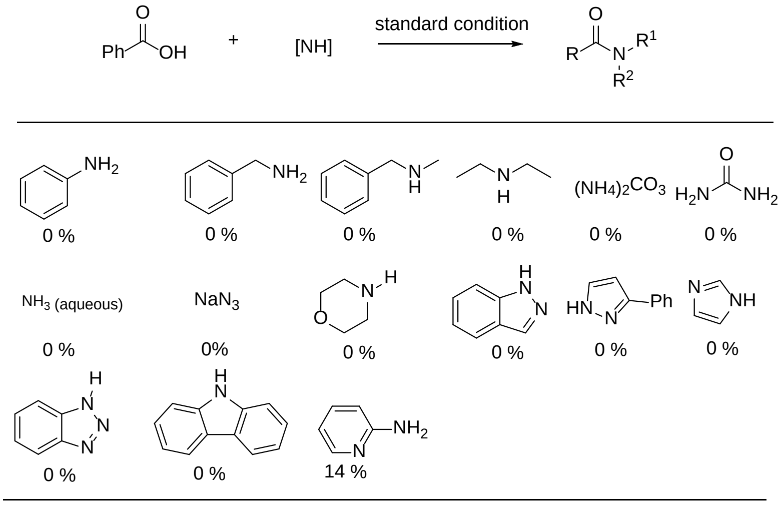

Scheme S1: The compatibility of reaction with other amines.

Experiments with pyridine *N*-oxide as possible intermediate

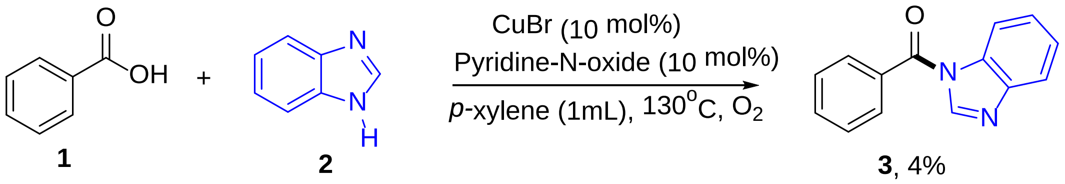

1 **2** **3**, 4%

A sealed pressure vessel was charged with benzoic acid (**1**, 30.0 mg, 0.25 mmol), benzimidazole (**2**, 60 mg, 0.5 mmol), CuBr (3.6 mg, 0.025 mmol), pyridine *N*-oxide (2.3 mg, 0.025 mmol), and *p*-xylene (1 mL). The resulting solution was stirred at 130 °C under O_2 for 20 hours. After cooling down to room temperature, the mixture was measured by GC without further purification. Most of starting materials remain, and only 4% yield of product **3** generated.

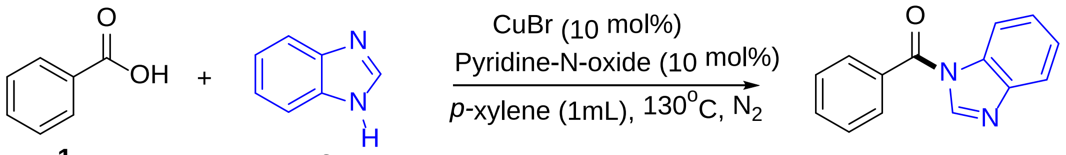

1 **2** **3**, 0%

A sealed pressure vessel was charged with benzoic acid (**1**, 30.0 mg, 0.25 mmol), benzimidazole (**2**, 60 mg, 0.5 mmol), CuBr (3.6 mg, 0.025 mmol), pyridine *N*-oxide (2.3 mg, 0.025 mmol), and *p*-xylene (1 mL). The resulting solution was stirred at 130 °C under N_2 for 20 hours. After cooling down to room temperature, the mixture was measured by GC without further purification. Starting materials remain, and no product generated at all.

Preparative scale of the reaction

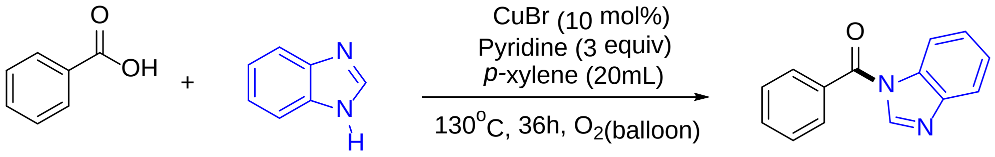

1 **2** **3**, 65%

A sealed pressure vessel was charged with benzoic acid (**1**, 610.0 mg, 5 mmol), benzimidazole (**2**, 1180 mg, 10 mmol), CuBr (71.7 mg, 0.5 mmol), pyridine (1200 mg, 15 mmol), and *p*-xylene (20 mL). The resulting solution was stirred at 130 °C under O_2 (balloon) monitored by TLC and GC for 32 hours. Upon completion of the reaction, the solvents were removed via rotary evaporator and the residue was purified with flash chromatography (silica gel, ethyl acetate/petroleum ether 1:4) to give 720 mg of (1*H*-benzo[*d*]imidazol-1-yl)(phenyl)methanone (**3**) in 65% isolated yield as a white solid.

Procedure and characterization data for amidation reactions

Typical procedure and characterization data for amidation reaction

A sealed pressure vessel was charged with benzoic acid (**1**, 30.0 mg, 0.25 mmol), benzimidazole (**2**, 60 mg, 0.5 mmol), CuBr (3.6 mg, 0.025 mmol), pyridine (60 mg, 0.75 mmol), and *p*-xylene (1 mL). The resulting solution was stirred at 130 °C under O_2 monitored by TLC and GC) for 16 hours. Upon completion of the reaction, the solvents were removed via rotary evaporator and the residue was purified with flash chromatography (silica gel, ethyl acetate/petroleum ether 1:4) to give 47 mg of (1*H*-benzo[*d*]imidazol-1-yl)(phenyl)methanone (**3**) in 85% isolated yield as a white solid.

(1*H*-Benzo[*d*]imidazol-1-yl)(phenyl)methanone (3, CAS：62573-86-8)[1]

85% yield (47 mg) as a white solid.. ^{1}H NMR: (500 MHz, $CDCl_3$, ppm) δ 8.22 (s, 1 H), 8.21 - 8.19 (m, 1 H), 7.85 - 7.83 (m, 1 H), 7.82 - 7.80 (m, 2 H), 7.71 - 7.68 (m, 1 H), 7.61 - 7.58 (m, 2 H), 7.47 - 7.42 (m, 2 H).; ^{13}C NMR: (125 MHz, $CDCl_3$, ppm).) δ 167.1 (s), 144.0 (s), 143.1 (s), 133.2 (s), 132.8 (s), 132.1 (s), 129.5 (s), 129.0 (s), 125.8 (s), 125.3 (s), 120.5 (s), 115.4 (s). mp = 70.1 - 71.2 ^{o}C.

(1*H*-Benzo[*d*]imidazol-1-yl)(*p*-tolyl)methanone (4, CAS：28997-00-4)[1]

75% yield (44 mg) as a white solid. ^{1}H NMR: (500 MHz, $CDCl_3$, ppm) δ 8.24 (s, 1 H), 8.19 - 8.17 (m, 1 H), 7.84 - 7.83 (m, 1 H), 7.72 -7.71(m, 2 H), 7.46 - 7.41 (m, 2 H), 7.39 - 7.38 (m, 2 H), 2.49 (s, 1 H).; ^{13}C NMR: (125 MHz, $CDCl_3$, ppm) δ 167.1 (s), 144.3 (s), 144.1 (s), 143.1 (s), 132.2 (s), 130.0 (s), 129.8 (s), 129.7 (s), 125.6 (s), 125.1 (s), 120.5 (s), 115.4 (s), 21.7 (s). mp = 92.7 - 94.5 ^{o}C.

(1*H*-Benzo[*d*]imidazol-1-yl)(4-ethylphenyl)methanone (5, new compound)

92% yield (57 mg) as a white solid. ^{1}H NMR: (500 MHz, $CDCl_3$, ppm) δ 8.25 (s, 1 H), 8.20 - 8.18 (m, 1 H), 7.85 - 7.83 (m, 1 H), 7.75 - 7.73 (m, 2 H), 7.47 - 7.40 (m, 4 H), 2.78 (q, *J*=8.00Hz, 2 H),

1.31 (t, J=7.5, 3 H).; ^{13}C NMR: (125 MHz, $CDCl_3$, ppm) δ 167.1 (s), 150.4 (s), 144.1 (s), 143.2 (s), 132.2 (s), 130.2 (s), 129.9 (s), 128.5 (s), 125.6 (s), 125.1 (s), 120.5 (s), 115.4 (s), 29.0 (s), 15.1 (s). new compound, HRMS m/z (EI) calcd. for C16H14N2O M+ 250.1106, found 250.1111. mp = 90.1 - 91.8 ^{o}C.

(1*H*-Benzo[*d*]imidazol-1-yl)(4-isopropylphenyl)methanone (6, CAS：901440-49-1)

71% yield (47 mg) as a white solid. ^{1}H NMR: (500 MHz, $CDCl_3$, ppm) δ 8.26 (s, 1 H), 8.21 - 8.19 (m, 1 H), 7.85 - 7.83 (m, 1 H), 7.76 - 7.74 (m, 2 H), 7.47 - 7.41 (m, 4 H), 3.08 - 2.99 (m, 1 H), 1.32 (d, J=7.00, 6 H).; ^{13}C NMR: (125 MHz, $CDCl_3$, ppm).) δ 167.0 (s), 154.9 (s), 144.0 (s), 143.2 (s), 132.2 (s), 130.3 (s), 129.9 (s), 127.2 (s), 125.6 (s), 125.1 (s), 120.5 (s), 115.4 (s), 34.3 (s), 23.6 (s). mp = 75.5 - 76.5 ^{o}C.

(1*H*-Benzo[*d*]imidazol-1-yl)(4-propylphenyl)methanone (7, new compound)[2]

64% yield (43 mg) as a white solid. ^{1}H NMR (500 MHz, $CDCl_3$) δ 8.27 (s, 1H), 8.23 - 8.15 (m, 1H), 7.89 - 7.80 (m, 1H), 7.77 - 7.65 (m, 2H), 7.49 - 7.41 (m, 2H), 7.39 (d, J = 8.2 Hz, 2H), 2.76 - 2.68 (m, 2H), 1.76 - 1.68 (m, 2H), 0.99 (t, J = 7.3 Hz, 3H).; ^{13}C NMR (126 MHz, $CDCl_3$) δ 167.1 (s), 149.0 (s), 149.0 (s), 143.2 (s), 132.2 (s), 130.2 (s), 129.8 (s), 129.1 (s), 125.7 (s), 125.2 (s), 120.5 (s), 115.4 (s), 38.0 (s), 24.2 (s), 13.8 (s). new compound, HRMS m/z (EI) calcd. for C17H16N2O M+ 264.1263, found 264.1262. mp = 53.8 - 54.9 ^{o}C.

(1*H*-Benzo[*d*]imidazol-1-yl)(4-(*tert*-butyl)phenyl)methanone (8, CAS：20208-57-5)

95% yield (66 mg) as a white solid. ^{1}H NMR: (500 MHz, $CDCl_3$, ppm) δ 8.26 (s, 1 H), 8.23 - 8.21 (m, 1 H), 7.85 - 7.83 (m, 1 H), 7.77 - 7.75 (m, 2 H), 7.61 - 7.59 (m, 2 H), 7.47 - 7.41 (m, 2 H), 1.39(s, 9 H).; ^{13}C NMR: (125 MHz, $CDCl_3$, ppm) δ 167.1 (s), 157.2 (s), 144.1 (s), 143.2 (s), 132.2 (s), 130.0 (s), 129.7 (s), 126.0 (s), 125.7 (s), 125.1 (s), 120.5 (s), 115.5 (s), 35.3 (s), 31.1 (s). mp = 131.3 - 132.8 ^{o}C.

(1*H*-Benzo[*d*]imidazol-1-yl)(4-butylphenyl)methanone (9, new compound)

67% yield (46 mg) as a white solid. ^{1}H NMR: (500 MHz, CDCl3, ppm) δ 8.27 (s, 1H), 8.19 (dd, J = 6.9, 2.0 Hz, 1H), 7.88 - 7.82 (m, 1H), 7.73 (d, J = 8.1 Hz, 2H), 7.44 (qd, J = 7.4, 6.0 Hz, 2H), 7.39 (d, J = 8.0 Hz, 2H), 3.00 (t, J = 7.5Hz, 2H), 1.68 - 1.65 (m, 2H), 1.42 - 1.38 (m, 2H), 0.96 (t, J = 7.4 Hz, 3H).; 13C NMR: (125 MHz, CDCl3, ppm). δ 167.1 (s), 149.2 (s), 144.0 (s), 143.2 (s), 132.2 (s), 130.1 (s), 129.8 (s), 129.1 (s), 125.7 (s), 125.1 (s), 120.5 (s), 115.4 (s), 35.7 (s), 33.2 (s), 22.3 (s), 13.9 (s). new compound , HRMS m/z (EI) calcd. for C18H18N2O M+ 278.1419, found 278.1411. mp = 37.1-38.9 ^{o}C.

(1*H*-Benzo[*d*]imidazol-1-yl)(4-fluorophenyl)methanone (10, CAS：154786-24-0)[4]

53% yield (32 mg) as a white solid. ^{1}H NMR: (500 MHz, $CDCl_3$, ppm) δ 8.21 (s, 1 H), 8.16 - 8.14 (m, 1 H), 7.87 - 7.83 (m, 3 H), 7.48 - 7.42 (m, 2 H), 7.30 - 7.27 (m, 2 H).; ^{13}C NMR: (125 MHz, $CDCl_3$, ppm) δ 165.9 (s), 165.6(d, J= 254.63Hz), 144.1 (s), 142.7 (s), 132.3(d, J= 9.13Hz), 132.1 (s), 129.5(d, J= 3.38Hz), 125.8 (s), 125.4 (s), 120.6 (s), 116.5(d, J= 22.13Hz), 115.3 (s). mp = 74.8 - 76.1 ^{o}C.

(1*H*-Benzo[*d*]imidazol-1-yl)(4-chlorophenyl)methanone (11, CAS：71589-37-2)[5]

52% yield (34 mg) as a white solid. ^{1}H NMR: (500 MHz, CDCl3, ppm) δ 8.19 (s, 1 H), 8.17 - 8.15 (m, 1 H), 7.85 - 7.83 (m, 1 H), 7.78 - 7.75 (m, 2 H), 7.59 - 7.57 (m, 2 H); 7.48 - 7.43 (m, 2H).; ^{13}C NMR: (125 MHz, CDCl3, ppm) δ 166.0 (s), 144.0 (s), 142.7 (s), 139.9 (s), 132.0 (s), 131.1 (s), 130.9 (s), 129.5 (s), 125.9 (s), 125.4 (s), 120.6 (s), 115.4 (s). mp = 149.3 - 150.9 ^{o}C.

(1*H*-Benzo[*d*]imidazol-1-yl)(4-bromophenyl)methanone (12, CAS：304668-33-5)[1]

40% yield (30 mg) as a white solid. ^{1}H NMR: (500 MHz, CDCl3, ppm) δ 8.18 (s, 1 H), 8.17 - 8.13

(m, 1 H), 7.85 - 7.81 (m, 1 H), 7.75 - 7.73 (m, 2 H), 7.69 - 7.66 (m, 2 H), 7.47-7.42 (m, 2H).; ^{13}C NMR: (125 MHz, CDCl3, ppm) δ 166.1 (s), 144.0 (s), 142.6 (s), 132.4 (s), 132.0 (s), 131.6 (s), 131.0 (s), 128.4 (s), 125.9 (s), 125.4 (s), 120.6 (s), 115.4 (s). mp = 108.9 - 110.3 ^{o}C.

(1*H*-Benzo[*d*]imidazol-1-yl)(4-methoxyphenyl)methanone (13, CAS: 13361-55-2)[3]

77% yield (48 mg) as a white solid. ^{1}H NMR (500 MHz, $CDCl_3$) δ 8.27 (s, 1H), 8.13 (dd, *J* = 6.5, 2.6 Hz, 1H), 7.86 - 7.78 (m, 3H), 7.46 - 7.40 (m, 2H), 7.06 (d, *J* = 8.8 Hz, 2H), 3.92 (s, 3H).; ^{13}C NMR (126 MHz, $CDCl_3$) δ 166.4 (s), 163.7 (s), 144.0 (s), 143.1 (s), 132.3 (s), 132.1 (s), 125.5 (s), 125.0 (s), 124.7 (s), 120.5 (s), 115.3 (s), 114.4 (s), 55.6 (s). mp = 111.9 - 113.7 ^{o}C.

(1*H*-Benzo[*d*]imidazol-1-yl)(4-vinylphenyl)methanone (14, CAS: 300396-82-1)

89% yield (54 mg) as a white solid. ^{1}H NMR (500 MHz, $CDCl_3$) δ 8.24 (s, 1H), 8.21 - 8.13 (m, 1H), 7.85 - 7.80 (m, 1H), 7.81 - 7.74 (m, 2H), 7.60 (d, *J* = 8.3 Hz, 2H), 7.47 - 7.40 (m, 2H), 6.80 (dd, *J* = 17.6, 10.9 Hz, 1H), 5.94 (d, *J* = 17.6 Hz, 1H), 5.47 (d, *J* = 10.9 Hz, 1H).; ^{13}C NMR (126 MHz, $CDCl_3$) δ 166.7 (s), 144.0 (s), 143.0 (s), 142.4 (s), 135.4 (s), 132.1 (s), 131.7 (s), 130.1 (s), 126.6 (s), 125.7 (s), 125.2 (s), 120.5 (s), 117.6 (s), 115.4 (s). mp = 95.8 - 96.6 ^{o}C.

(1*H*-Benzo[*d*]imidazol-1-yl)(4-cyclohexylphenyl)methanone (15, new compound)

78% yield (60 mg) as a white solid. ^{1}H NMR (500 MHz, $CDCl_3$) δ 8.26 (s, 1H), 8.20 (dd, *J* = 6.7, 2.0 Hz, 1H), 7.83 (dd, *J* = 6.7, 2.1 Hz, 1H), 7.74 (d, *J* = 8.2 Hz, 2H), 7.47 - 7.39 (m, 4H), 2.67 - 2.58 (m, 1H), 1.94 - 1.85 (m, 4H), 1.81 - 1.76 (m, 1H), 1.51 - 1.38 (m, 4H), 1.33 - 1.25 (m, 1H).; ^{13}C NMR (126 MHz, $CDCl_3$) δ 167.0 (s), 154.1 (s), 144.0 (s), 143.2 (s), 132.2 (s), 130.2 (s), 129.9 (s), 127.5 (s), 125.6 (s), 125.1 (s), 120.4 (s), 115.4 (s), 44.7 (s), 34.1 (s), 26.6 (s), 25.9 (s). new compound, HRMS m/z (EI) calcd. for C20H20N2O M+ 304.1576, found 304.1570. mp = 94.0 - 96.1 ^{o}C.

(1*H*-Benzo[*d*]imidazol-1-yl)(o-tolyl)methanone (16, CAS: 200626-52-4)

76% yield (45 mg) as a yellow oil. ^{1}H NMR (500 MHz, $CDCl_3$) δ 8.18 - 8.14 (m, 1H), 7.97 (s, 1H), 7.84 - 7.81 (m, 1H), 7.51 (td, *J* = 7.6, 1.3 Hz, 1H), 7.46 - 7.42 (m, 3H), 7.37 (dd, *J* = 8.9, 8.4 Hz, 2H), 2.40 (s, 3H).; ^{13}C NMR (126 MHz, $CDCl_3$) 13C NMR (126 MHz, CDCl3) δ 167.6 (s), 144.3 (s), 143.0 (s), 136.7 (s), 133.0 (s), 131.7(s), 131.6(s), 131.4 (s), 127.9 (s), 126.0 (s), 125.8 (s), 125.3 (s), 120.6 (s), 115.4 (s), 19.4 (s).

(1*H*-Benzo[*d*]imidazol-1-yl)(3,4-dimethylphenyl)methanone (17, CAS: 333348-55-3)

72% yield (45 mg) as a white solid. ^{1}H NMR (500 MHz, $CDCl_3$) δ 8.25 (s, 1H), 8.20 - 8.15 (m, 1H), 7.86 - 7.79 (m, 1H), 7.59 (s, 1H), 7.52 (dd, *J* = 7.8, 1.7 Hz, 1H), 7.46 - 7.39 (m, 2H), 7.32 (d, *J* = 7.8 Hz, 1H), 2.38 (s, 3H), 2.35 (s, 3H).; ^{13}C NMR (126 MHz, $CDCl_3$) δ 167.2 (s), 144.0 (s), 143.2 (s), 143.0 (s), 137.7 (s), 132.2 (s), 130.7 (s), 130.3 (s), 130.1 (s), 127.3 (s), 125.5 (s), 125.0 (s), 120.4 (s), 115.4 (s), 20.0 (s), 19.7 (s). mp = 122.1 - 123.3 ^{o}C.

(1*H*-Benzo[*d*]imidazol-1-yl)(3,4-dichlorophenyl)methanone (18, CAS: 330215-63-9)

28% yield (21 mg) as a white solid.. ^{1}H NMR (500 MHz, $CDCl_3$) δ 8.19 (s, 1H), 8.18 - 8.14 (m, 1H), 7.93 (d, *J* = 2.0 Hz, 1H), 7.87 - 7.83 (m, 1H), 7.69 (d, *J* = 8.3 Hz, 1H), 7.64 (dd, *J* = 8.3, 2.0 Hz, 1H), 7.49 - 7.44 (m, 2H).; ^{13}C NMR (126 MHz, $CDCl_3$) δ 164.7 (s), 144.0 (s), 142.4 (s), 138.2 (s), 134.0 (s), 132.4 (s), 131.9 (s), 131.4 (s), 131.2 (s), 128.4 (s), 126.1 (s), 125.7 (s), 120.8 (s), 115.4 (s). mp = 131.8 - 133.7 ^{o}C.

(1*H*-Benzo[*d*]imidazol-1-yl)(3-chlorophenyl)methanone (19, CAS :200626-53-5)

51% yield (26 mg) as a white solid. ^{1}H NMR (500 MHz, $CDCl_3$) δ 8.23 (s, 1H), 8.21 - 8.18 (m, 1H), 7.86 - 7.83 (m, 1H), 7.82 - 7.79 (m, 2H), 7.72 - 7.68 (m, 1H), 7.61 - 7.57 (m, 2H), 7.48 - 7.41 (m, 2H).; ^{13}C NMR (126 MHz, $CDCl_3$) δ 167.1 (s), 144.0 (s), 143.1 (s), 133.2 (s), 132.8 (s), 132.1

(s), 129.5 (s), 129.0 (s), 125.8 (s), 125.3 (s), 120.5 (s), 115.5 (s). mp = 102.3 - 104.1 ^{o}C.

(1*H*-Benzo[*d*]imidazol-1-yl)(naphthalen-2-yl)methanone (20, new compound)

59% yield (40 mg) as a white solid. ^{1}H NMR: (500 MHz, CDCl3, ppm) δ 8.33 - 8.32 (m, 2 H), 8.23 - 8.22 (m, 1 H), 8.05 - 8.04 (m, 1 H), 7.98 - 7.96 (m, 2 H), 7.88 - 7.86 (m, 2 H); 7.70 - 7.67(m, 1H), 7.65 - 7.62 (m, 1H), 7.49 - 7.44 (m, 2H).; ^{13}C NMR: (125 MHz, CDCl3, ppm). δ 167.2 (s), 144.0 (s), 143.2 (s), 135.3 (s), 132.2 (s), 131.1 (s), 129.9 (s), 129.2 (s), 129.0 (s), 128.0 (s), 127.6 (s), 125.8 (s), 125.3 (s), 125.0 (s), 120.6 (s), 115.4 (s). new compound, HRMS m/z (EI) calcd. for C18H12N2O M+ 272.0950, found 272.0953. mp = 105.1 - 106.7 ^{o}C.

(1*H*-Benzo[*d*]imidazol-1-yl)(pyren-4-yl)methanone (21, new compound)

88% yield (76 mg) as a white solid. ^{1}H NMR (500 MHz, $CDCl_3$) δ 8.32 - 8.20 (m, 6H), 8.17 – 8.06 (m, 4H), 8.01 (s, 1H), 7.91 - 7.86 (m, 1H), 7.51 - 7.46 (m, 2H).; ^{13}C NMR (126 MHz, $CDCl_3$) δ 167.6 (s), 144.3 (s), 143.5 (s), 133.8 (s), 132.0 (s), 131.0 (s), 130.4 (s), 130.0 (s), 129.9 (s), 129.6 (s), 126.9 (s), 126.8 (s), 126.7 (s), 126.5 (s), 126.5 (s), 126.0 (s), 125.9 (s), 125.4 (s), 124.6 (s), 124.1 (s), 124.0 (s), 123.2 (s), 120.6 (s), 115.6 (s). new compound, HRMS m/z (EI) calcd. for C24H14N2O M+ 346.1106, found 346.1103. mp = 132.5 - 134.3 ^{o}C.

(1*H*-Benzo[*d*]imidazol-1-yl)(thiophen-3-yl)methanone (22, new compound)

40% yield (24 mg) as a white solid. ^{1}H NMR: (500 MHz, CDCl3, ppm) δ 8.404 (s, 1 H), 8.217 - 8.199 (m, 1 H), 8.074 - 8.066 (m, 1 H), 7.851 - 7.834 (m, 1 H), 7.586 - 7.574 (m, 1 H), 7.542 - 7.526 (m, 1H), 7.475 - 7.416 (m, 2H).; ^{13}C NMR: (125 MHz, CDCl3, ppm) δ 161.5 (s), 144.0 (s), 142.5 (s), 134.7 (s), 133.2 (s), 132.1 (s), 128.2 (s), 127.6 (s), 125.7 (s), 125.3 (s), 120.6 (s), 115.4 (s). new compound), HRMS m/z (EI) calcd. for C12H8N2OS M+ 228.0357, found 228.0361. mp = 124.8 - 126.3 ^{o}C.

(1*H*-Benzo[*d*]imidazol-1-yl)(furan-3-yl)methanone (23, new compound)

61% yield (33 mg) as a white solid. ^{1}H NMR (500 MHz, $CDCl_3$) δ 8.48 (s, 1H), 8.26 - 8.22 (m, 1H), 8.15 - 8.10 (m, 1H), 7.84 (dd, *J* = 6.8, 1.8 Hz, 1H), 7.62 (t, *J* = 1.7 Hz, 1H), 7.48 - 7.42 (m, 2H), 6.91 (dd, *J* = 1.9, 0.8 Hz, 1H).; ^{13}C NMR (126 MHz, $CDCl_3$) δ 160.4 (s), 147.7 (s), 144.6 (s), 143.8 (s), 141.9 (s), 131.9 (s), 125.9 (s), 125.3 (s), 120.8 (s), 120.5 (s), 115.4 (s), 110.4 (s). new compound, HRMS m/z (EI) calcd. for C12H8N2O2 M+ 212.0586, found 212.0589. mp = 128.3 - 130.1 ^{o}C.

(1*H*-Benzo[*d*]imidazol-1-yl)(cyclohexyl)methanone (24, CAS: 294649-09-5)

51% yield (30 mg) as a white solid. ^{1}H NMR (500 MHz, $CDCl_3$) δ 8.44 (s, 1H), 8.28 - 8.22 (m, 1H), 7.80 (dd, *J* = 7.2, 1.3 Hz, 1H), 7.45 - 7.35 (m, 2H), 3.06 (tt, *J* = 11.5, 3.4 Hz, 1H), 2.04 (dd, *J* = 14.1, 1.6 Hz, 2H), 1.96 - 1.87 (m, 2H), 1.81 - 1.70 (m, 3H), 1.48 - 1.39 (m, 2H), 1.37 - 1.30 (m, 1H).; ^{13}C NMR (126 MHz, $CDCl_3$) δ 173.8 (s), 143.7 (s), 140.8 (s), 131.6 (s), 125.9 (s), 125.0 (s), 120.4 (s), 115.8 (s), 44.1 (s), 29.4 (s), 25.5 (s), 25.4 (s). mp = 57.8 - 59.1 ^{o}C.

1-(1*H*-Benzo[*d*]imidazol-1-yl)hexan-1-one (25, CAS: 901547-84-0)

47% yield (26 mg) as a yellow oil. ^{1}H NMR (500 MHz, $CDCl_3$) δ 8.41 (s, 1H), 8.29 - 8.21 (m, 1H), 7.80 (dd, *J* = 7.1, 1.4 Hz, 1H), 7.47 - 7.35 (m, 2H), 3.00 (t, *J* = 7.4 Hz, 2H), 1.91 - 1.84 (m, 2H), 1.48 - 1.36 (m, 4H), 0.94 (t, *J* = 7.1 Hz, 3H).; ^{13}C NMR (126 MHz, $CDCl_3$) δ 170.4 (s), 143.8 (s), 140.9 (s), 131.5 (s), 125.9 (s), 125.0 (s), 120.5 (s), 115.6 (s), 35.9 (s), 31.2 (s), 24.0 (s), 22.4 (s), 13.9 (s).

1-(1*H*-Benzo[*d*]imidazol-1-yl)ethan-1-one (26, CAS: 18773-95-0)[8]

46% yield (19 mg) as a white solid. ^{1}H NMR (500 MHz, $CDCl_3$) δ 8.36 (s, 1H), 8.27 - 8.16 (m, 1H), 7.80 (dd, *J* = 7.0, 1.6 Hz, 1H), 7.45 - 7.38 (m, 2H), 2.74 (s, 3H).; ^{13}C NMR (126 MHz,

$CDCl_3$) δ 167.2 (s), 143.9 (s), 141.3 (s), 131.4 (s), 125.9 (s), 125.1 (s), 120.5 (s), 115.5 (s), 23.8 (s). mp = 92.0 - 93.4 ^{o}C.

(5,6-Dimethyl-1*H*-benzo[*d*]imidazol-1yl)(phenyl)methanone (27, CAS：16109-46-9)[7]

89% yield (57 mg) as a white solid. ^{1}H NMR: (500 MHz, $CDCl_3$, ppm) δ 8.09 (s, 1 H), 8.00 (s, 1 H), 7.80 - 7.78 (m, 2 H), 7.69 - 7.66 (m, 1 H), 7.59 - 7.56 (m, 3 H), 2.42(s, 3H), 2.40(s, 3H).; ^{13}C NMR: (125 MHz, $CDCl_3$, ppm). δ 167.1 (s), 142.5 (s), 142.4 (s), 135.1 (s), 134.3 (s), 133.9 (s), 133.0 (s), 130.5 (s), 129.5 (s), 129.0 (s), 120.5 (s), 115.7 (s), 20.5 (s), 20.3 (s). mp = 91.8 - 93.7 ^{o}C.

Typical procedure and characterization data for amine exchange reaction

A sealed pressure vessel was charged with (1*H*-benzo[*d*]imidazol-1-yl)(phenyl)methanone (**3**) (55.0 mg, 0.25 mmol), pyridin-2-amine (**27**, 35.5 mg, 0.375 mmol), and *p*-xylene (1 mL). The resulting solution was stirred at 130 °C under air overnight. Upon completion of the reaction, the solvents were removed via rotary evaporator and the residue was purified with flash chromatography (silica gel, ethyl acetate/petroleum ether 1:4) to give 47 mg of *N*-(pyridin-2-yl)benzamide (**29**) in 92% isolated yield as a yellow solid.

***N*-(Pyridin-2-yl)benzamide (29, CAS : 4589-12-2)[9]**

92% yield (47 mg) as a yellow solid.^{1}H NMR (500 MHz, $CDCl_3$) δ 8.84 (s, 1H), 8.40 (d, *J* = 8.4 Hz, 1H), 8.23 (d, *J* = 4.1 Hz, 1H), 7.98 - 7.88 (m, 2H), 7.81 - 7.69 (m, 1H), 7.57 (t, *J* = 7.4 Hz, 1H), 7.49 (t, *J* = 7.6 Hz, 2H), 7.11 - 6.99 (m, 1H).; ^{13}C NMR (126 MHz, $CDCl_3$) δ 165.8 (s), 151.6 (s), 147.9 (s), 138.5 (s), 134.3 (s), 132.2 (s), 128.8 (s), 127.2 (s), 119.9 (s), 114.2 (s). mp = 78.2 - 81.1 ^{o}C.

***N*-(Pyridin-2-yl)-2-naphthamide (31, CAS : 159257-88-2)[9]**

85% yield (53 mg) as a white solid. 1H NMR (500 MHz, CDCl3) δ 9.15 (s, 1H), 8.49 - 8.40 (m, 2H), 8.23 (ddd, *J* = 4.9, 1.7, 0.7 Hz, 1H), 7.99 (dd, *J* = 8.5, 1.8 Hz, 1H), 7.94 - 7.87 (m, 3H), 7.76 (ddd, *J* = 8.4, 7.4, 1.9 Hz, 1H), 7.57 (dtd, *J* = 14.7, 6.9, 1.3 Hz, 2H), 7.04 (ddd, *J* = 7.3, 4.9, 0.9 Hz, 1H).; ^{13}C NMR (126 MHz, $CDCl_3$) δ 165.9 (s), 151.7 (s), 147.8 (s), 138.5 (s), 135.0 (s), 132.5 (s), 131.4 (s), 129.0 (s), 128.7 (s), 128.0 (d, *J* = 9.8 Hz), 127.7 (s), 126.9 (s), 123.6 (s), 119.9 (s), 114.3 (s). mp = 162.2 - 163.1 ^{o}C.

(1*H*-Indazol-1-yl)(phenyl)methanone (32, CAS: 23301-00-0)[1]

95% yield (52 mg) as a white solid. ^{1}H NMR: (500 MHz, $CDCl_3$, ppm) δ 8.58 (dd, *J*= 8.5Hz, *J*= 0.7Hz, 1 H), 8.21 (d, *J*= 0.6Hz, 1 H), 8.09-8.07 (m, 2 H), 7.79 -7.77(m, 1 H), 7.64– 7.59 (m, 2 H), 7.55- 7.51(m, 2H), 7.43- 7.40(m, 1H); ^{13}C NMR: (125 MHz, $CDCl_3$, ppm).) δ 168.3 (s), 140.2 (s), 140.1 (s), 133.2 (s), 132.2 (s), 130.9 (s), 129.4 (s), 127.9 (s), 126.1 (s), 124.8 (s), 120.9 (s), 115.8 (s). mp = 83.8–84.2 ^{o}C.

(1*H*-Benzo[*d*][1,2,3]triazol-1-yl)(phenyl)methanone (33, CAS: 4231-62-3)[1]

56% yield (43 mg) as a white solid. ^{1}H NMR (500 MHz, DMSO-d_6) δ 8.27 (d, *J* = 8.3 Hz, 1H), 8.23 (d, *J* = 8.3 Hz, 1H), 8.06 (dd, *J* = 8.3, 1.2 Hz, 2H), 7.80 (ddd, *J* = 8.2, 7.2, 0.9 Hz, 1H), 7.78 – 7.70 (m, 1H), 7.67 – 7.51 (m, 3H); ^{13}C NMR (126 MHz, DMSO-d_6) δ 167.5 (s), 146.0 (s), 134.6 (s), 132.6 (s), 132.2 (d, *J* = 11.0 Hz), 131.7 (s), 129.3 (s), 127.7 (s), 120.9 (s), 115.3 (s). mp 111–112 ^{o}C.

***N*-Phenylbenzamide (34, CAS: 93-98-1)[10]**

68% yield (34 mg) as a white solid. ^{1}H NMR (500 MHz, DMSO-d_6) δ 10.26 (s, 1H), 7.99 – 7.84 (m, 2H), 7.73 (d, *J* = 7.6 Hz, 2H), 7.57 (ddd, *J* = 6.4, 3.7, 1.2 Hz, 1H), 7.50 (dd, *J* = 10.3, 4.6 Hz,

2H), 7.34 (t, J = 7.9 Hz, 2H), 7.10 (t, J = 7.4 Hz, 1H); ^{13}C NMR (126 MHz, DMSO-d_6) δ 166.8 (s), 139.8 (s), 135.7 (s), 132.6 (s), 129.6 (s), 129.4 (s), 128.5 (s), 124.8 (s), 121.5 (s). mp 161–163 ^{o}C.

***N*-Benzyl-*N*-methylbenzamide (35, CAS: 61802-83-3)[10]**

78% yield (57 mg) as a yellow oil. ^{1}H NMR (500 MHz, $CDCl_3$) δ7.47-7.29 (m, 9H), 7.17 (s, 1H), 4.77 (s, 1H), 4.51(s, 1H), 3.03 (s, 1.5H), 2.86(s, 1.5H); ^{13}C NMR (126 MHz, $CDCl_3$) δ172.3 (s), 171.6 (s), 137.0 (s), 136.5 (s), 136.1 (s), 129.6 (s), 128.8 (s), 128.7 (s), 128.3 (s), 128.1 (s), 127.5 (s), 126.9 (s), 126.7 (s), 55.1 (s), 50.7 (s), 36.9 (s), 33.1 (s).

Phenyl(3-phenyl-1*H*-pyrazol-1-yl)methanone (36, CAS: 126382-89-6)[1]

71% yield (44 mg) as a white solid. ^{1}H NMR (500 MHz, $CDCl_3$) δ 8.48 (d, J = 2.9 Hz, 1H), 8.31 – 8.23 (m, 2H), 7.91 – 7.87 (m, 2H), 7.68 – 7.60 (m, 1H), 7.54 (dd, J = 10.7, 4.8 Hz, 2H), 7.46 – 7.39 (m, 3H), 6.87 (d, J = 2.9 Hz, 1H); ^{13}C NMR: (125 MHz, $CDCl_3$, ppm).) δ 166.11 (s), 155.89 (s), 132.98 (s), 131.86 (s), 131.79 (s), 131.73 (s), 131.48 (s), 129.18 (s), 128.74 (s), 128.02 (s), 126.37 (s), 107.18 (s). mp = 63.7-64.9 ^{o}C.

***N*-(Pyridin-2-yl)cyclohexanecarboxamide (37, CAS: 68134-77-0)[11]**

86% yield (44 mg) as a white solid. ^{1}H NMR (500 MHz, $CDCl_3$) δ 8.71 (s, 1H), 8.29 – 8.15 (m, 2H), 7.73 – 7.60 (m, 1H), 7.06 – 6.90 (m, 1H), 2.24 (tt, J = 11.7, 3.5 Hz, 1H), 1.95 – 1.88 (m, 2H), 1.82 – 1.76 (m, 2H), 1.66 (dd, J = 9.0, 3.3 Hz, 1H), 1.52 (qd, J = 12.3, 3.2 Hz, 2H), 1.28 – 1.20 (m, 3H); ^{13}C NMR (126 MHz, $CDCl_3$) δ 175.0 (s), 151.8 (s), 147.4 (s), 138.4 (s), 119.5 (s), 114.3 (s), 46.3 (s), 29.4 (s), 25.6 (s), 25.5 (s). mp = 88.1- 89.3 ^{o}C.

Phenyl(piperidin-1-yl)methanone (38, CAS: 776-75-0)[10]

74% yield (35 mg) as a yellow oil. ^{1}H NMR (500 MHz, $CDCl_3$) δ 7.42 – 7.32 (m, 5H), 3.70 – 3.33 (m, 4H), 1.66 – 1.50 (m, 6H); ^{13}C NMR (126 MHz, $CDCl_3$) δ 170.3 (s), 136.5 (s), 129.3 (s), 128.3 (s), 126.7 (s), 48.7 (s), 43.1 (s), 26.5 (s), 25.6 (s), 24.5 (s).

***N,N*-Diethylbenzamide (39, CAS: 1696-17-9)[12]**

68% yield (30 mg) as a yellow oil.. ^{1}H NMR (500 MHz, $CDCl_3$) δ 7.41 – 7.31 (m, 5H), 3.54 – 3.24 (m, 4H), 1.27 – 1.05 (m, 6H); ^{13}C NMR (126 MHz, $CDCl_3$) δ 171.2 (s), 137.2 (s), 129.0 (s), 128.3 (s), 126.2 (s), 43.2 (s), 39.2 (s), 14.1 (s), 12.9 (s).

Methyl benzoyl-L-leucinate (40, CAS: 3005-60-5)[13]

A sealed pressure vessel was charged with (1*H*-benzo[*d*]imidazol-1-yl)(phenyl)methanone (**3**) (55.0 mg, 0.25 mmol), L-leucine methyl ester hydrochloride (68 mg, 0.375 mmol), Et_3N (38 mg, 0.375 mmol), and *p*-xylene (1 mL). The mixture was stirred at room temperature for 3 minutes. Then the resulting solution was heated to 130 °C under air overnight. Upon completion of the reaction, the solvents were removed via rotary evaporator and the residue was purified with flash chromatography (silica gel, ethyl acetate/petroleum ether 1:6) to give 45 mg of methyl benzoyl-L-leucinate (40) in 71% isolated yield as a white solid. ^{1}H NMR (500 MHz, $CDCl_3$) δ 7.85 – 7.76 (m, 2H), 7.56 – 7.39 (m, 3H), 6.64 (d, J = 8.0 Hz, 1H), 4.93 – 4.83 (m, 1H), 3.78 (s, 3H), 1.80 – 1.65 (m, 3H), 1.00 (d, J = 9.6, 3H), 0.99 (d, J = 9.6, 3H); ^{13}C NMR (126 MHz, $CDCl_3$) δ 173.7 (s), 167.1 (s), 133.9 (s), 131.7 (s), 128.5 (s), 127.0 (s), 52.3 (s), 51.1 (s), 41.8 (s), 25.0 (s), 22.8 (s), 22.0 (s).

References

(1). Zhao, J.; Li, P.; Xia, C.; Li, F., *Chem Commun.*, **2014**, 50, 4751-4754. doi: 10.1039/C4CC01587H

(2). Staab, H- A.; Lauer, D., *Chemische Berichte*., **1968**, 101, 864-878. doi: 10.1002/cber.19681010317

(3). Du, J.; Wang, X.; Zheng, R., *J Chem Res.*, **2007**, 14-15. doi:10.3184/030823407780199568

(4). Jois, Y. H. R.; Gibson, H. W., *Macromolecules.*, **1994**, 27, 2912-2916. doi: 10.1021/ma00089a003

(5). Hevener, K. E.; Mehboob, S.; Su, P-C.; Truong, K.; Boci, T.; Deng, J.; Ghassemi, M.; Cook, J. L.; Johnson, M. E.**,** *J Med Chem.*, **2012**, 55, 268-279. doi: 10.1021/jm201168g

(6). Mannschreck, A.; Staab, H. A.; Wurmb-Gerlich, D., *Tetrahedron Lett.*, **1963**,29, 2003-2010. doi:10.1016/S0040-4039(00)87820-X

(7). Yu, L.; Wang, M.; Wang, L., *Tetrahedron.*, **2014**, 70, 5391-5397. doi:10.1016/j.tet.2014.07.009

(8). Kim, B.R.; Sung, G. H.; Ryu, K. E.; Yoon, H. J.; Lee, S-G.; Yoon, Y-J., *Synlett*, **2014**, 25, 1909-1915. doi:10.1055/s-0034-1378335

(9). Subramanian, P.; Indu, S.; Kaliappan, K. P., *Org Lett.*, **2014**, 16, 6212-6215. doi: 10.1021/ol5031266

(10). Kovalenko, O. O.; Volkov, A.; Adolfsson, H., *Org Lett.*, **2015**, 17, 446-449. doi: 10.1021/ol503430t

(11). Itsenko, O.; Kihlberg.T.; Långström, B., *J. Org. Chem.*, **2004**, 69, 4356-4360. doi: 10.1021/jo049934m

(12). Xiong, B.; Zhu, L.; Feng, X.; Lei, J.; Chen, T.; Zhou, Y.; Han, L-B.; Au, C-T.; Yin, S-F., *Eur. J. Org. Chem*,, **2014**, 20, 4244-4247. doi: 10.1002/ejoc.201402332

(13). Reddy, K. R.; Maheswari, C. U.; Venkateshwar, M.; Kantam, M. L., *Eur. J. Org. Chem*, **2008**, 21, 3619-3622. doi: 10.1002/ejoc.200800454

NMR Spectra

(1*H*-Benzo[*d*]imidazol-1-yl)(phenyl)methanone（CAS：62573-86-8）

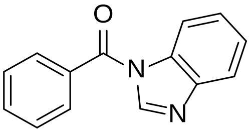

3

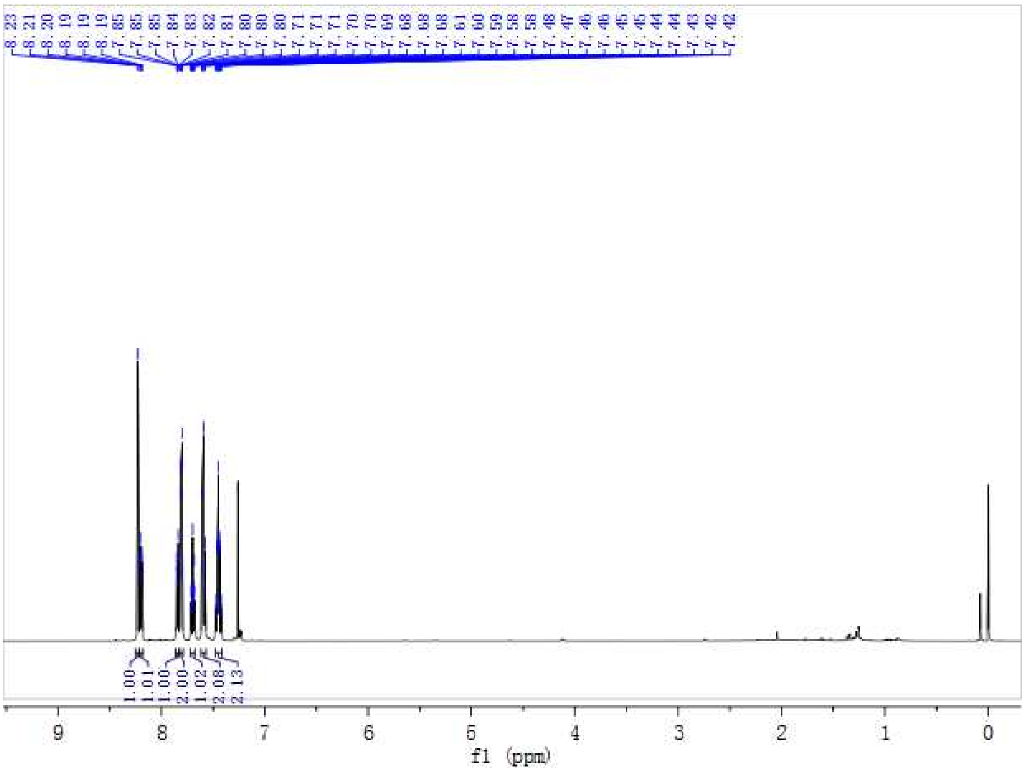

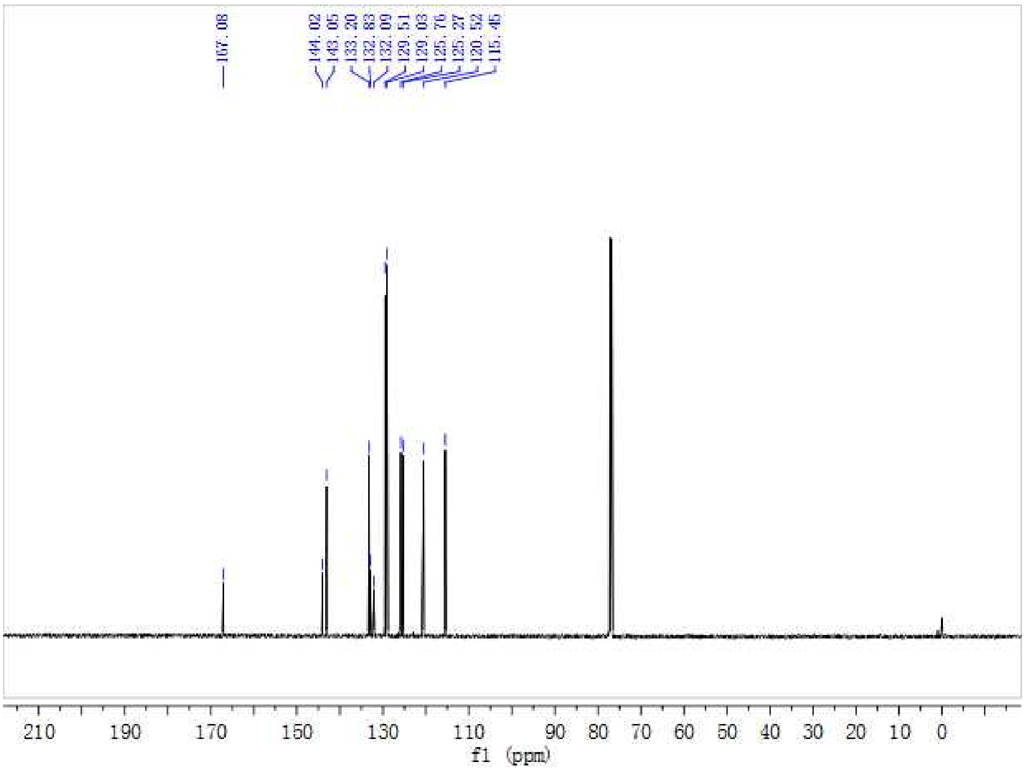

(1*H*-Benzo[*d*]imidazol-1-yl)(*p*-tolyl)methanone（CAS：28997-00-4）

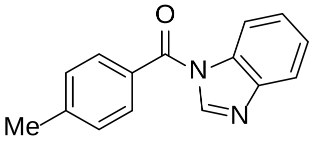

4

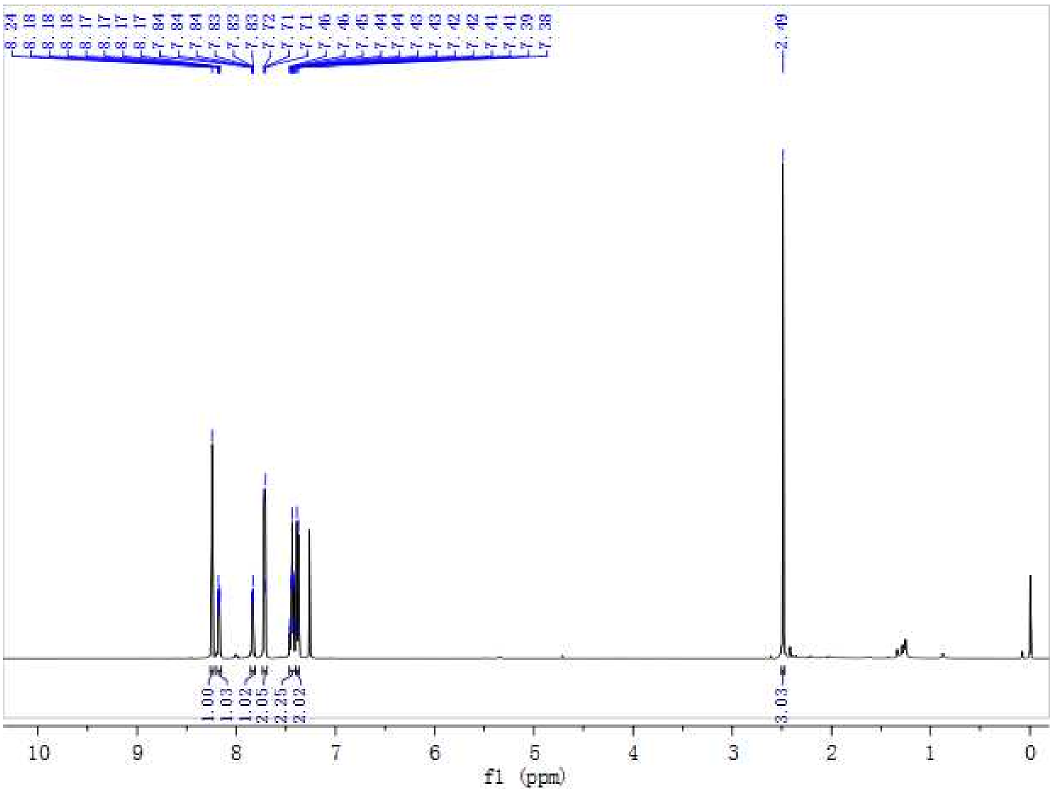

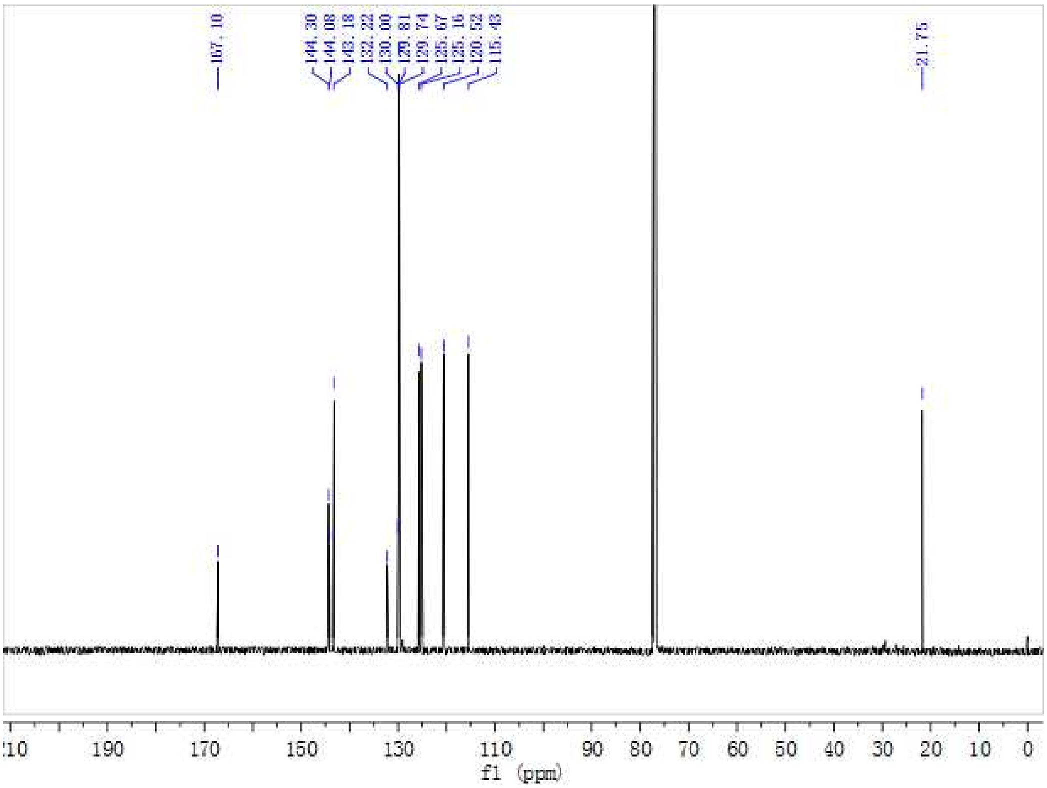

(1*H*-Benzo[*d*]imidazol-1-yl)(4-ethylphenyl)methanone (new compound)

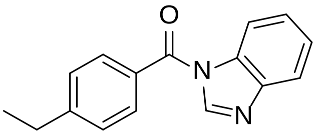

5

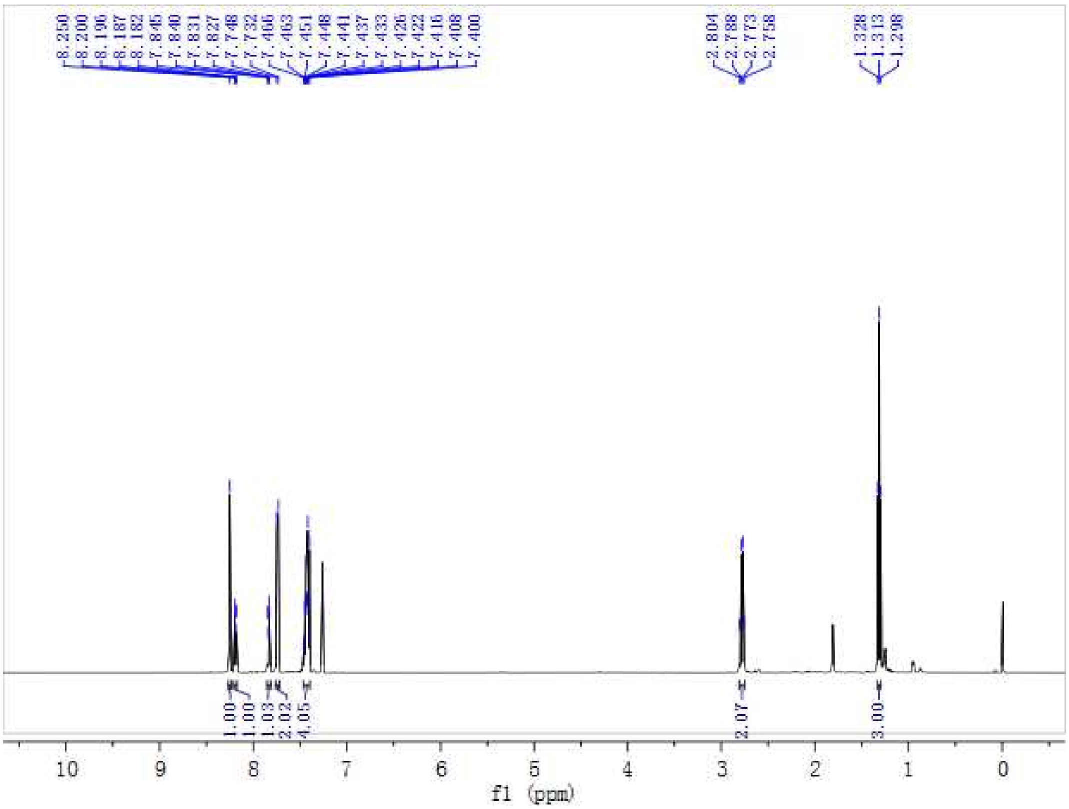

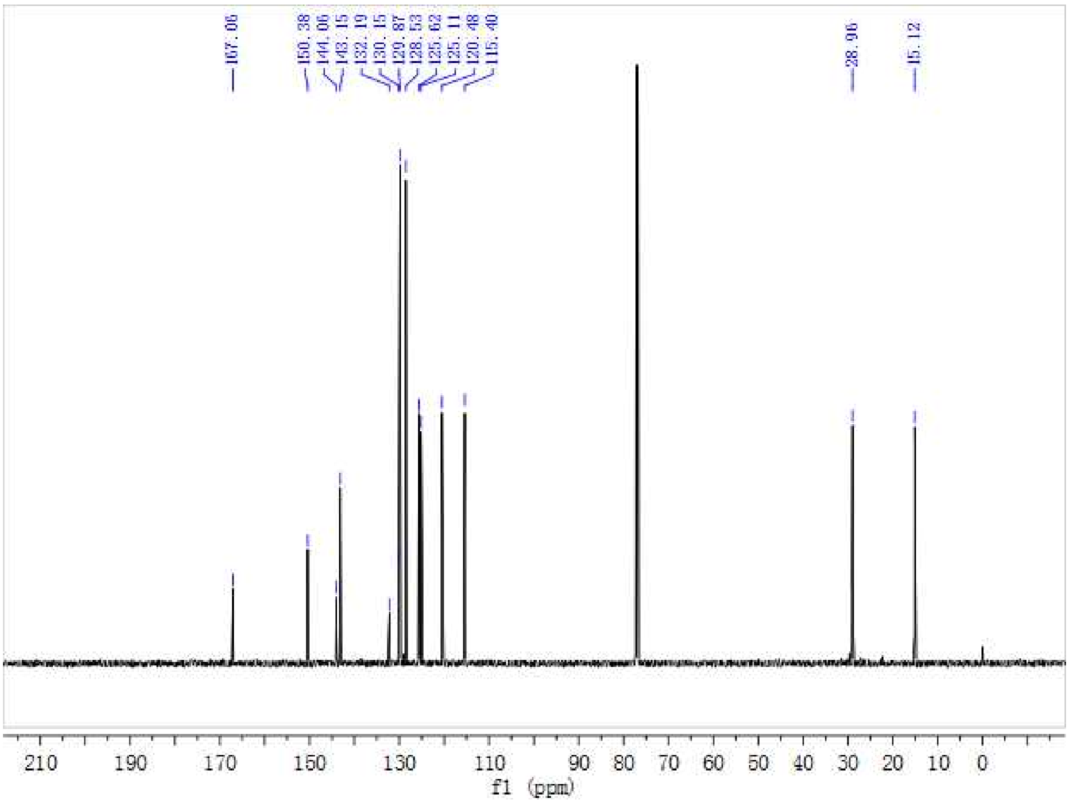

(1*H*-Benzo[*d*]imidazol-1-yl)(4-isopropylphenyl)methanone（CAS：901440-49-1）

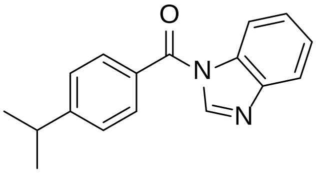

6

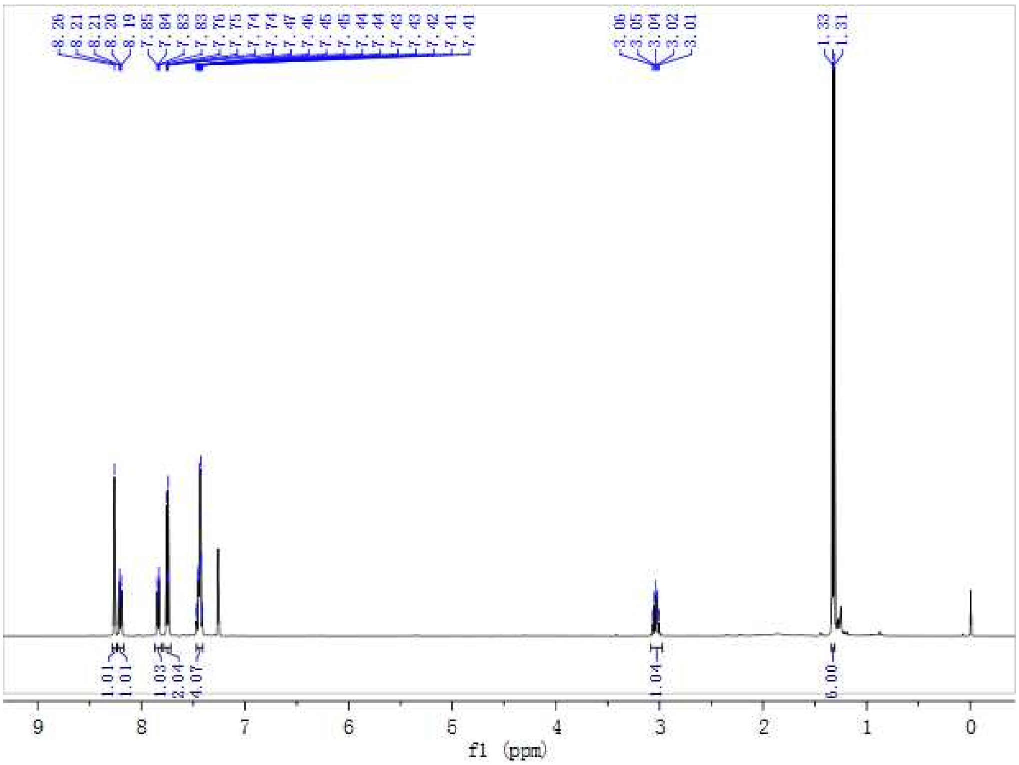

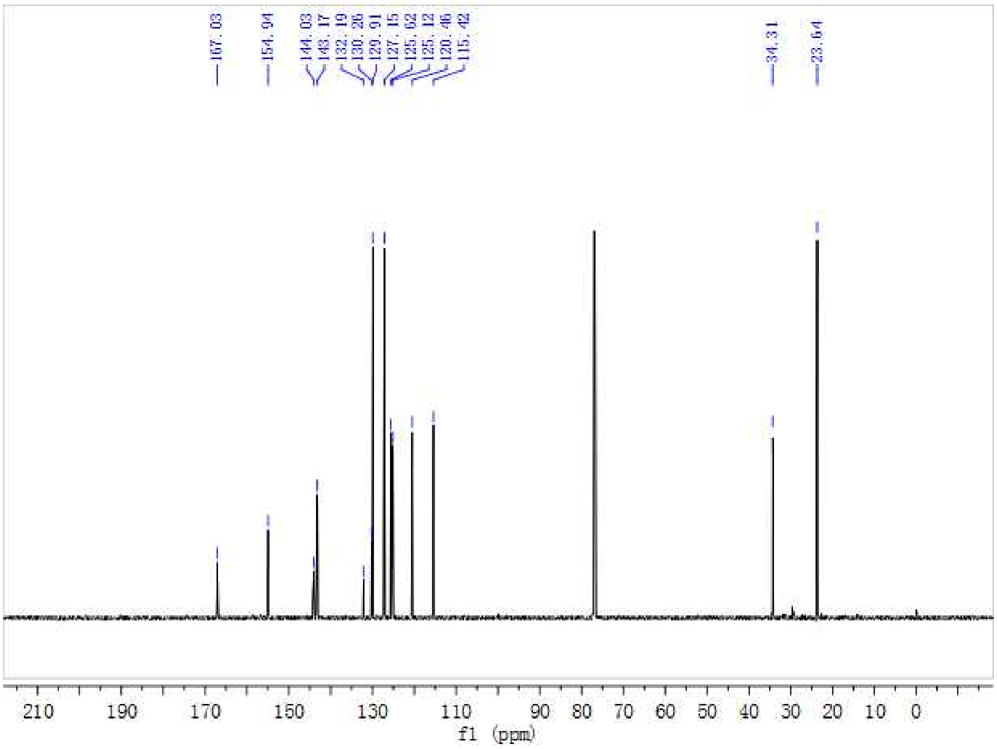

(1*H*-Benzo[*d*]imidazol-1-yl)(4-propylphenyl)methanone (new compound)

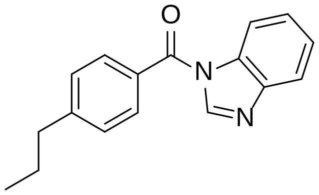

7

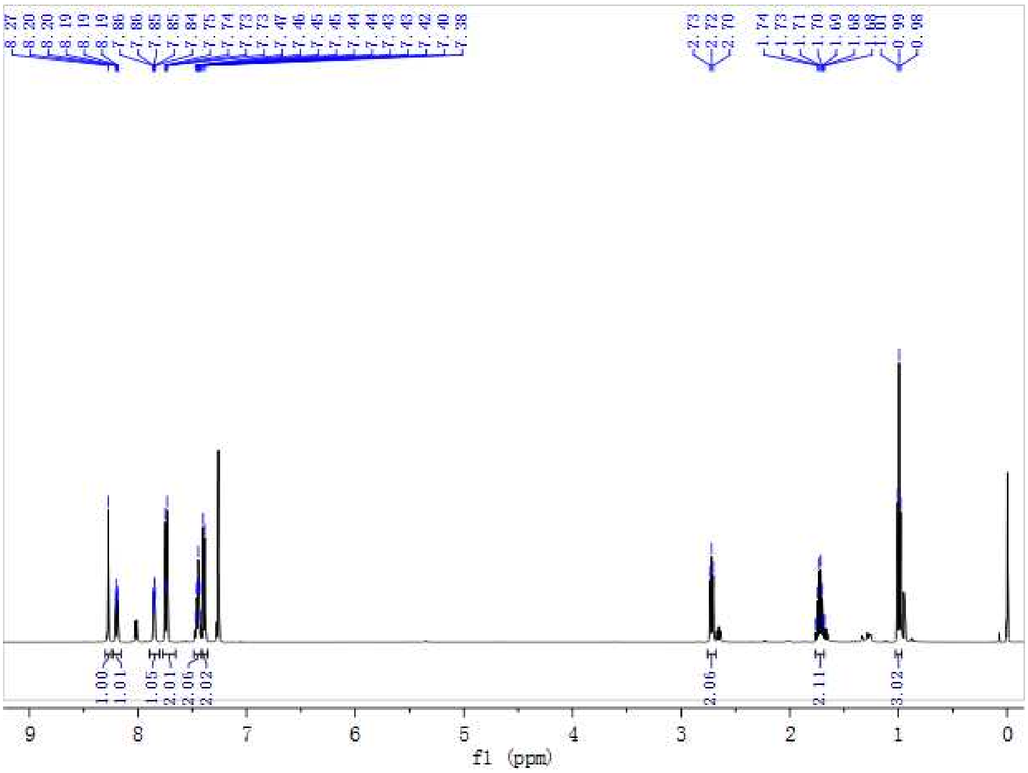

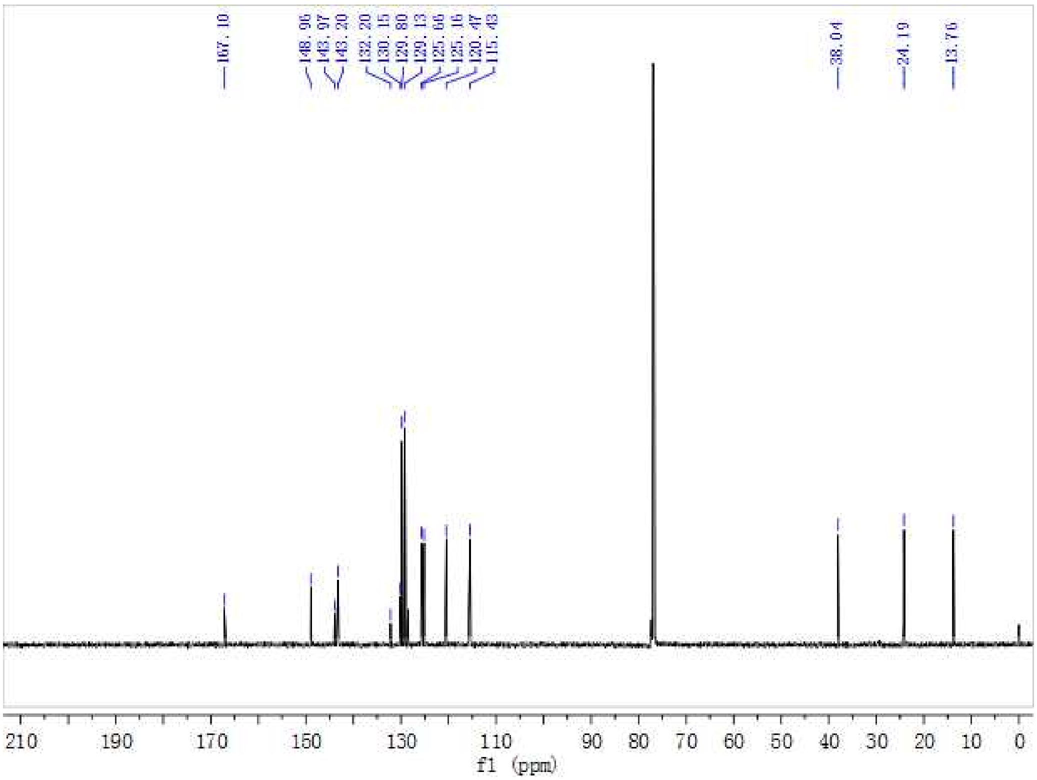

(1*H*-Benzo[*d*]imidazol-1-yl)(4-(*tert*-butyl)phenyl)methanone（CAS：20208-57-5）

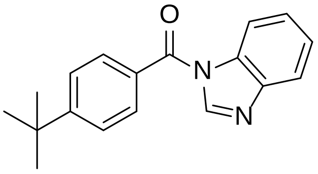

8

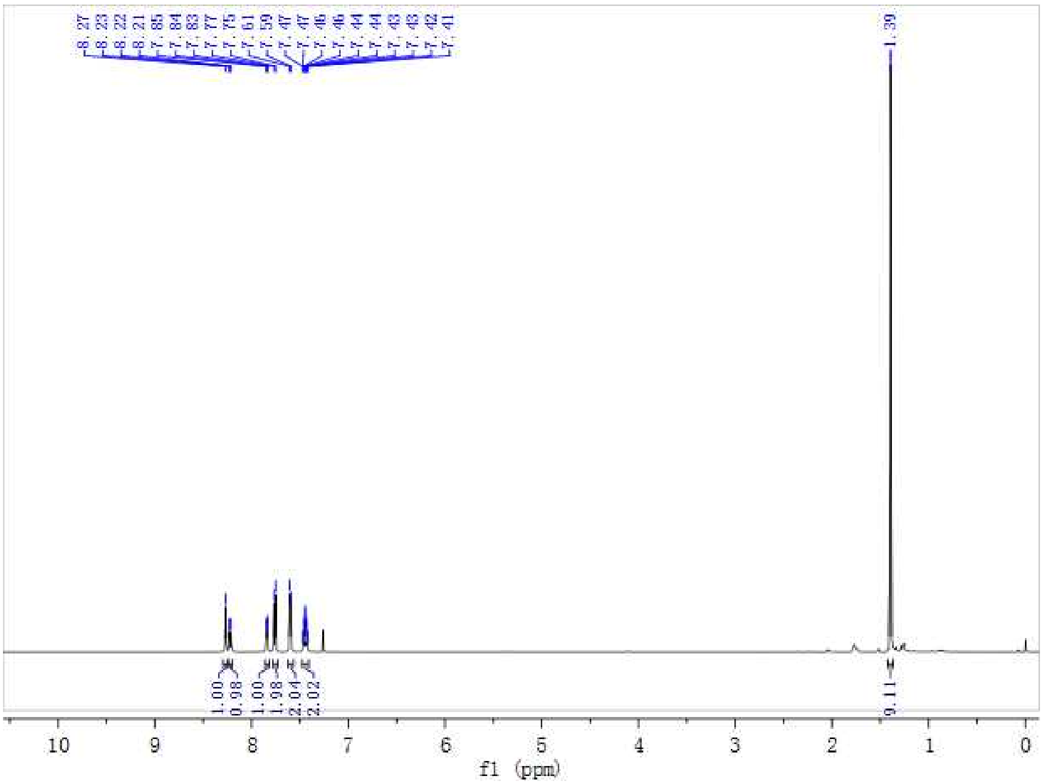

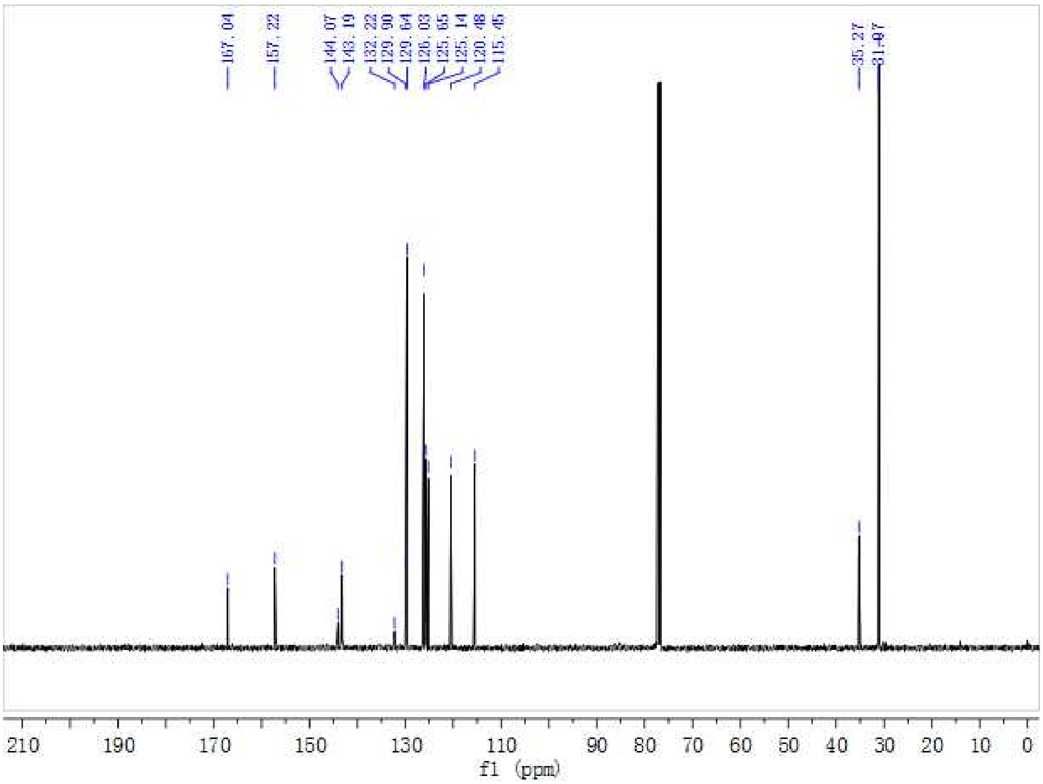

(1*H*-Benzo[*d*]imidazol-1-yl)(4-butylphenyl)methanone（new compound）

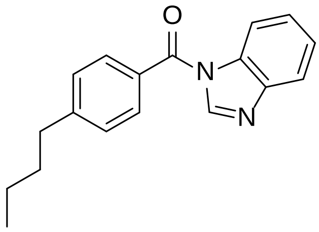

9

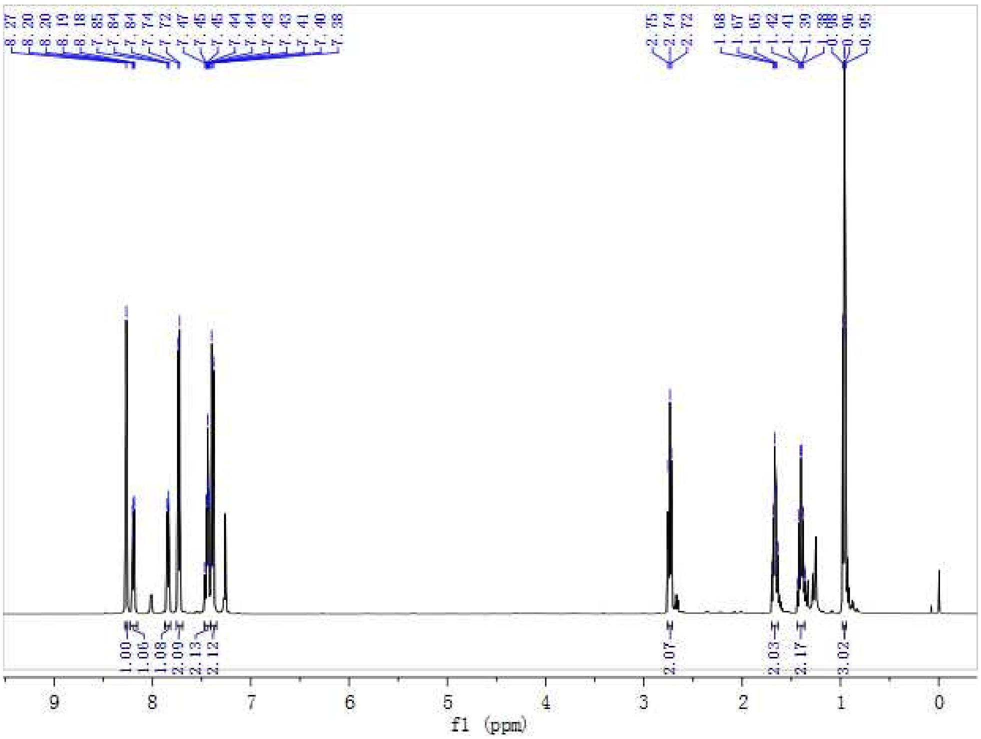

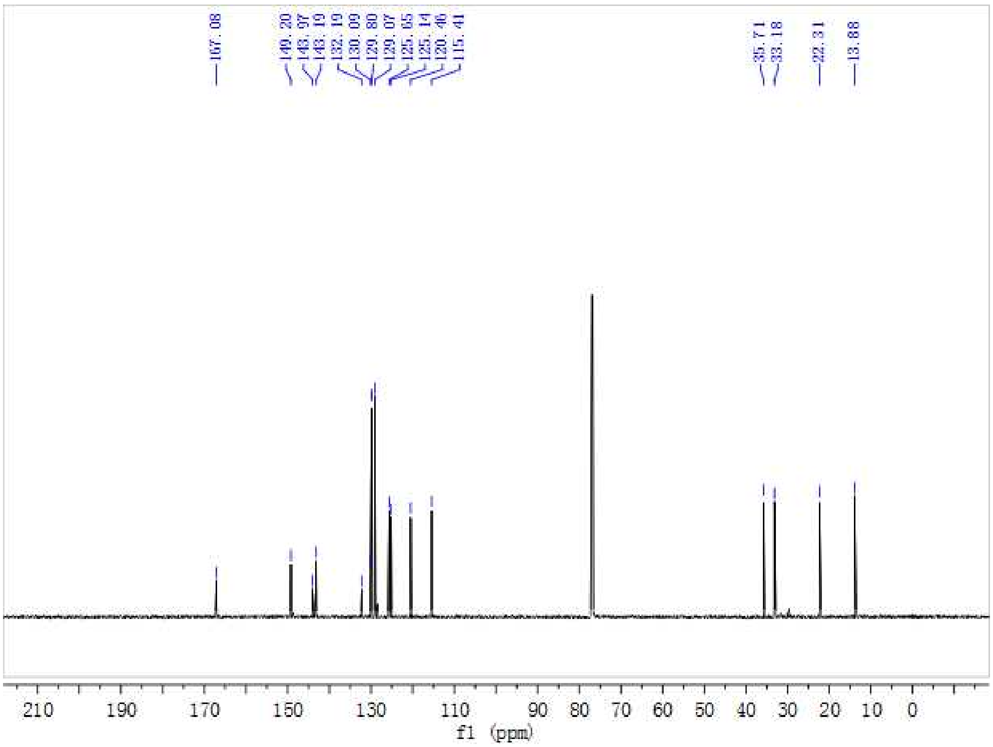

(1*H*-Benzo[*d*]imidazol-1-yl)(4-fluorophenyl)methanone（CAS：154786-24-0）

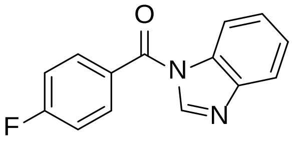

10

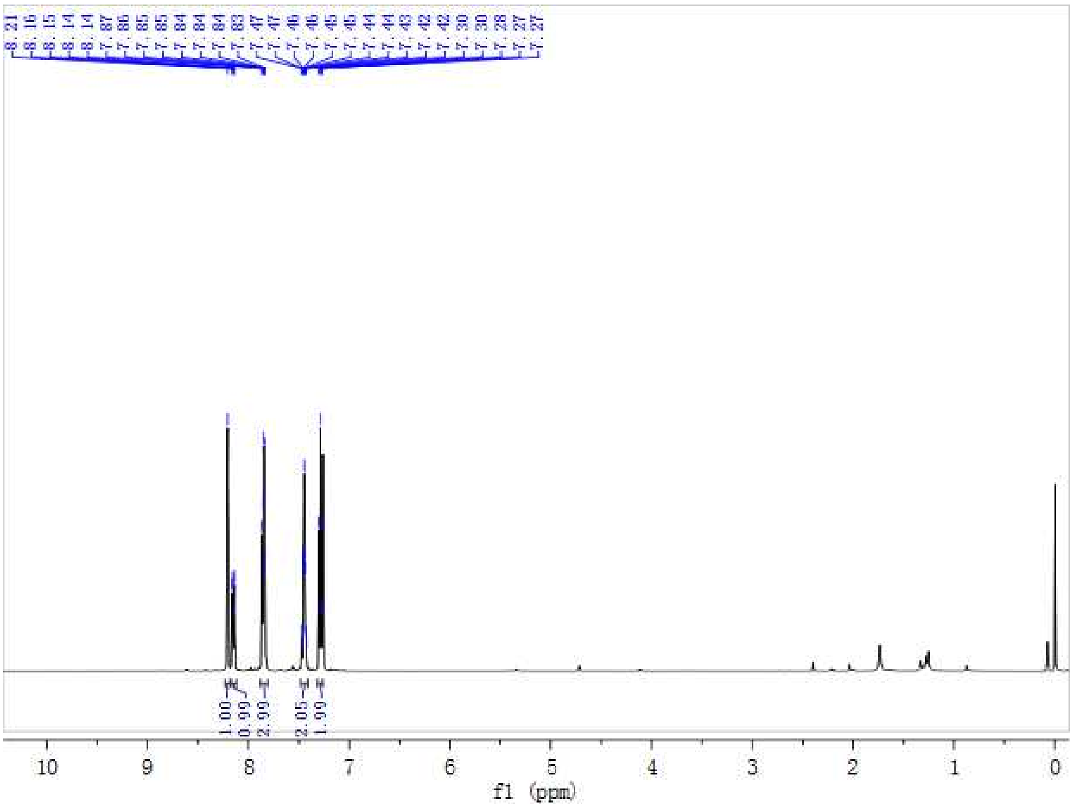

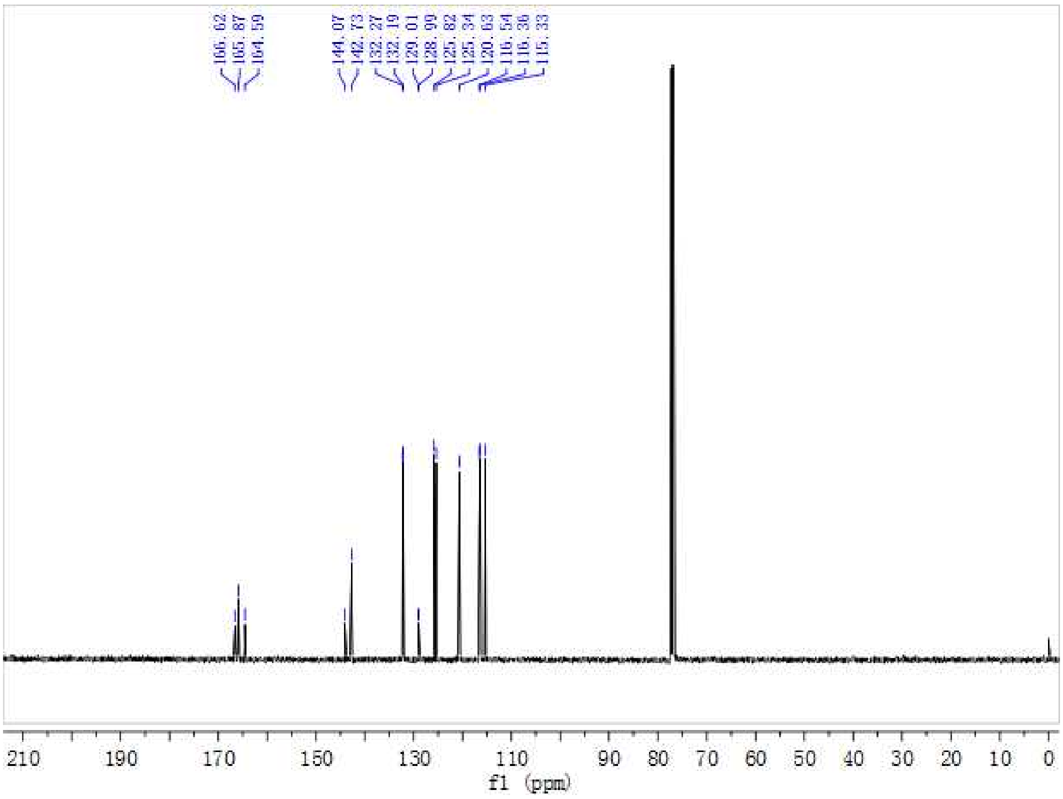

(1*H*-Benzo[*d*]imidazol-1-yl)(4-chlorophenyl)methanone（CAS：71589-37-2）

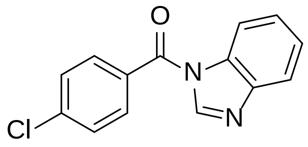

11

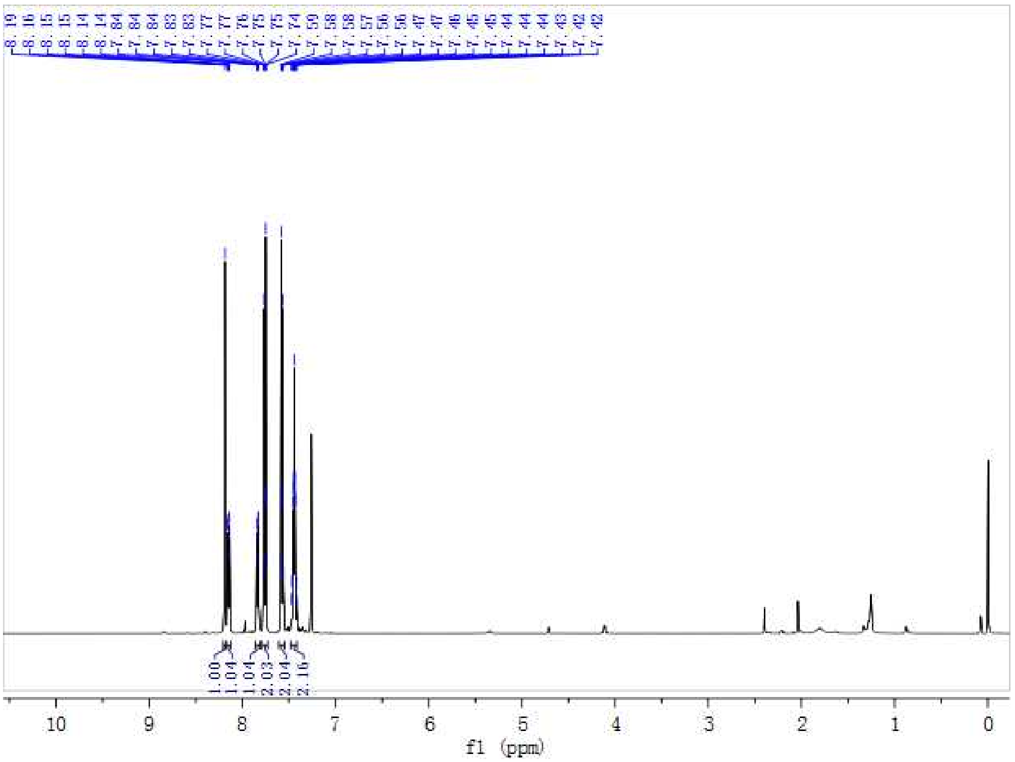

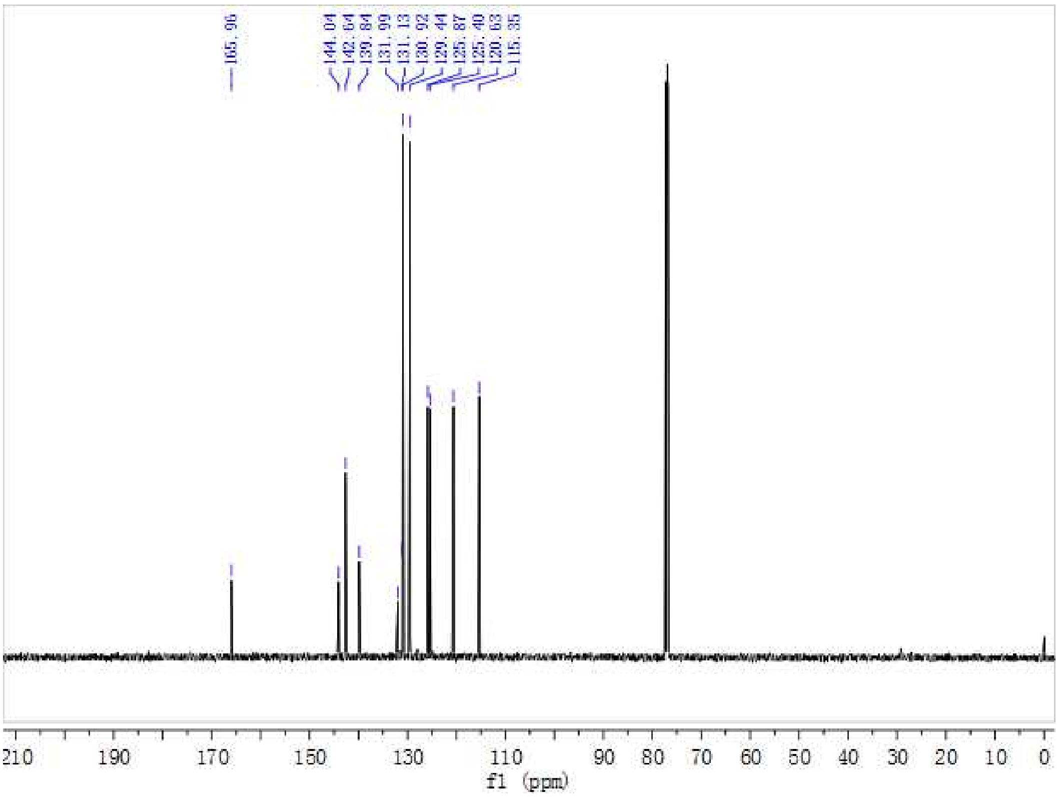

(1*H*-Benzo[*d*]imidazol-1-yl)(4-bromophenyl)methanone（CAS：304668-33-5）

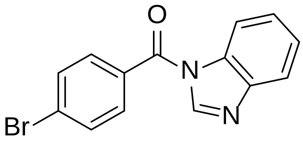

12

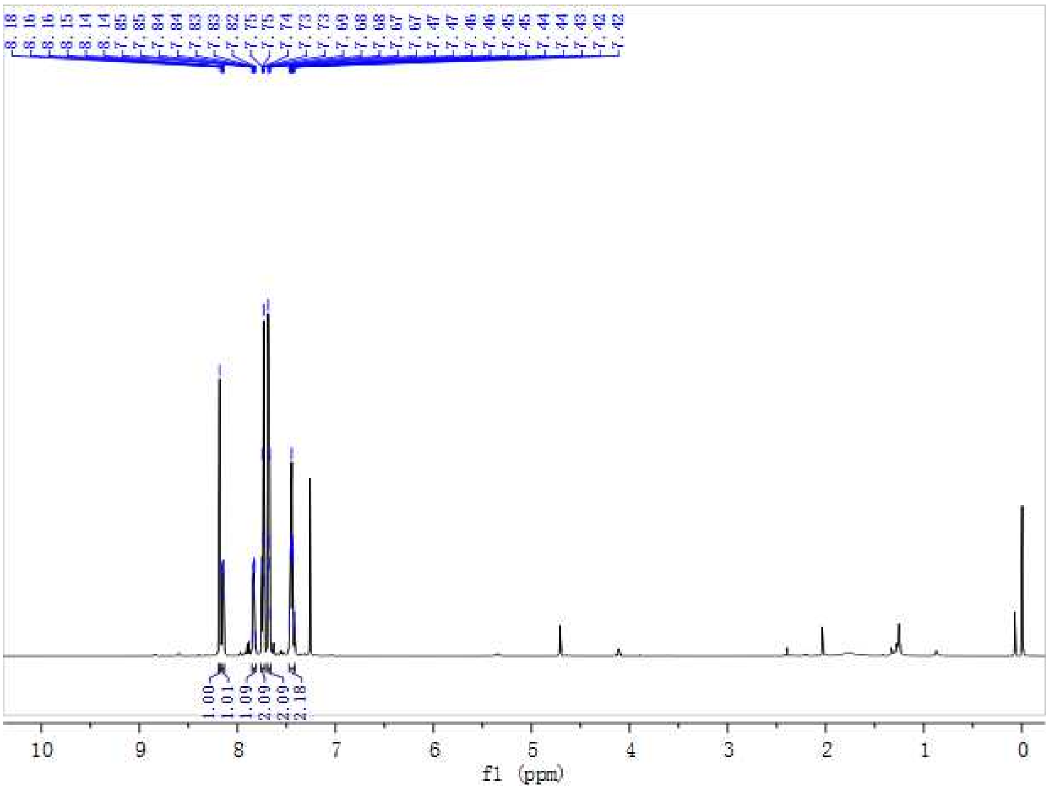

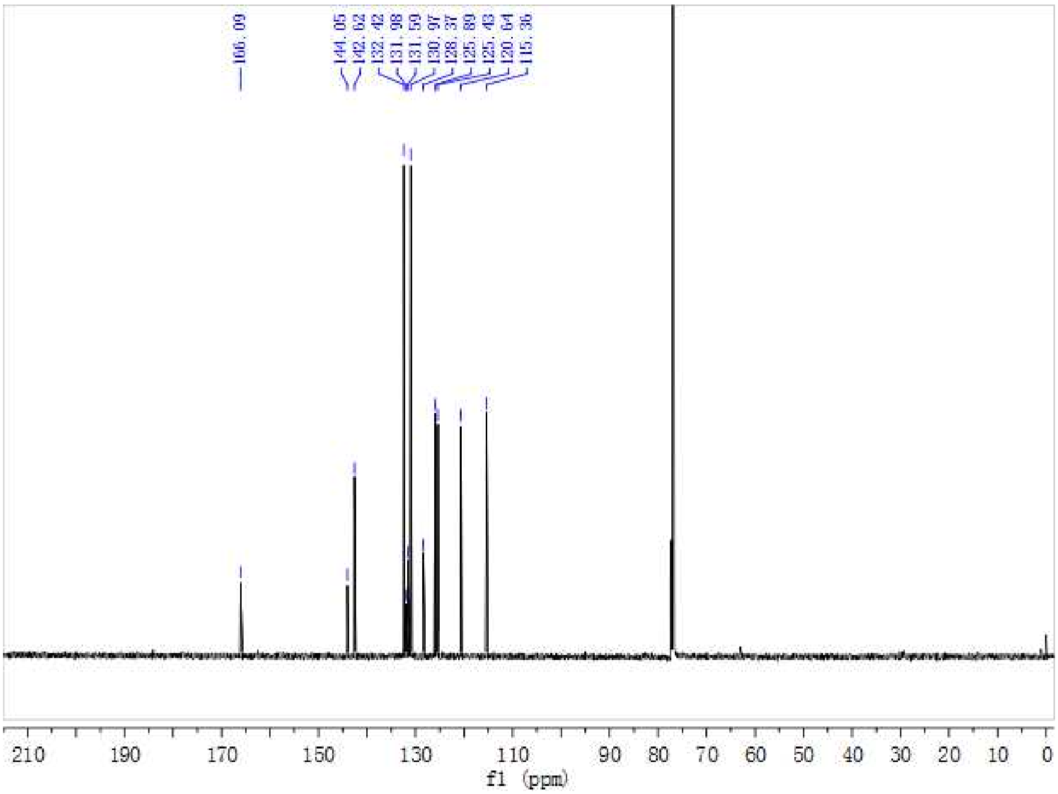

(1*H*-Benzo[*d*]imidazol-1-yl)(4-methoxyphenyl)methanone (CAS: 13361-55-2)

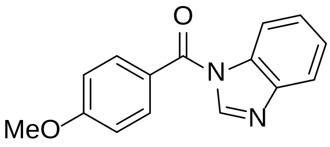

13

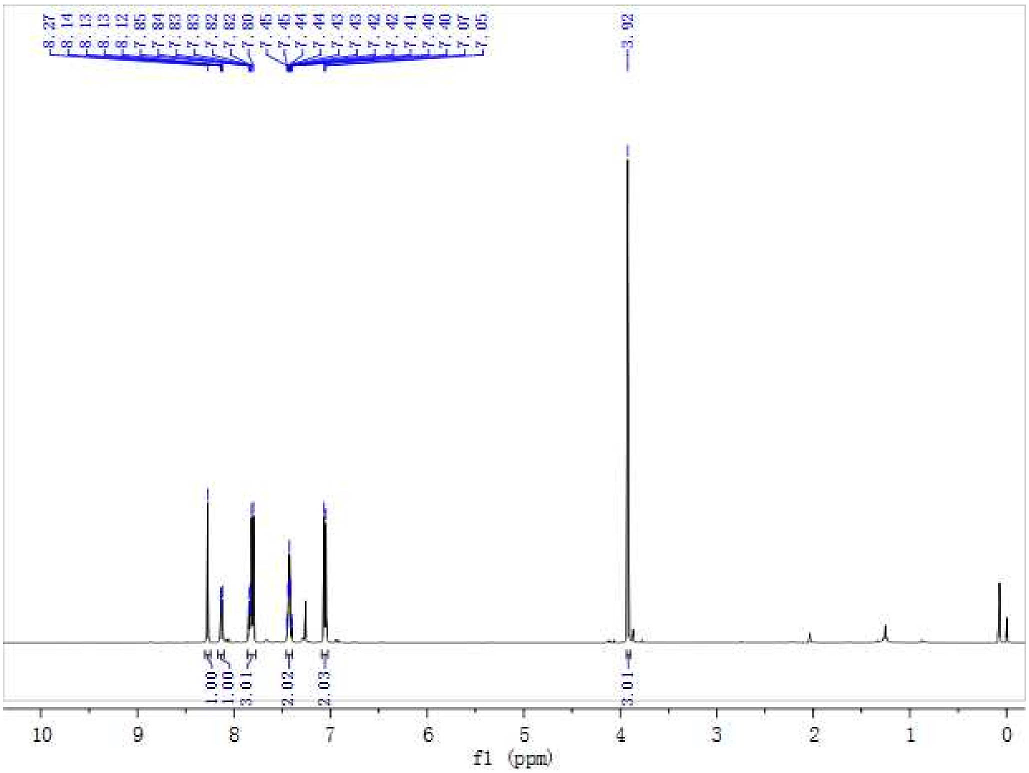

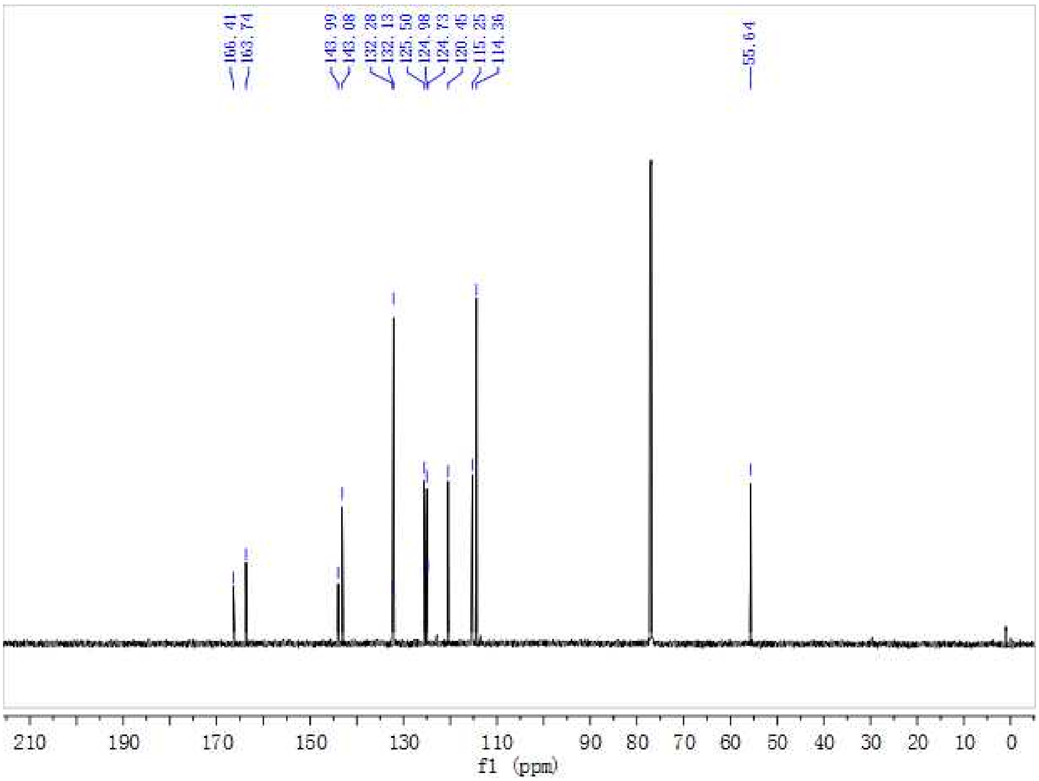

(1*H*-Benzo[*d*]imidazol-1-yl)(4-vinylphenyl)methanone (CAS: 300396-82-1)

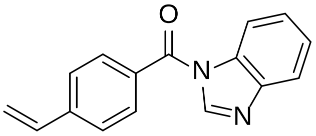

14

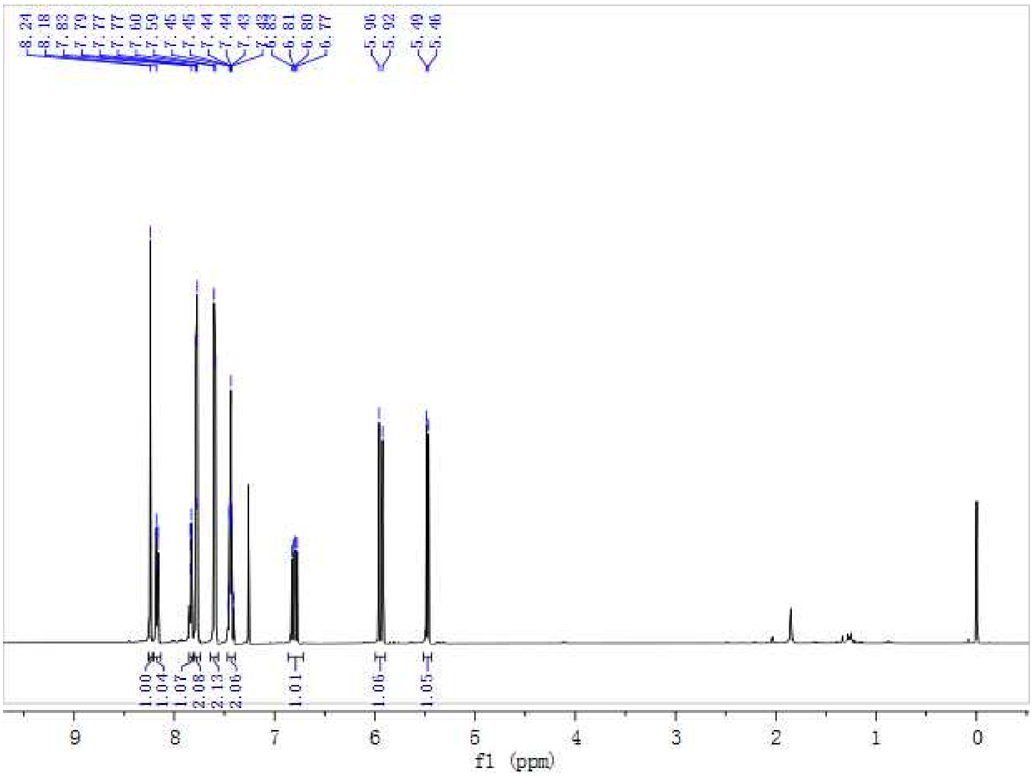

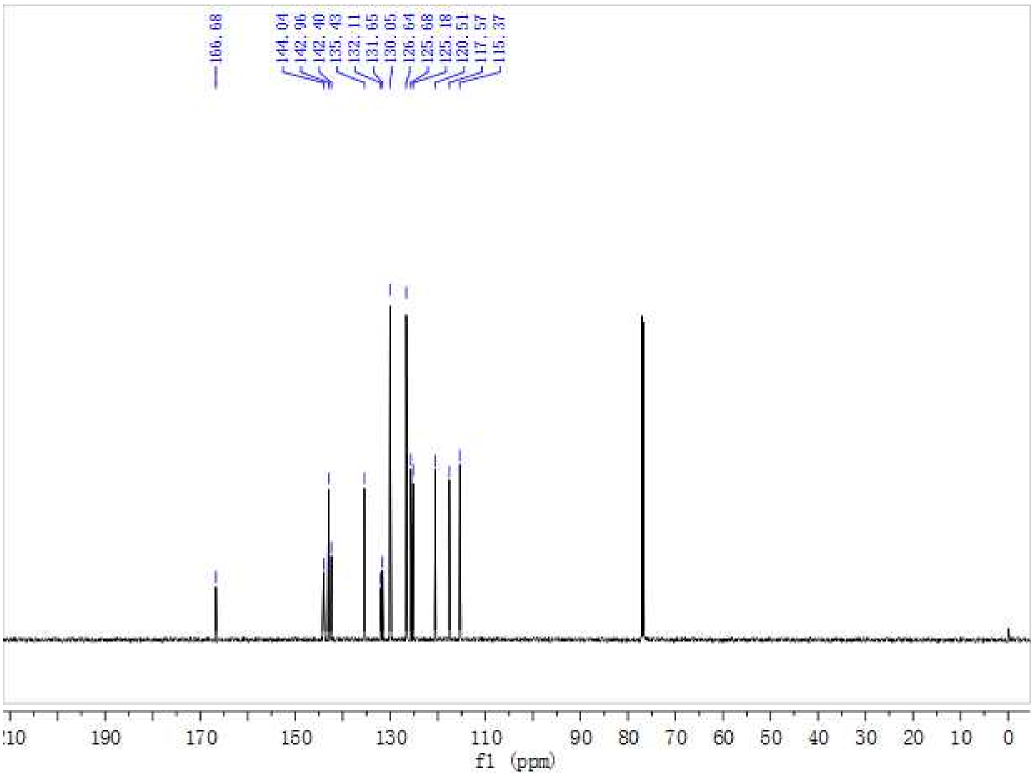

(1*H*-Benzo[*d*]imidazol-1-yl)(4-cyclohexylphenyl)methanone (new compound)

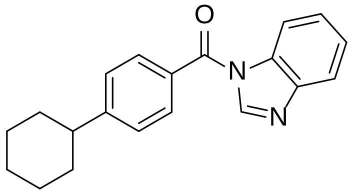

15

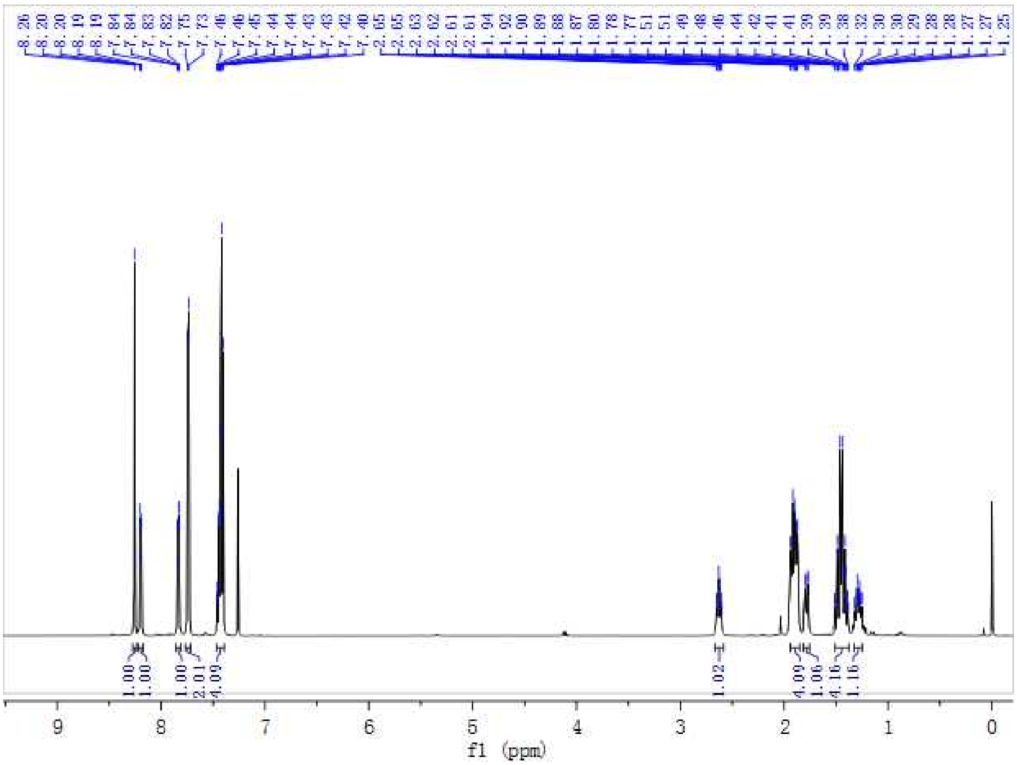

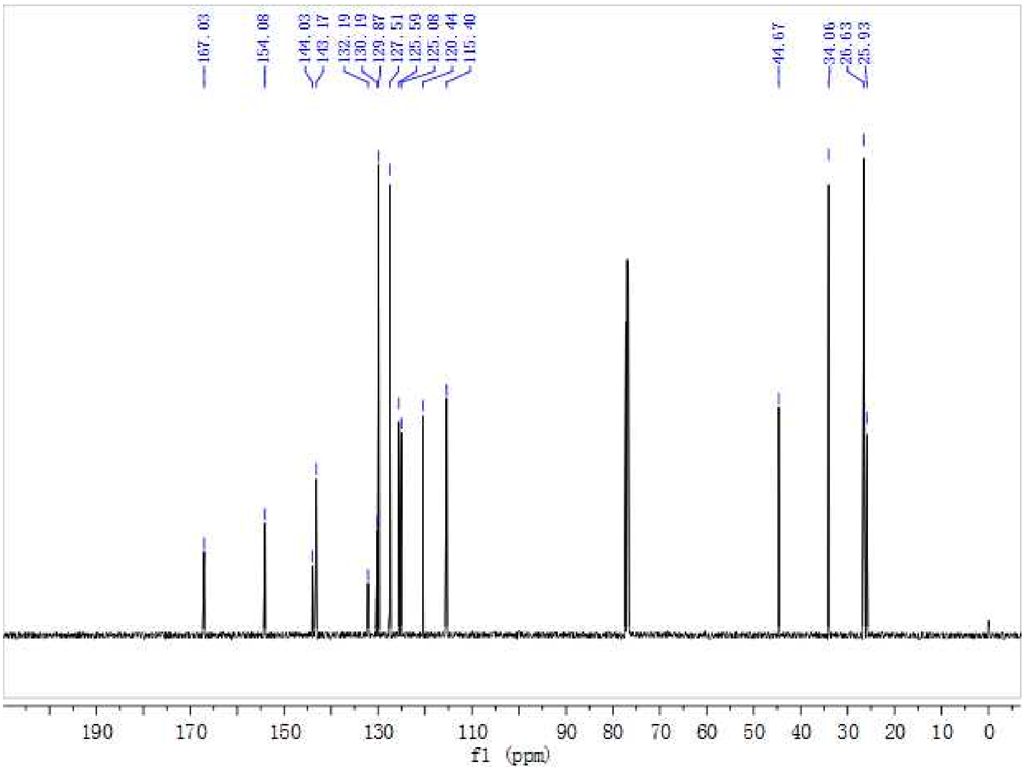

(1*H*-Benzo[*d*]imidazol-1-yl)(*o*-tolyl)methanone (CAS: 200626-52-4)

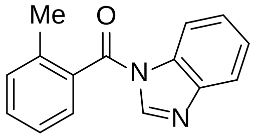

16

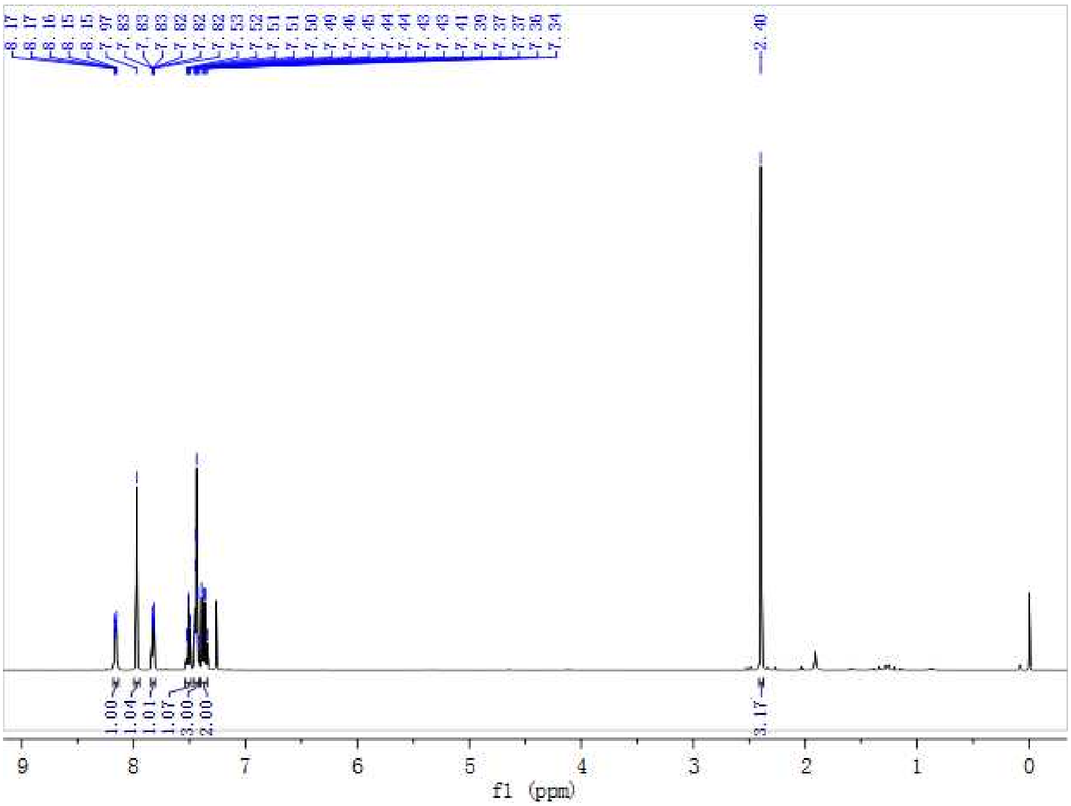

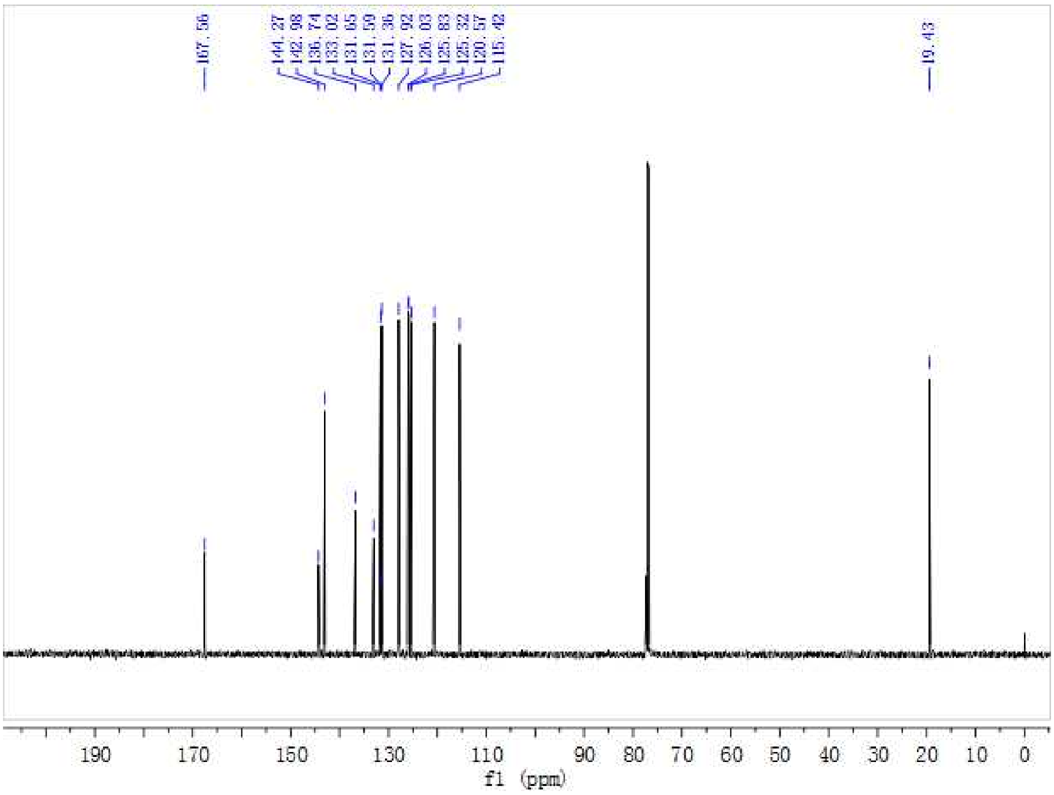

(1*H*-Benzo[*d*]imidazol-1-yl)(3,4-dimethylphenyl)methanone (CAS: 333348-55-3)

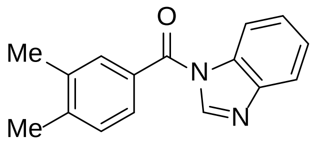

17

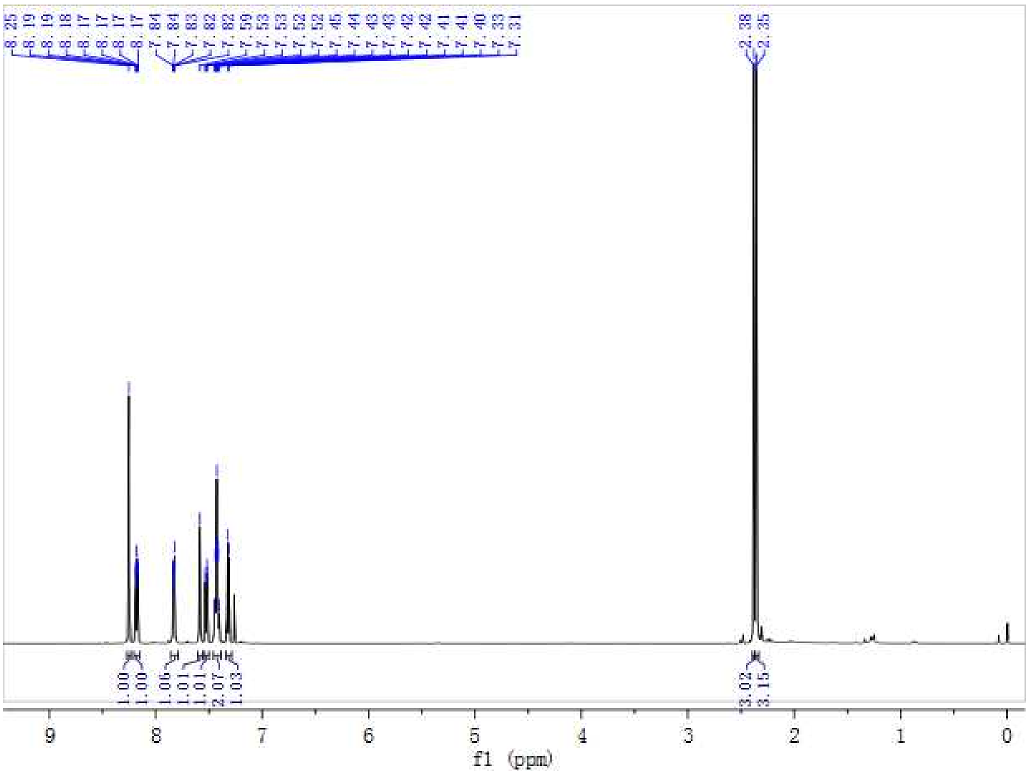

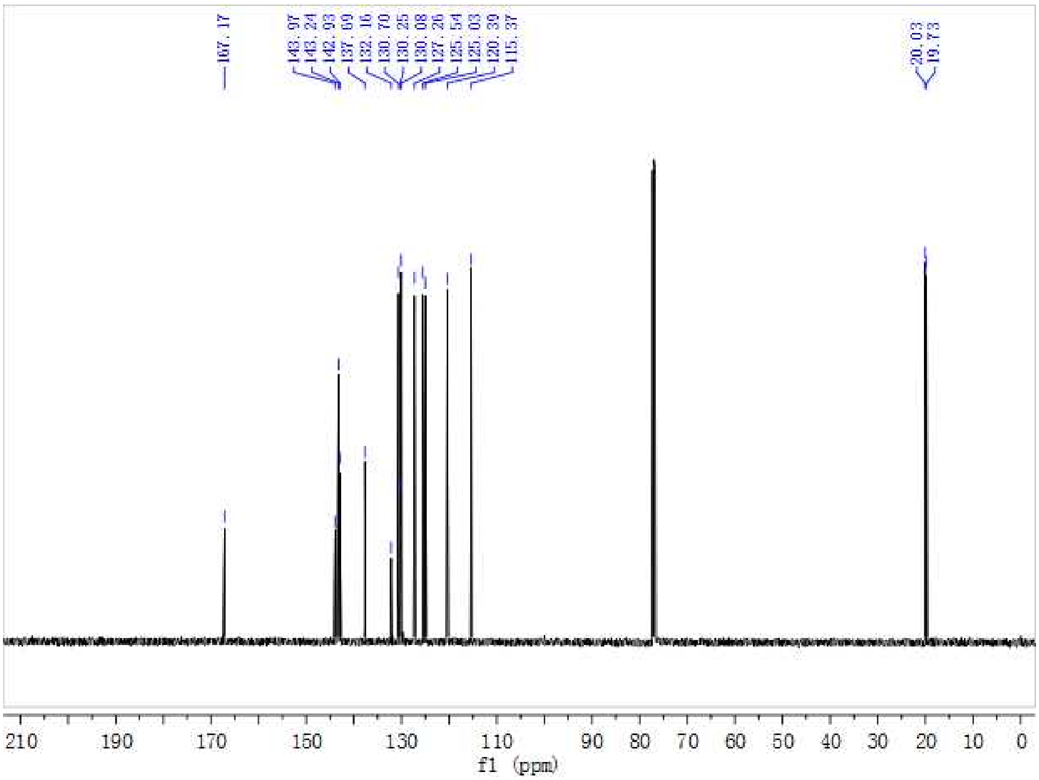

(1*H*-Benzo[*d*]imidazol-1-yl)(3,4-dichlorophenyl)methanone (CAS: 330215-63-9)

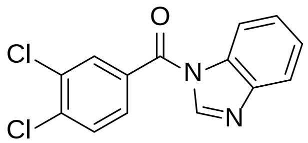

18

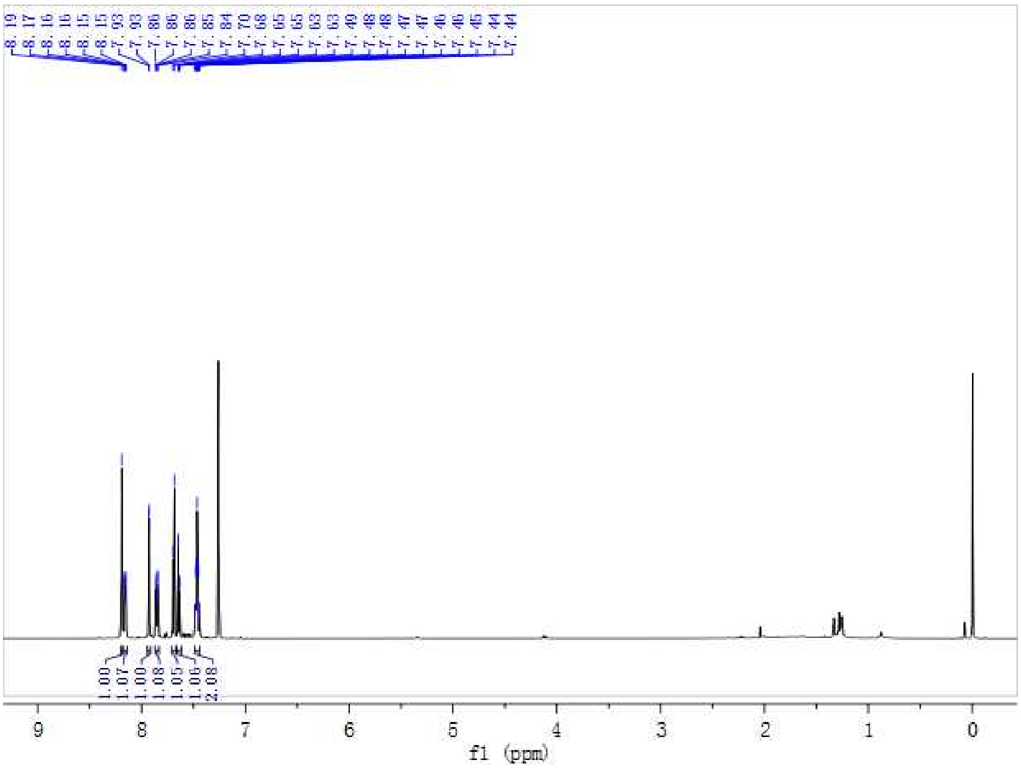

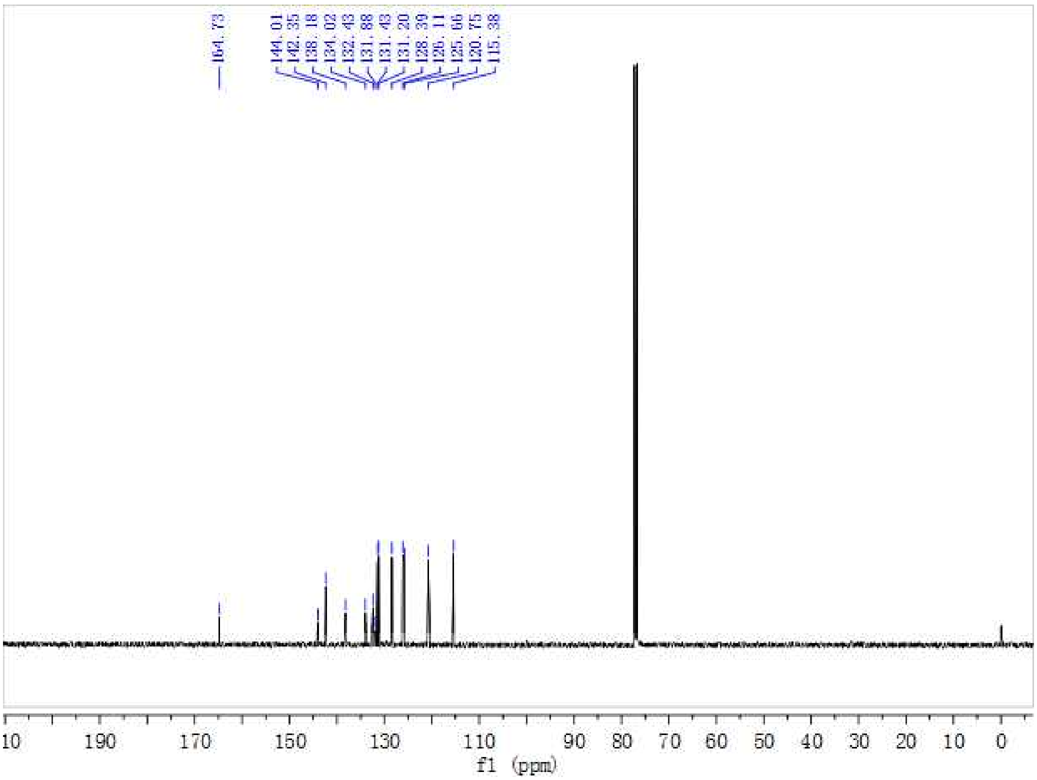

(1*H*-Benzo[*d*]imidazol-1-yl)(3-chlorophenyl)methanone (CAS :200626-53-5)

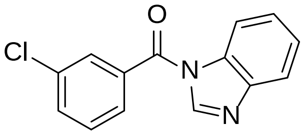

19

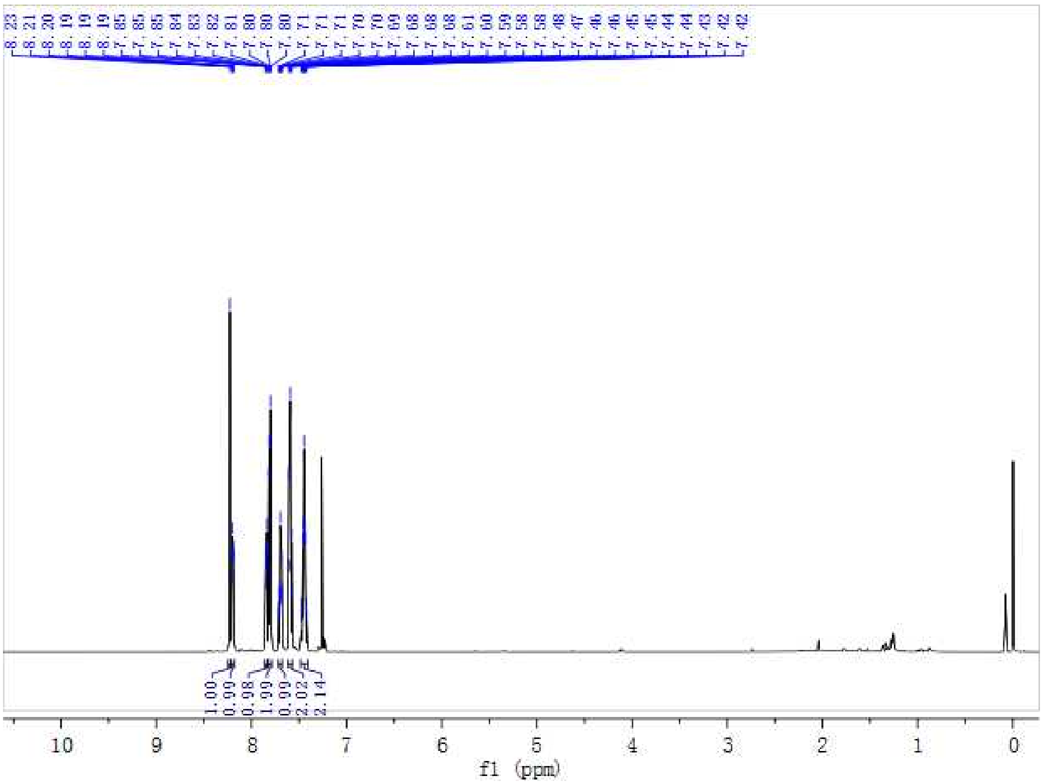

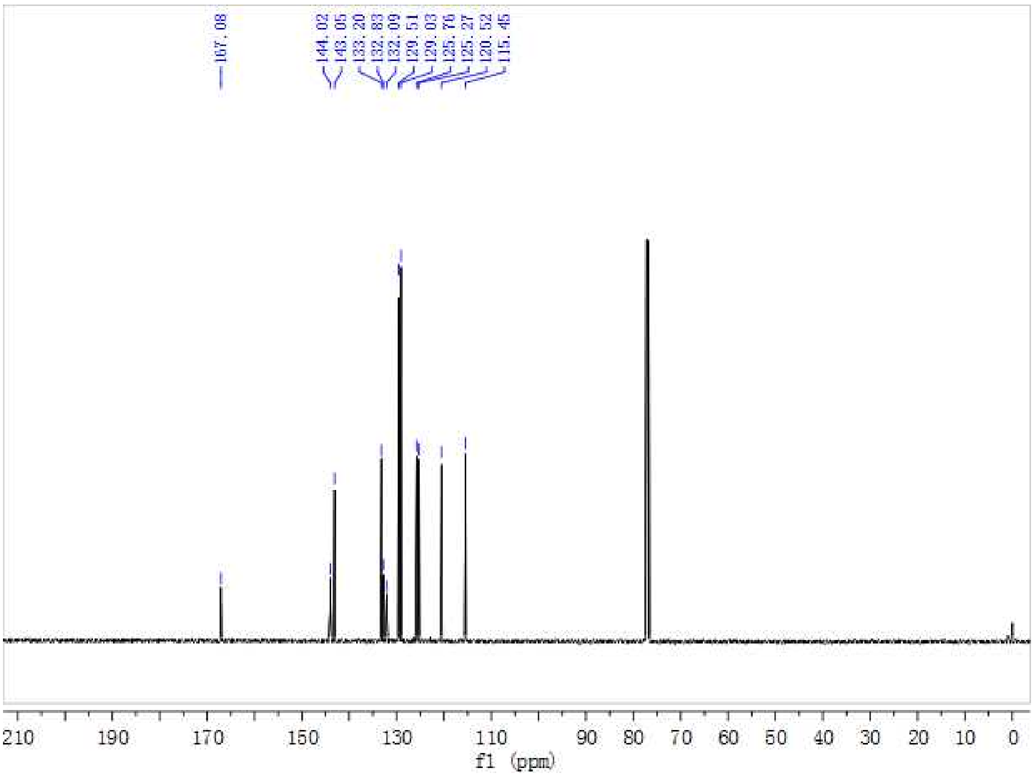

(1*H*-Benzo[*d*]imidazol-1-yl)(naphthalen-2-yl)methanone（new compound）

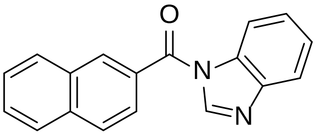

20

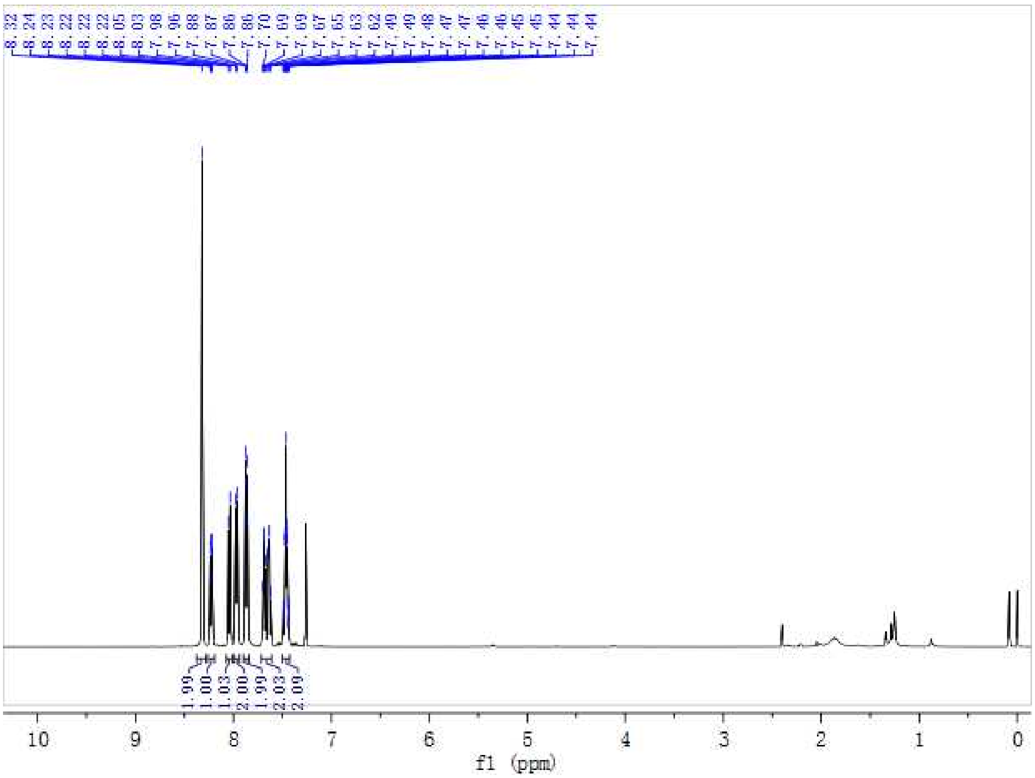

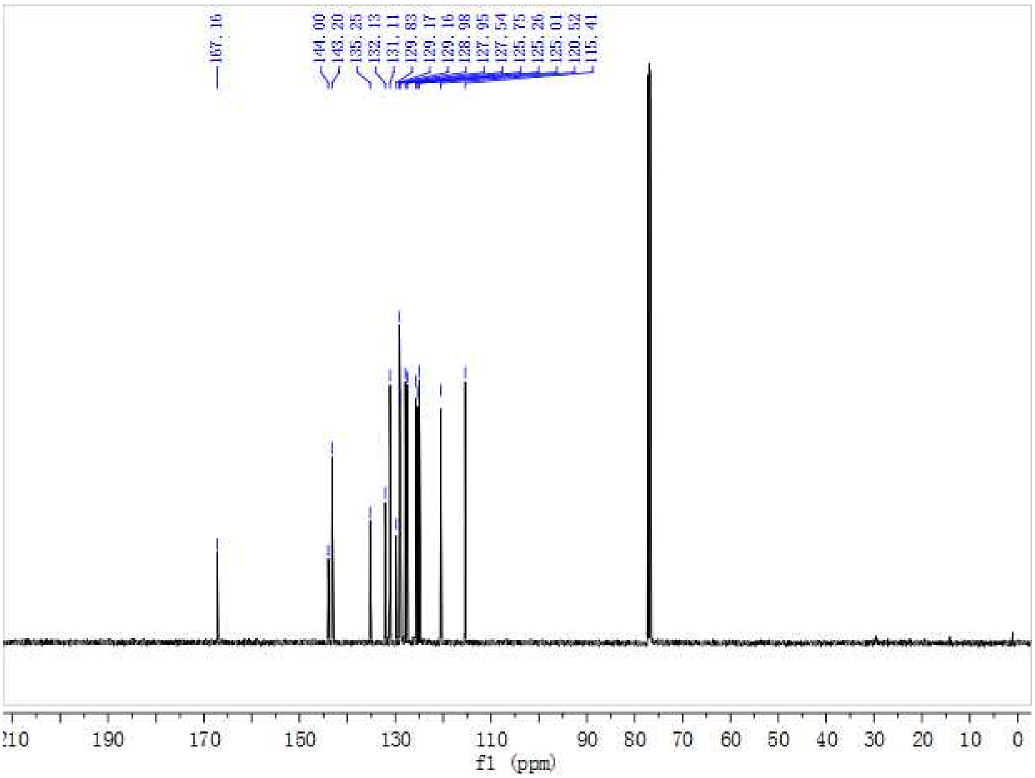

(1*H*-Benzo[*d*]imidazol-1-yl)(pyren-4-yl)methanone (new compound)

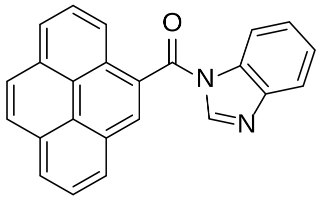

21

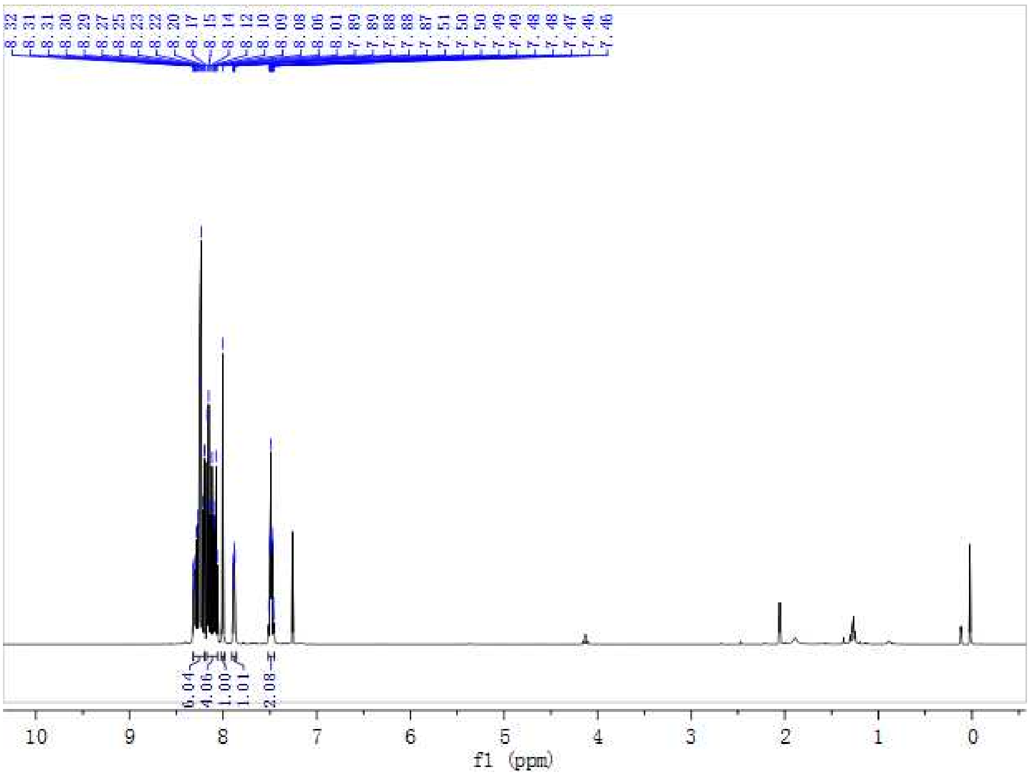

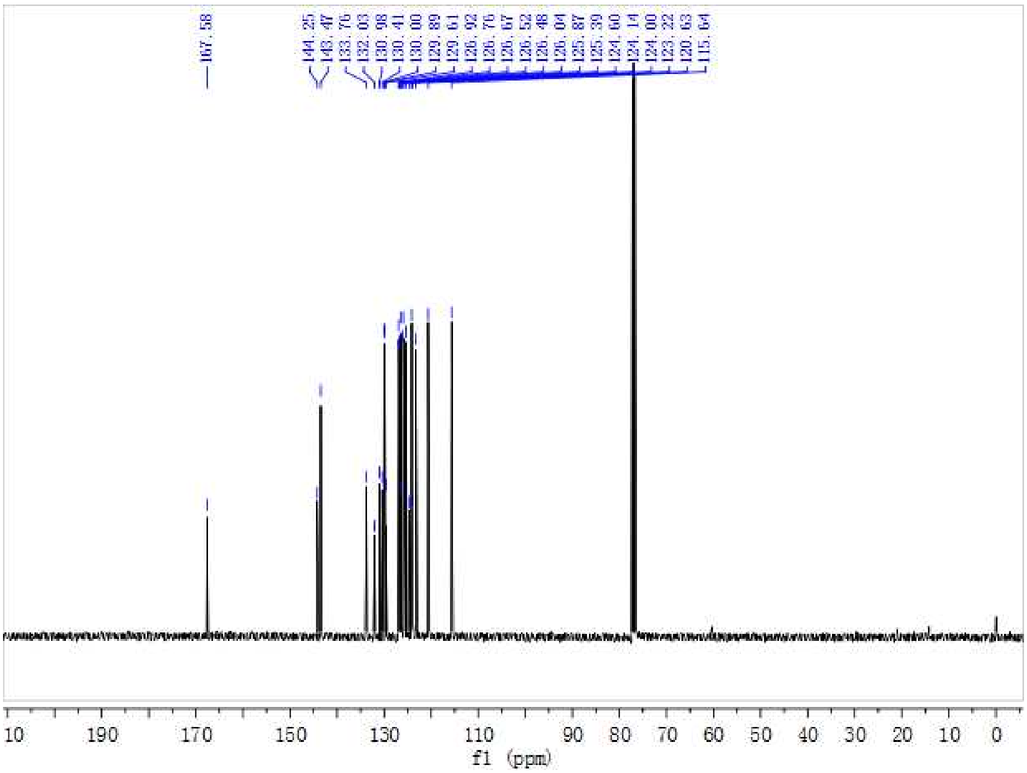

(1*H*-Benzo[*d*]imidazol-1-yl)(thiophen-3-yl)methanone（new compound）

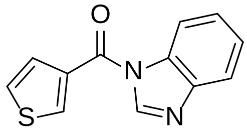

22

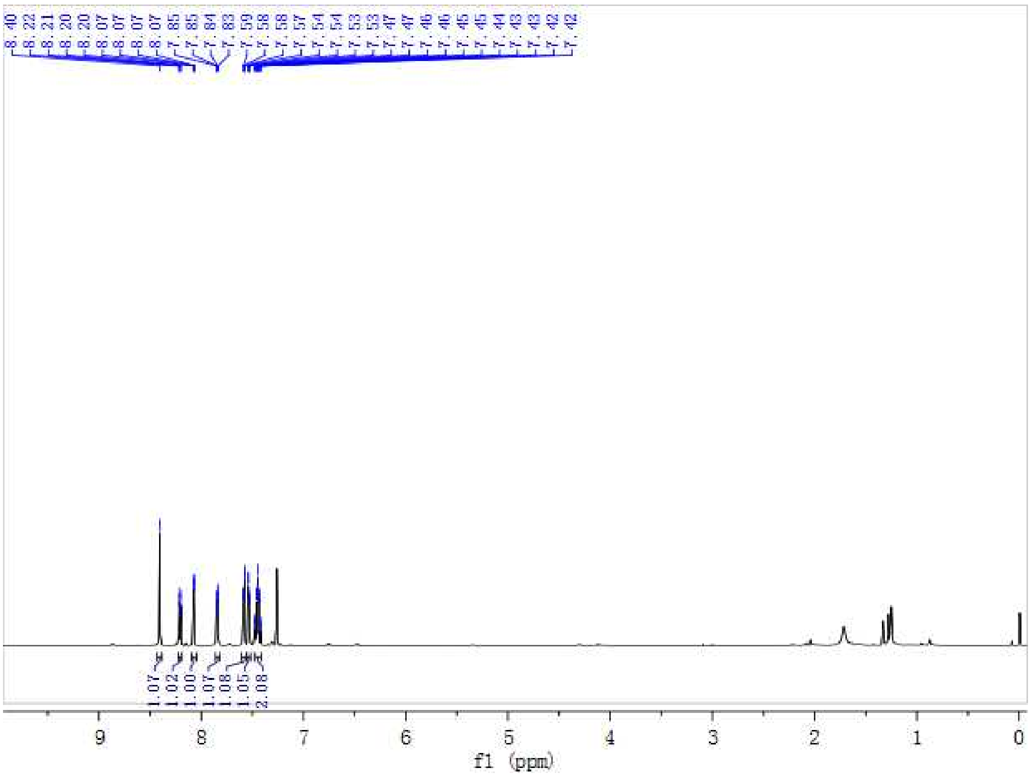

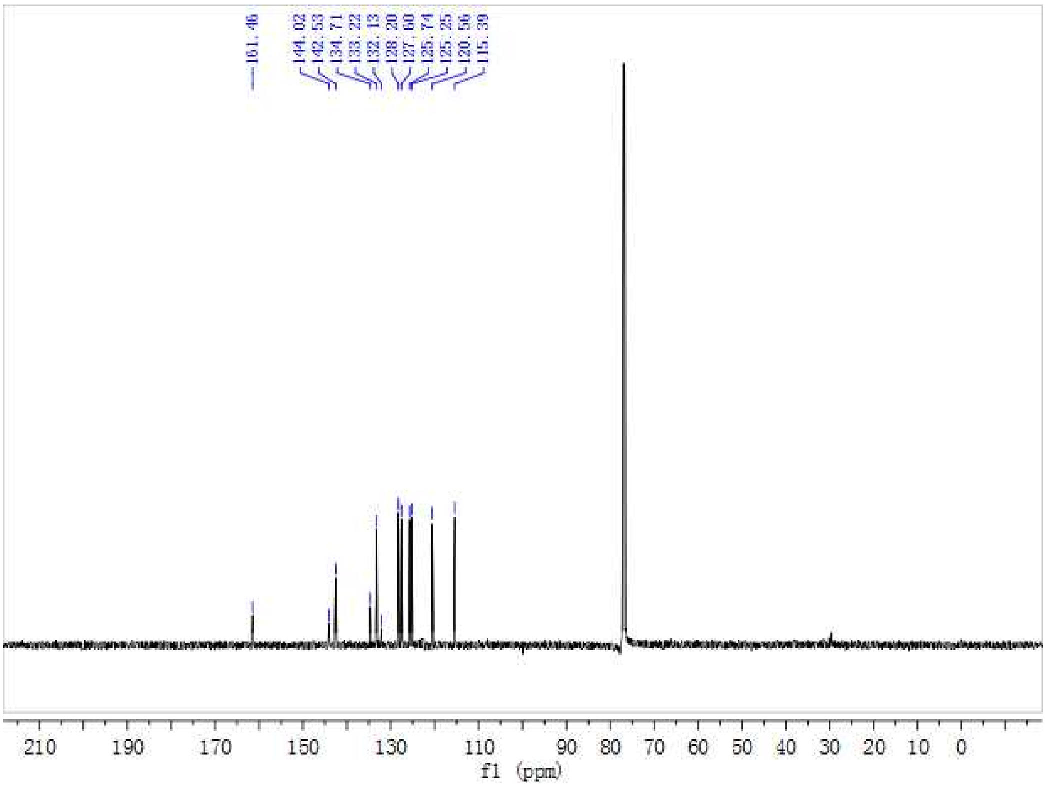

(1*H*-Benzo[*d*]imidazol-1-yl)(furan-3-yl)methanone (new compound)

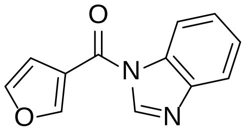

23

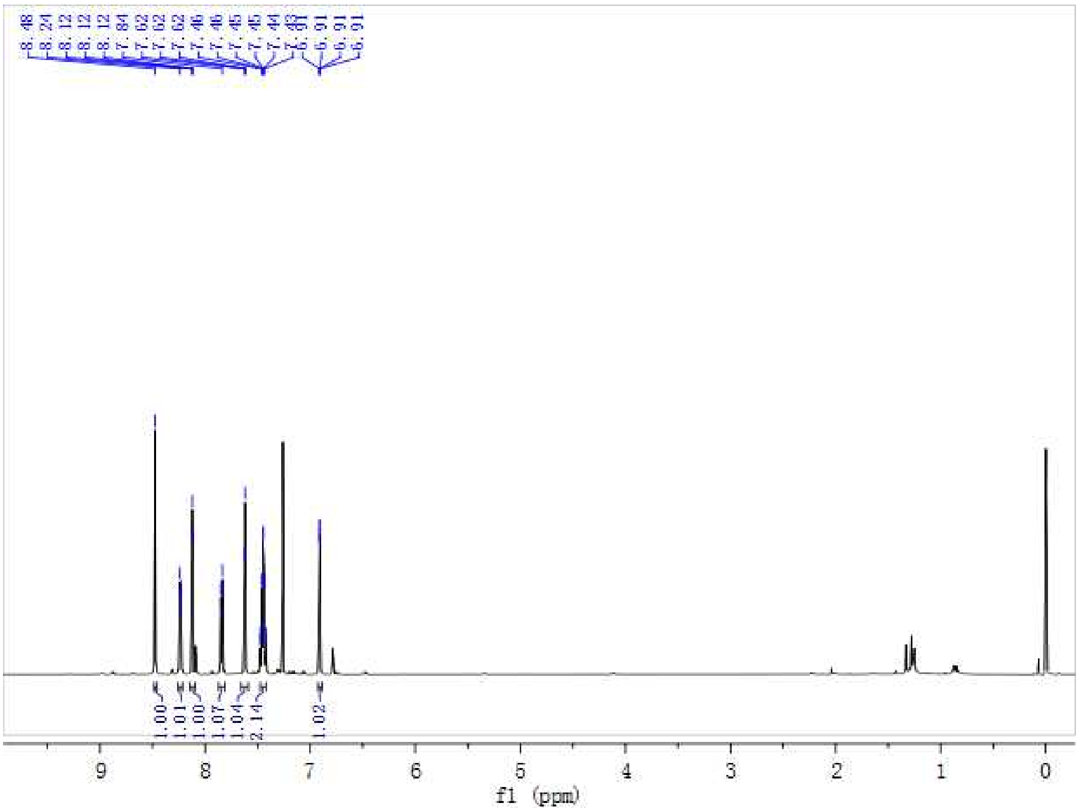

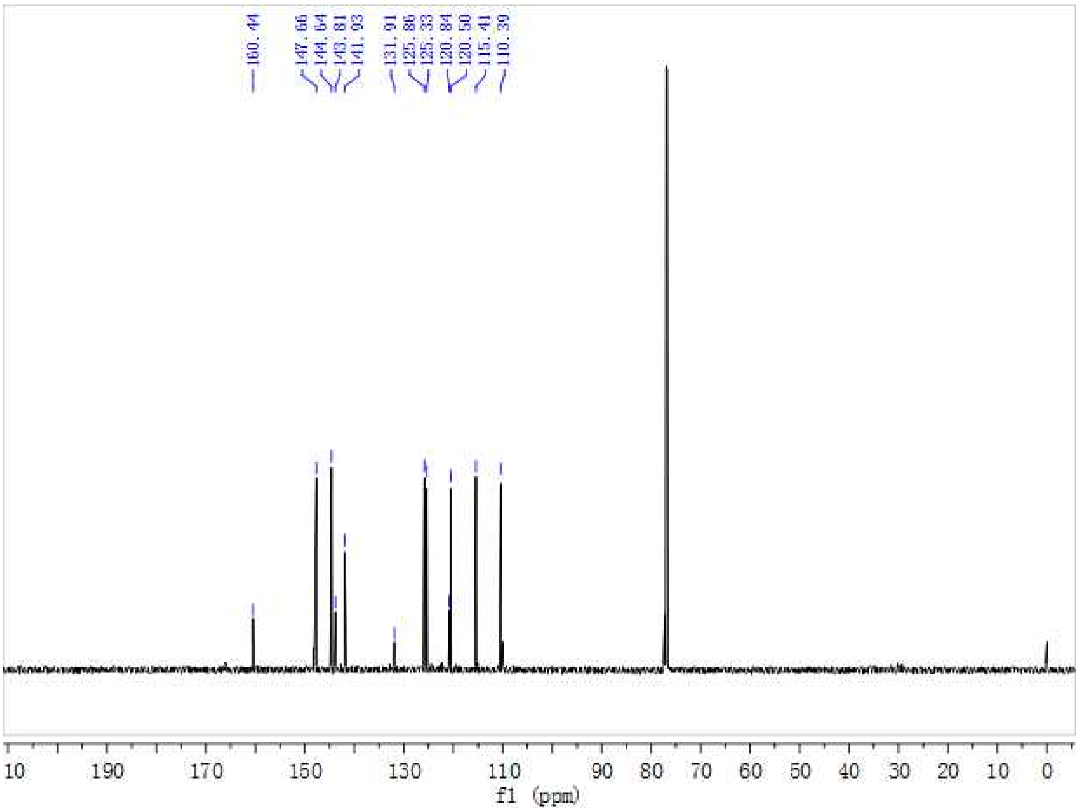

(1*H*-Benzo[d]imidazol-1-yl)(cyclohexyl)methanone (CAS: 294649-09-5)

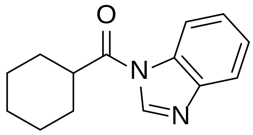

24

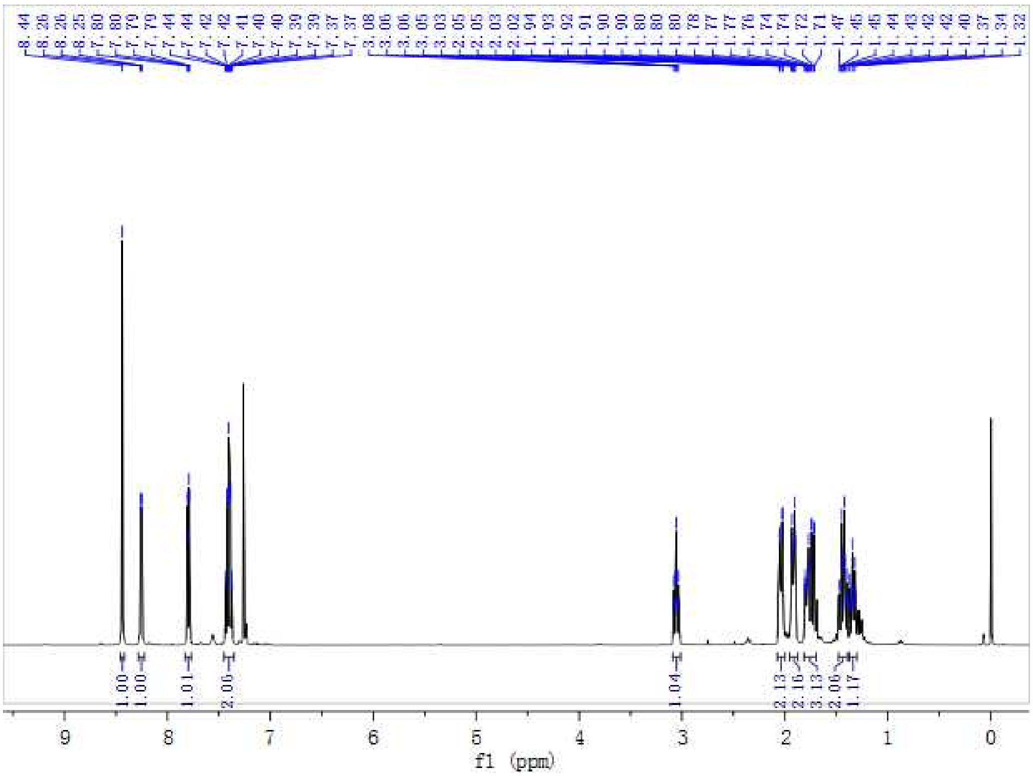

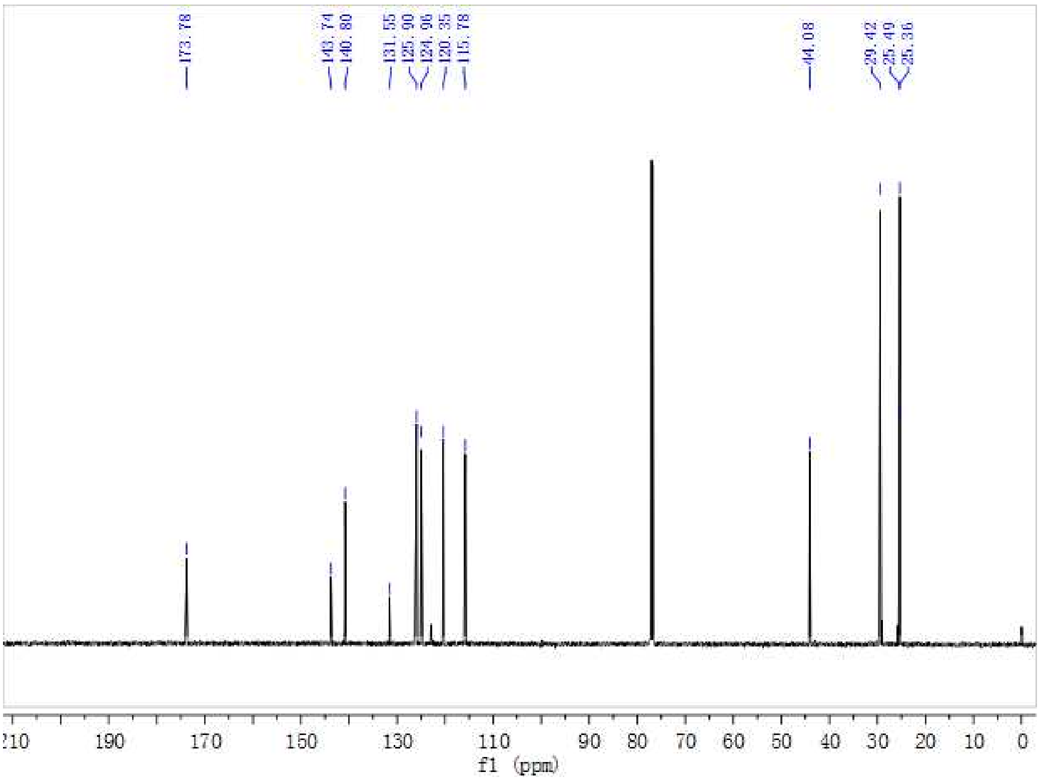

1-(1*H*-Benzo[*d*]imidazol-1-yl)hexan-1-one (CAS: 901547-84-0)

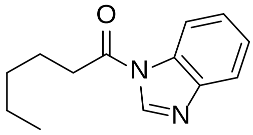

25

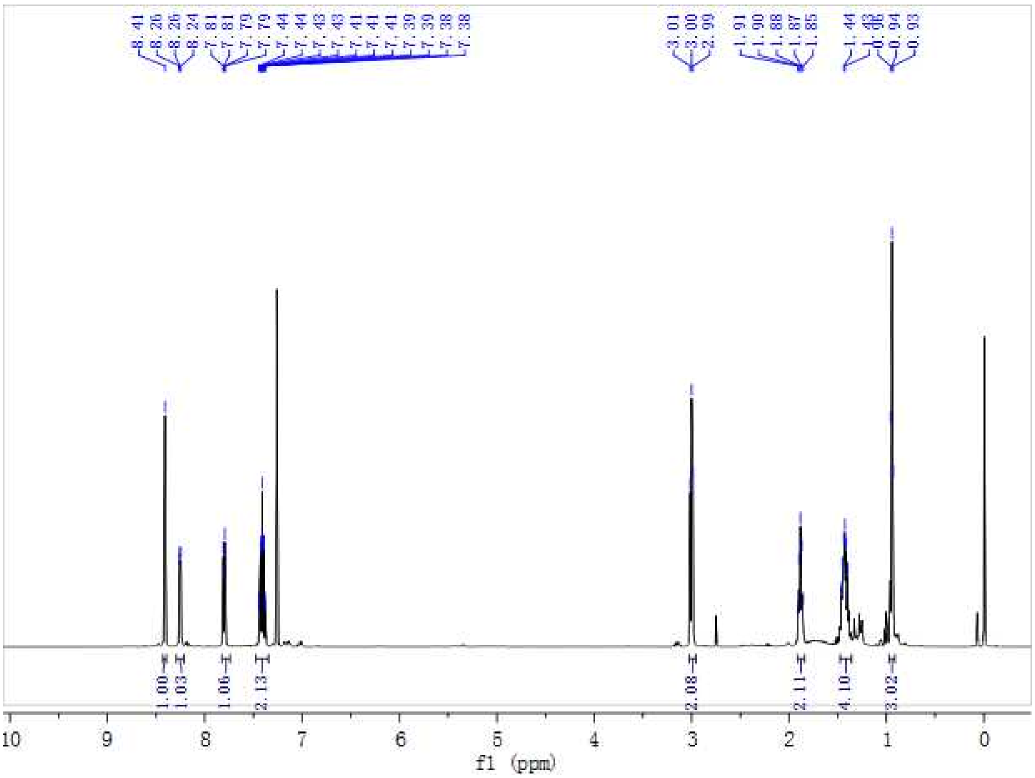

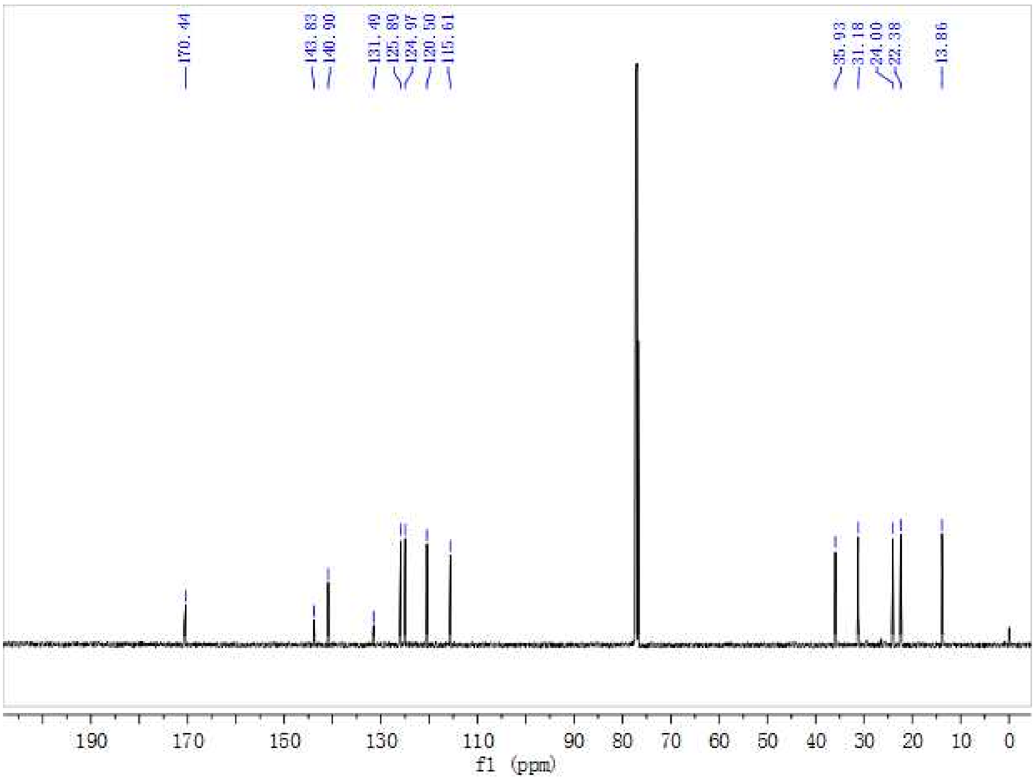

1-(1*H*-Benzo[*d*]imidazol-1-yl)ethan-1-one (CAS: 18773-95-0)

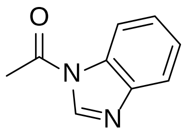

26

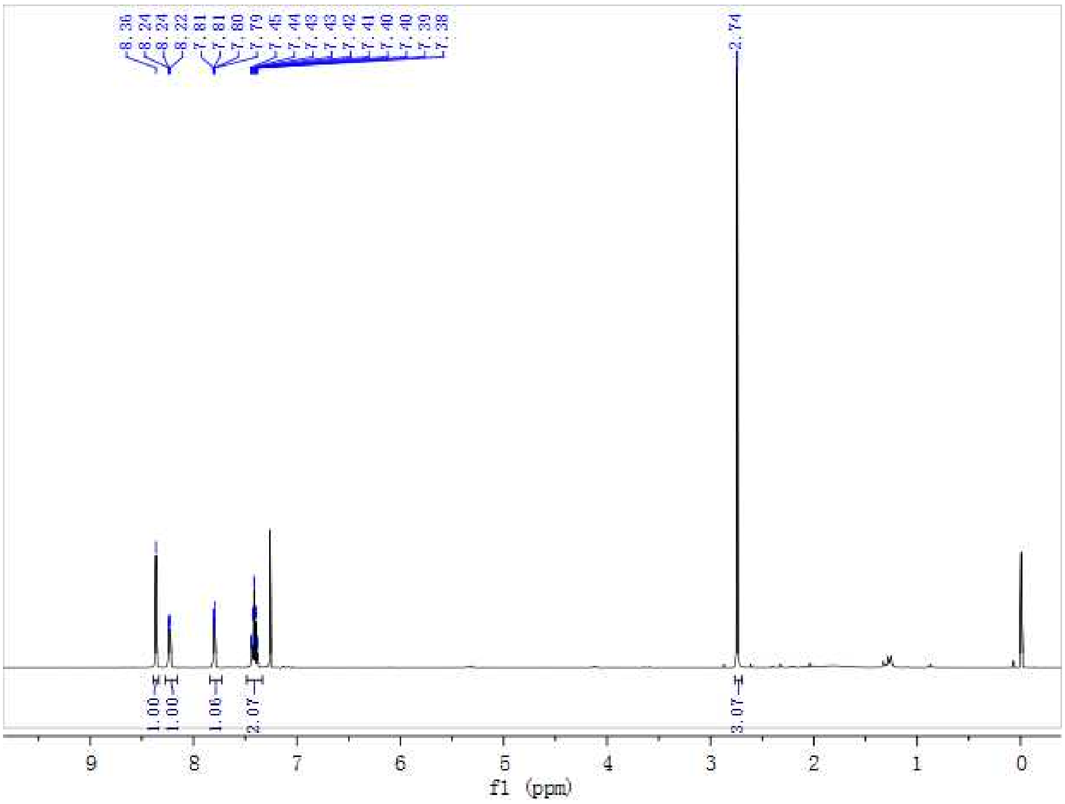

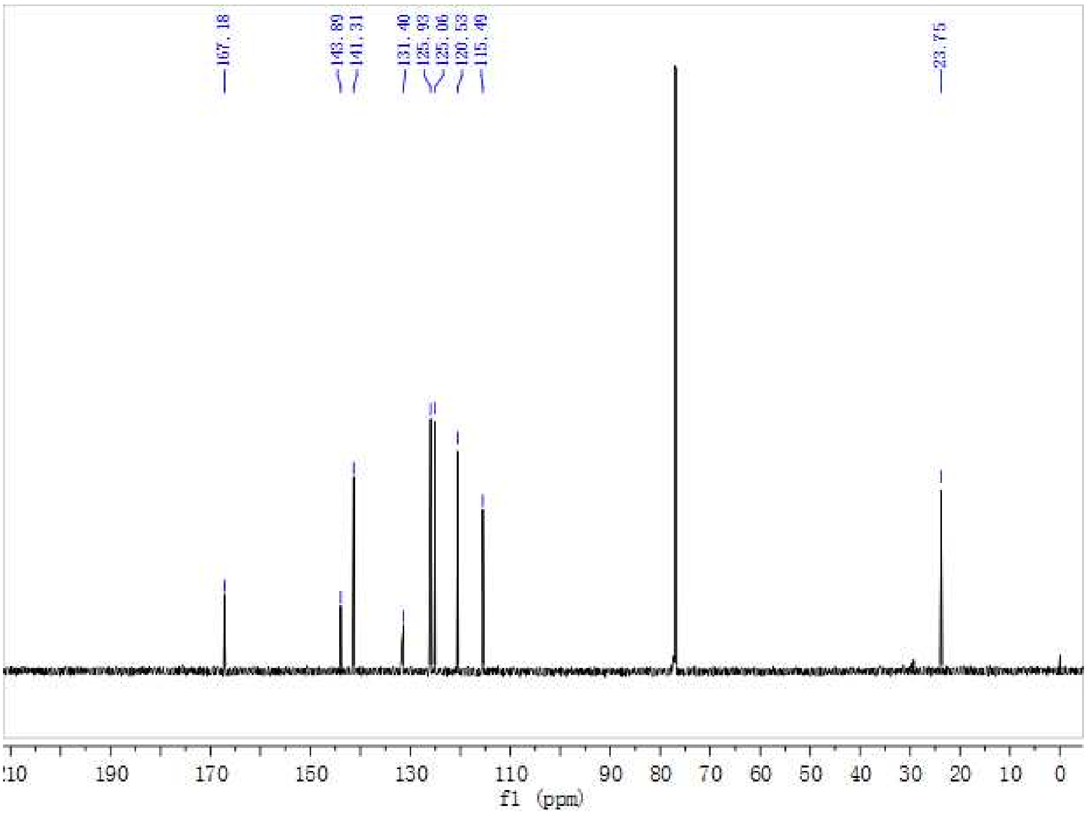

(5,6-Dimethyl-1*H*-benzo[*d*]imidazol-1-yl)(phenyl)methanone（CAS：16109-46-9）

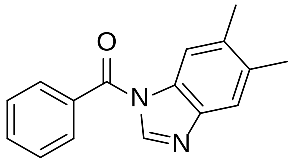

27

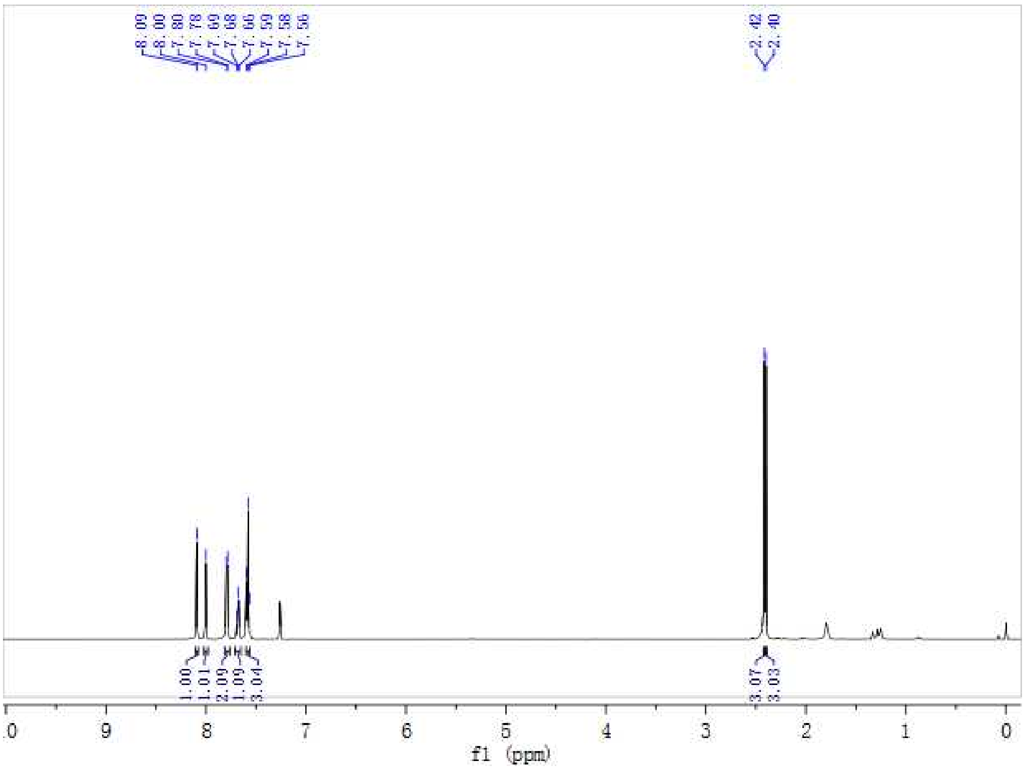

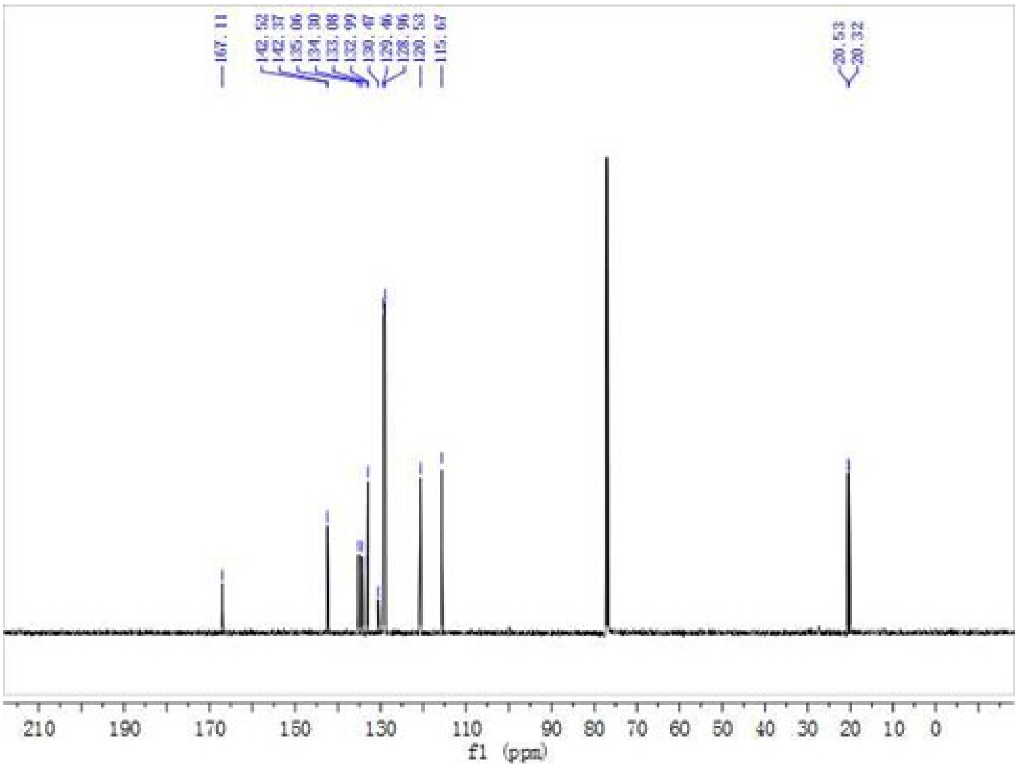

***N*-(Pyridin-2-yl)benzamide (CAS : 4589-12-2)**

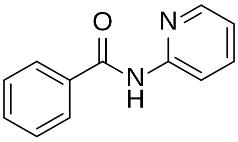

29

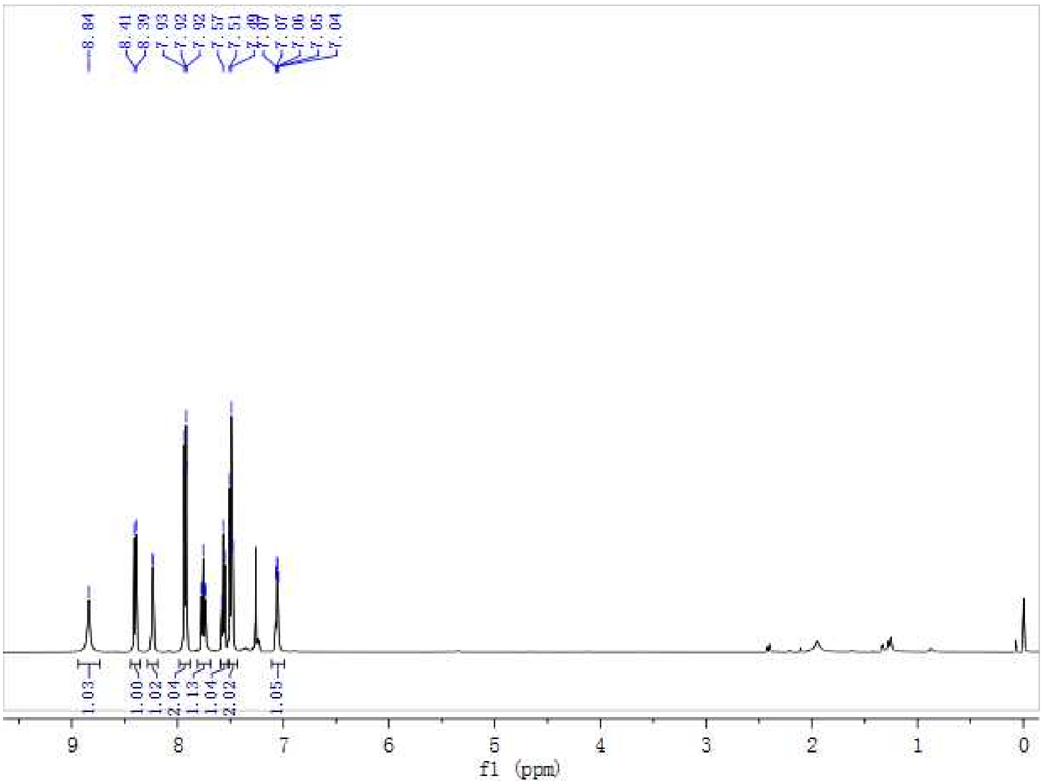

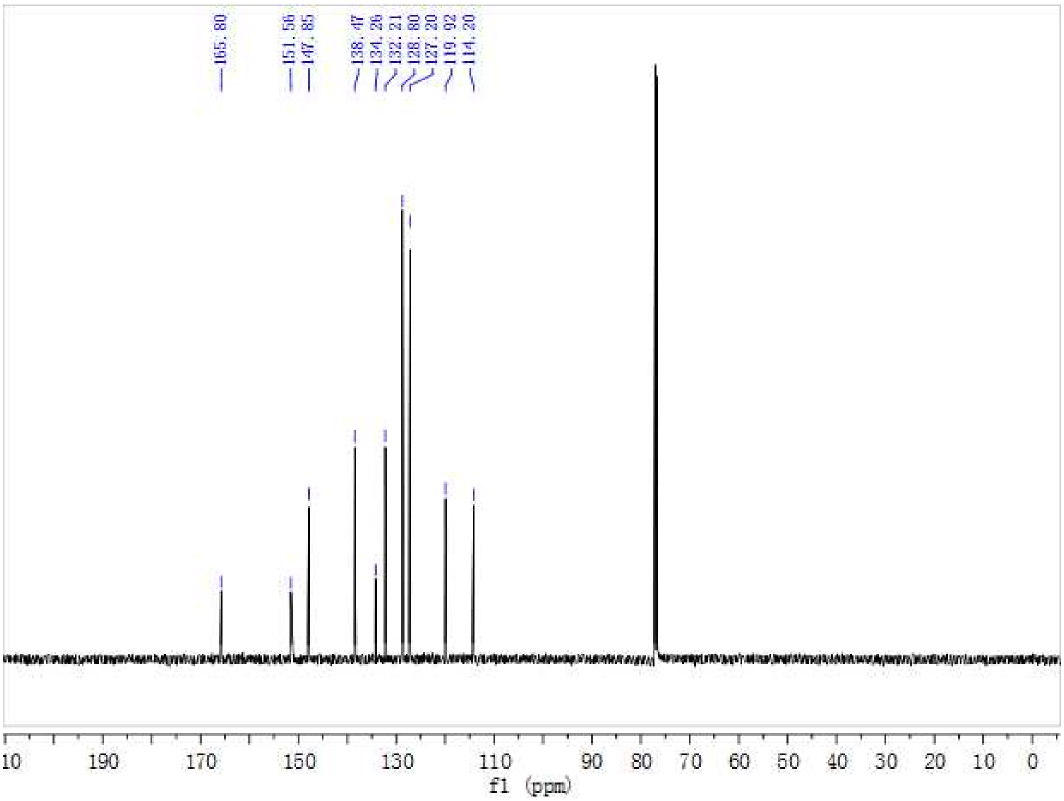

N-(Pyridin-2-yl)-2-naphthamide (CAS : 159257-88-2)

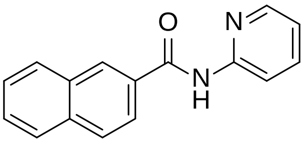

31

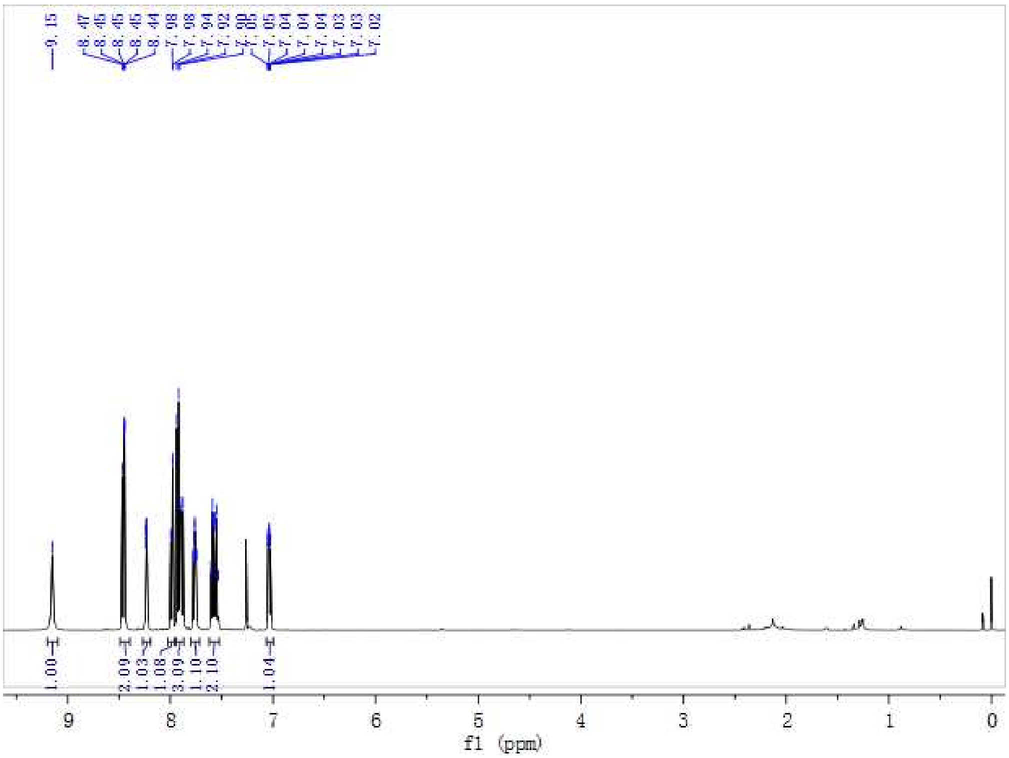

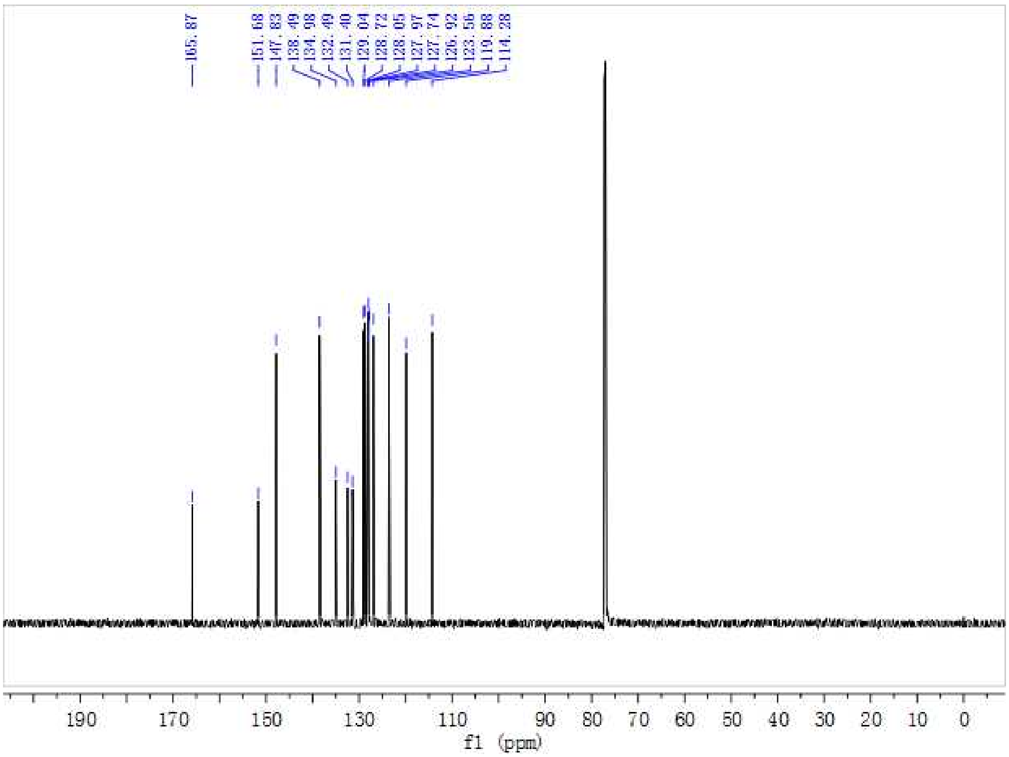

(1*H*-Indazol-1-yl)(phenyl)methanone (CAS: 23301-00-0)

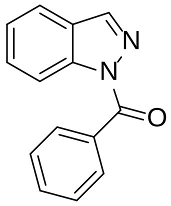

32

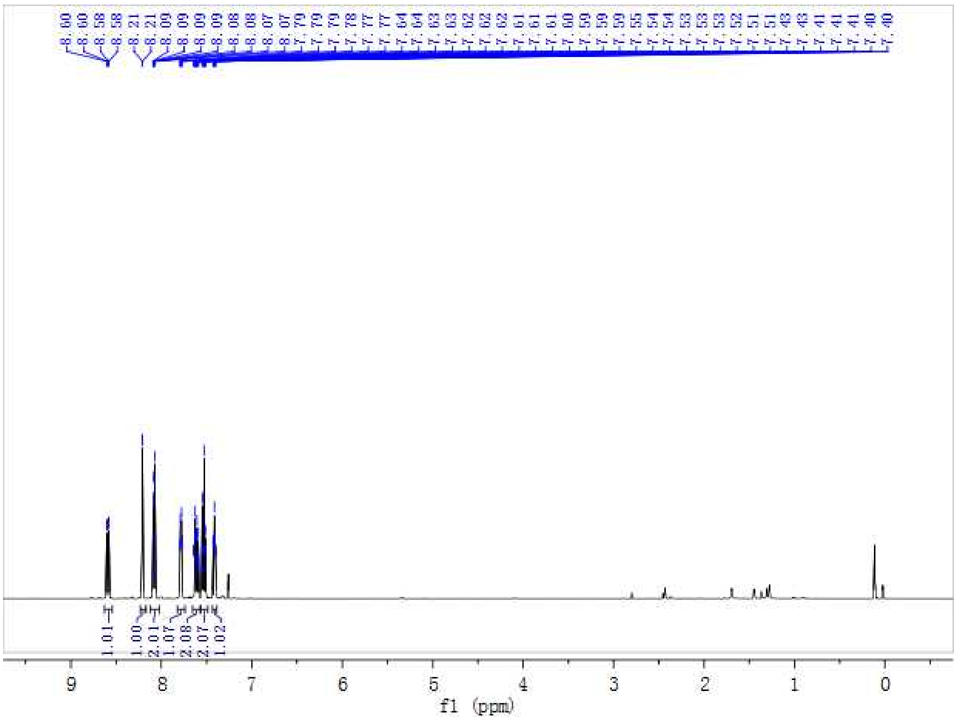

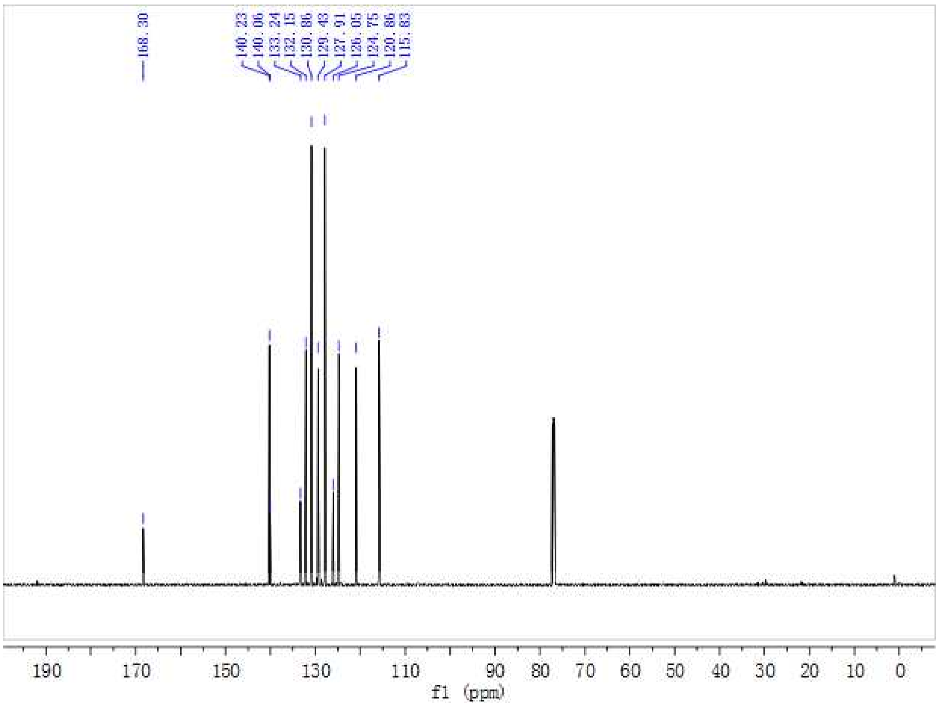

(1*H*-Benzo[*d*][1,2,3]triazol-1-yl)(phenyl)methanone (CAS: 4231-62-3)

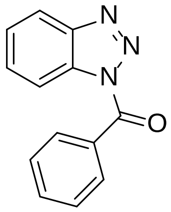

33

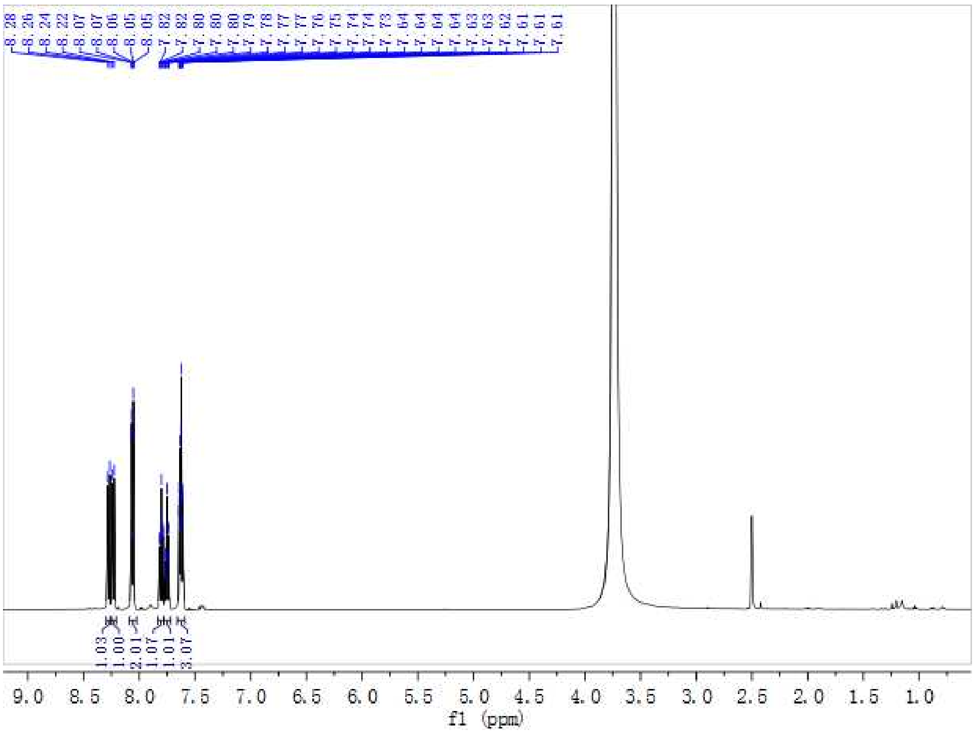

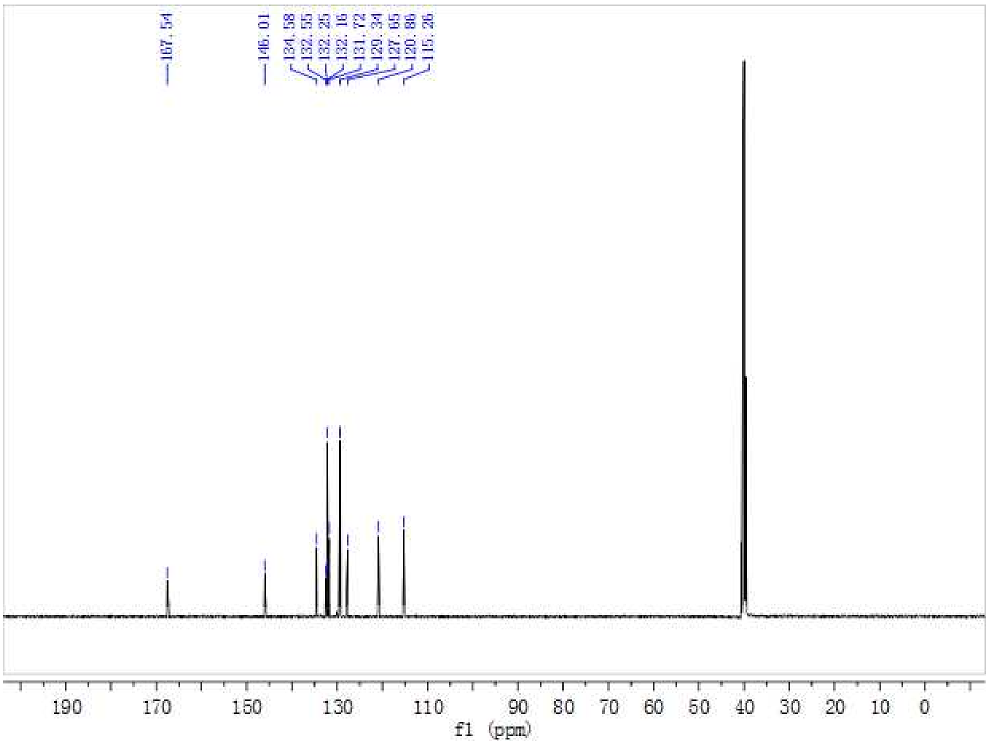

_N_-Phenylbenzamide (CAS: 93-98-1)

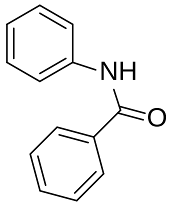

34

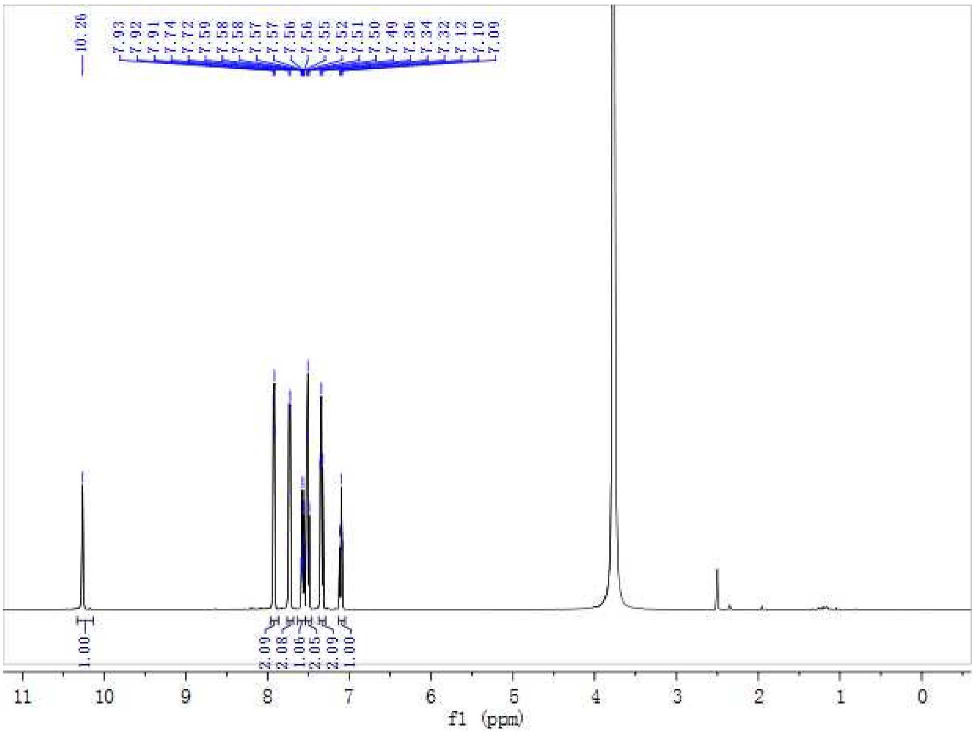

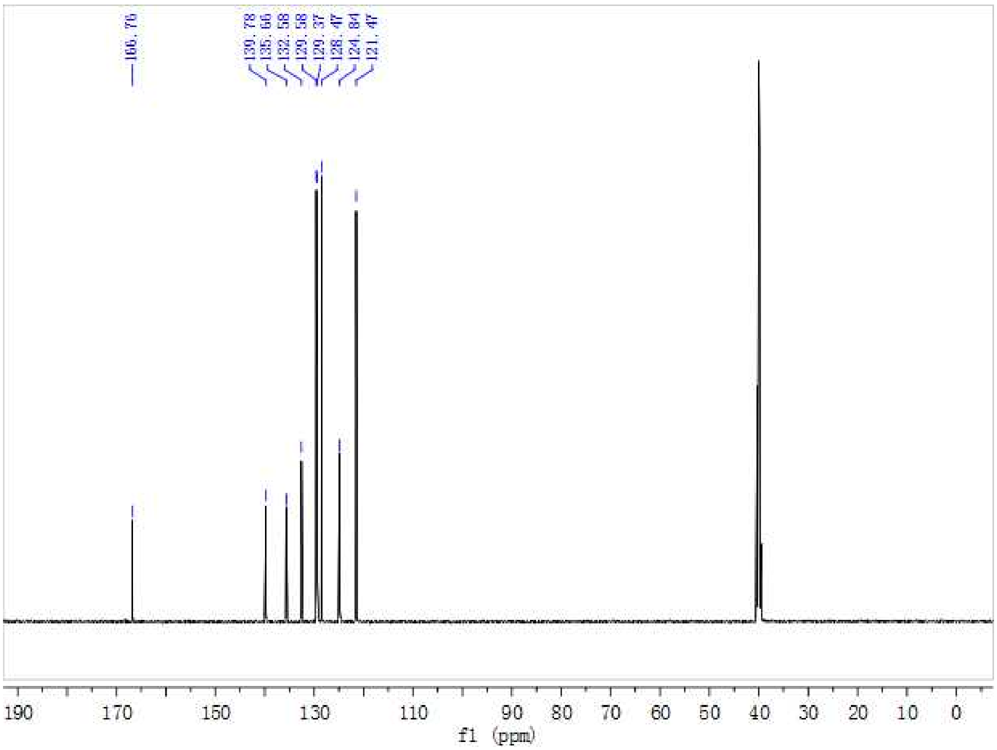

***N*-Benzyl-*N*-methylbenzamide (CAS: 61802-83-3)**

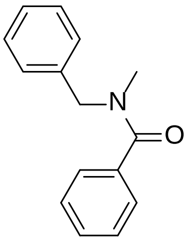

35

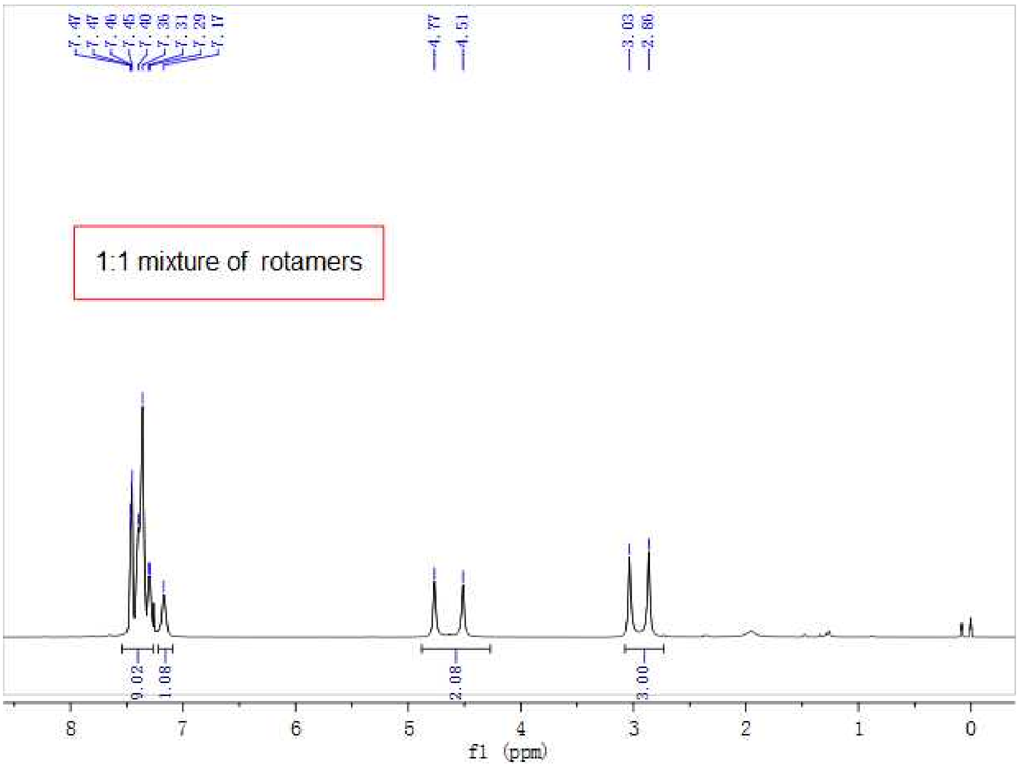

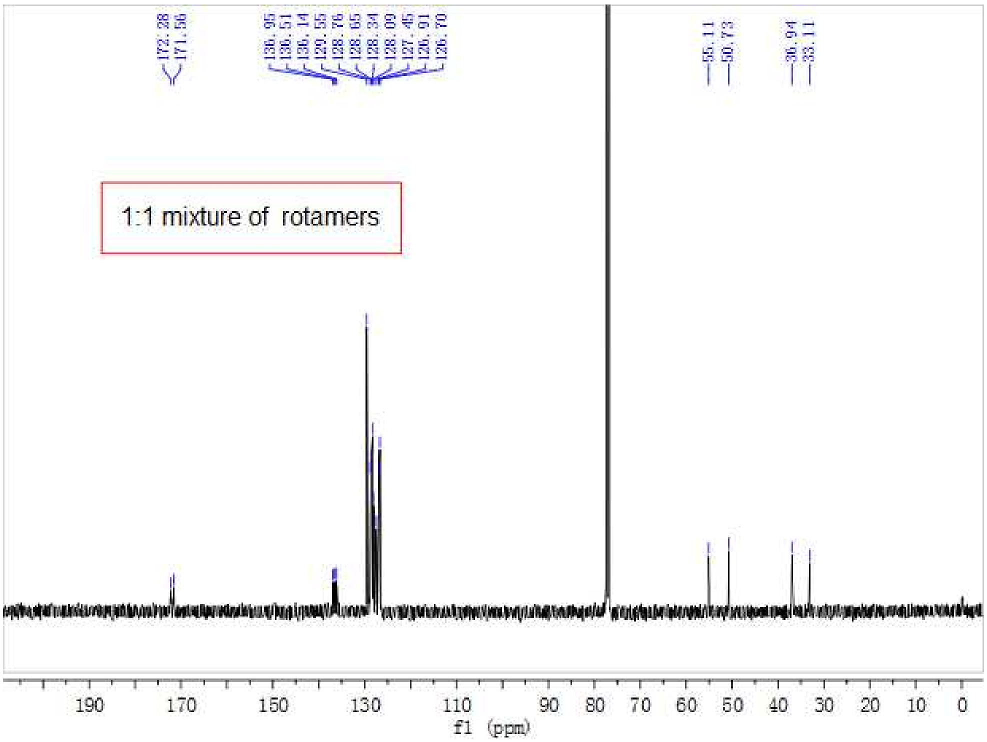

Phenyl(3-phenyl-1*H*-pyrazol-1-yl)methanone (CAS: 126382-89-6)

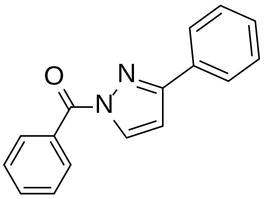

36

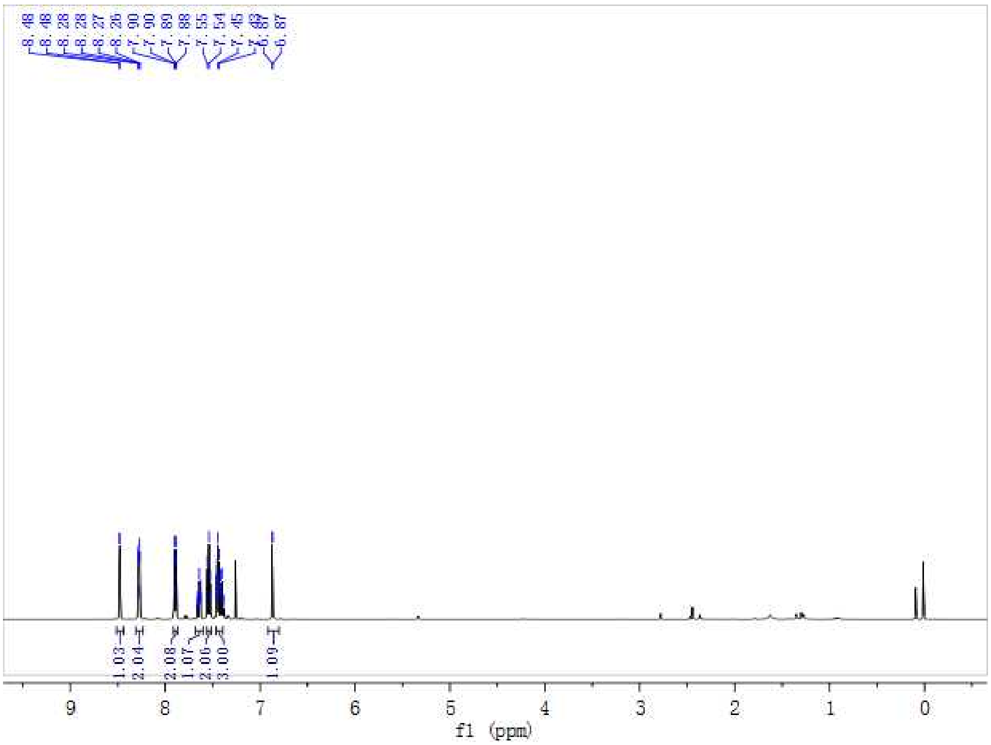

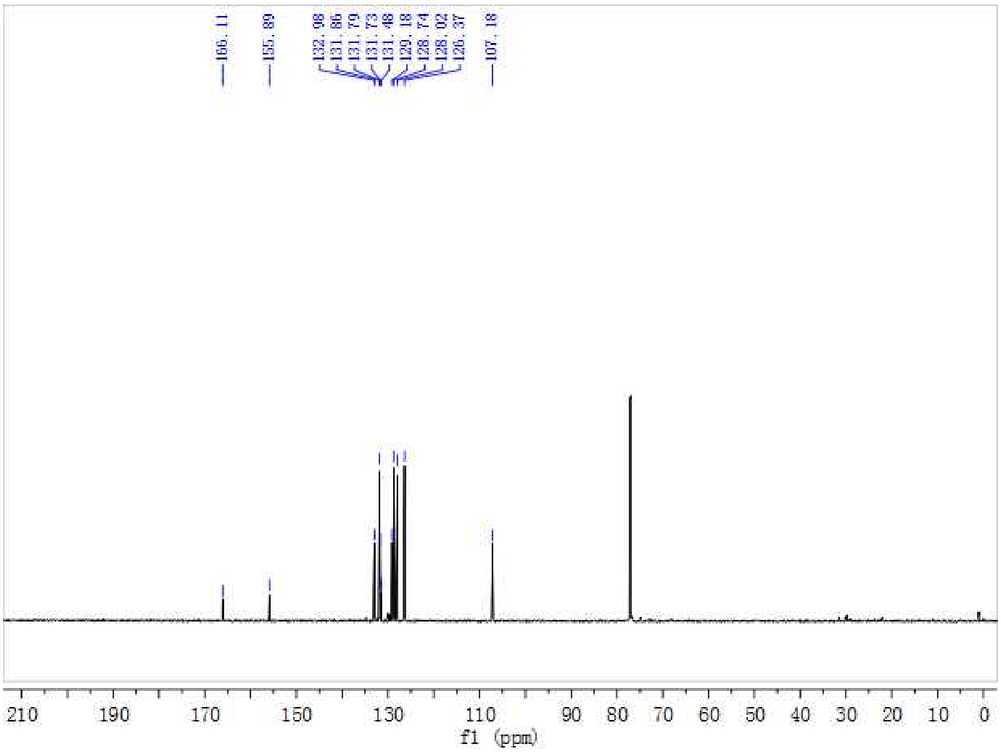

***N*-(Pyridin-2-yl)cyclohexanecarboxamide (CAS: 68134-77-0)**

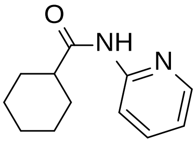

37

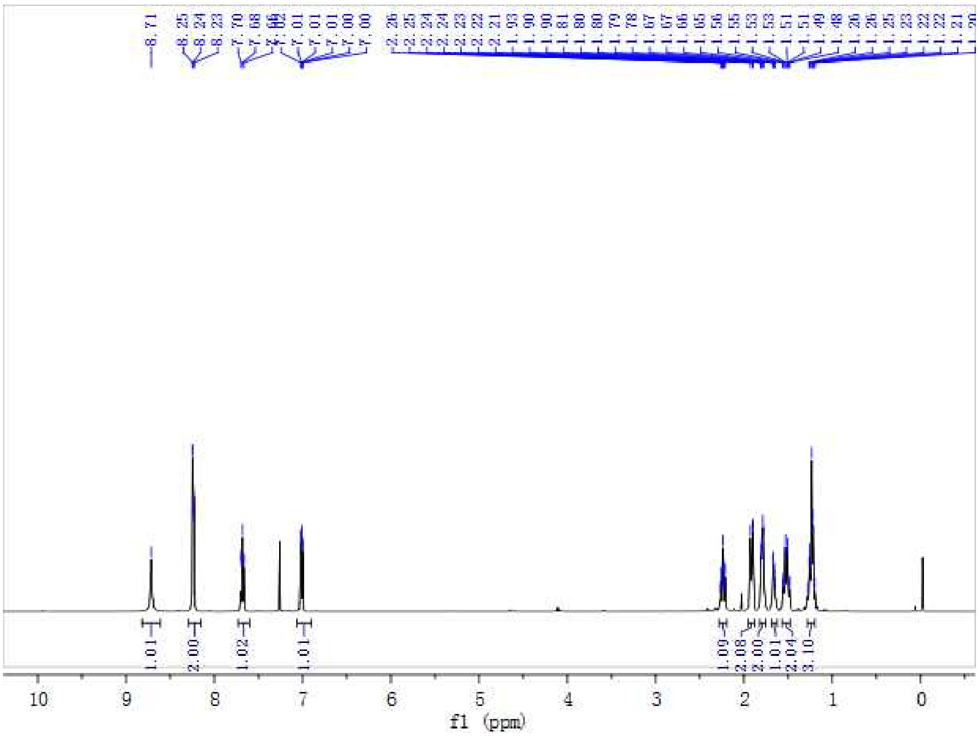

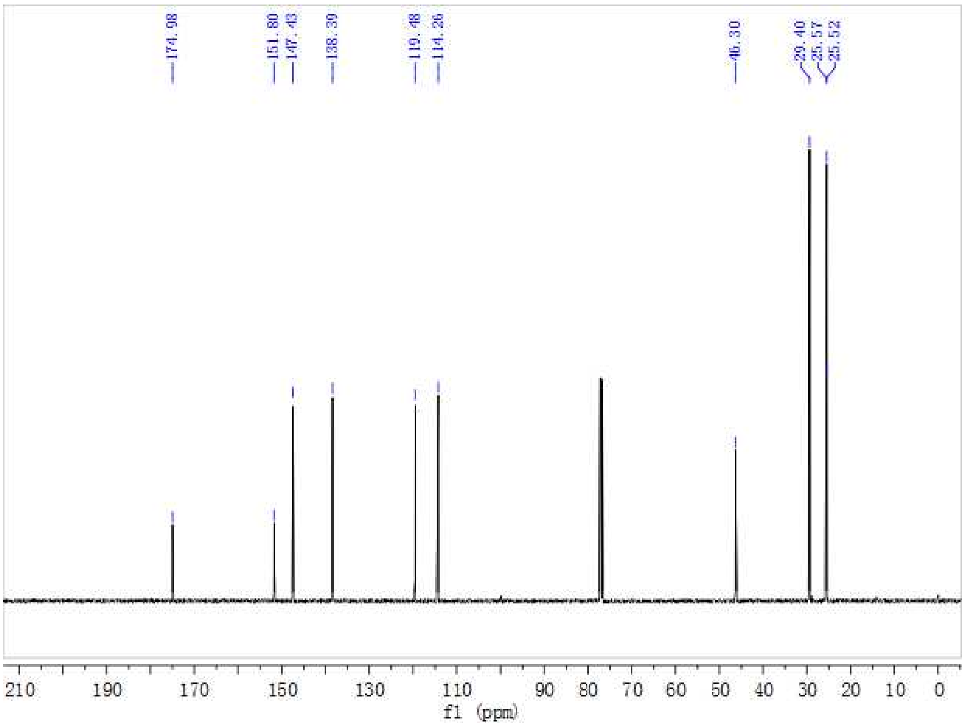

Phenyl(piperidin-1-yl)methanone (CAS: 776-75-0)

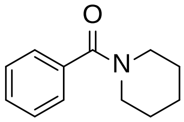

38

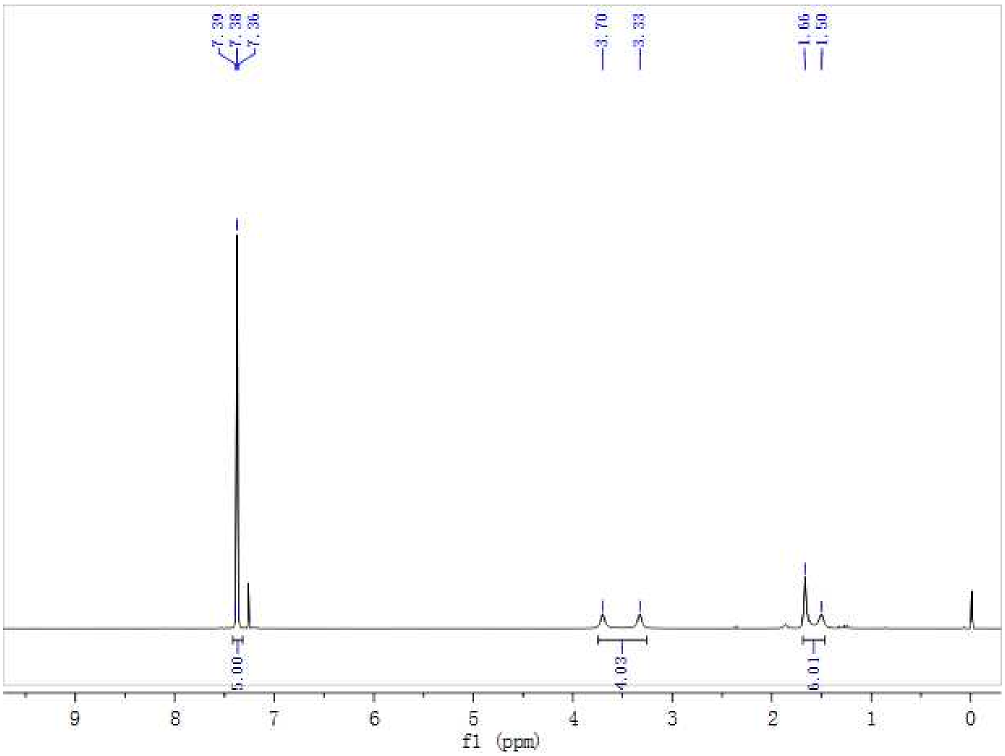

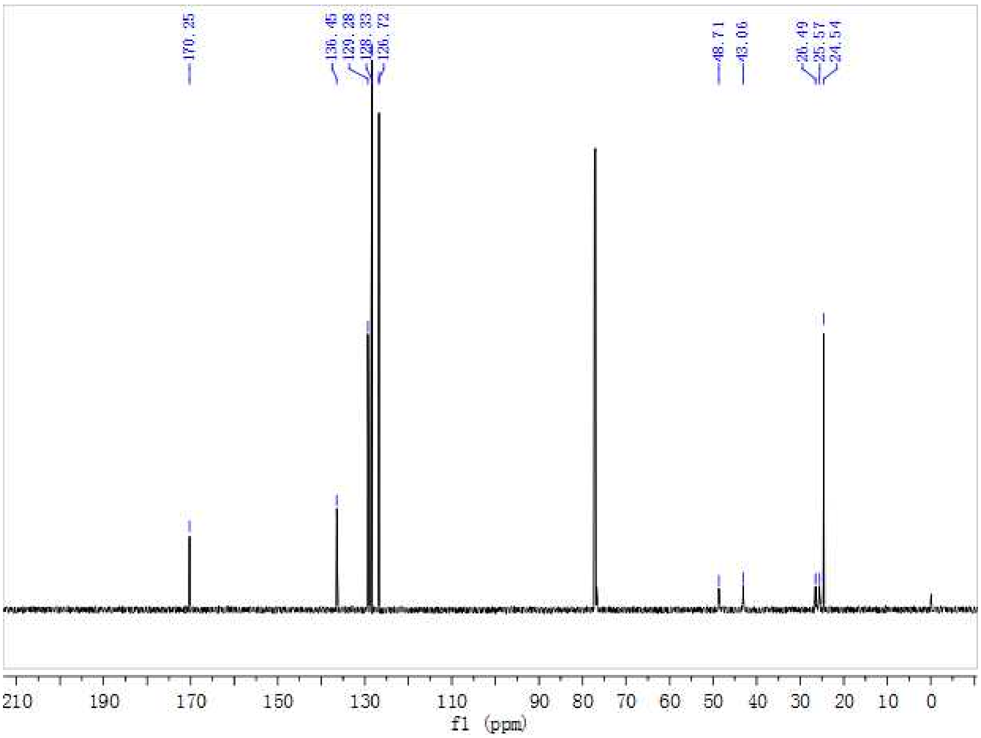

***N*,*N*-Diethylbenzamide (CAS: 1696-17-9)**

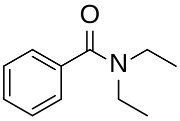

39

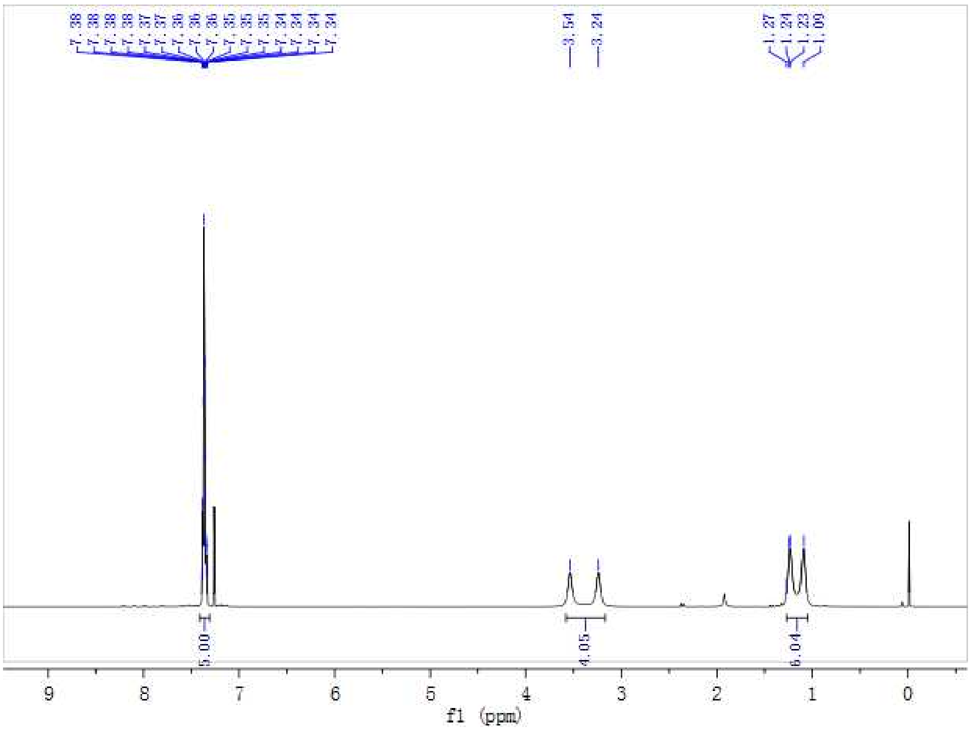

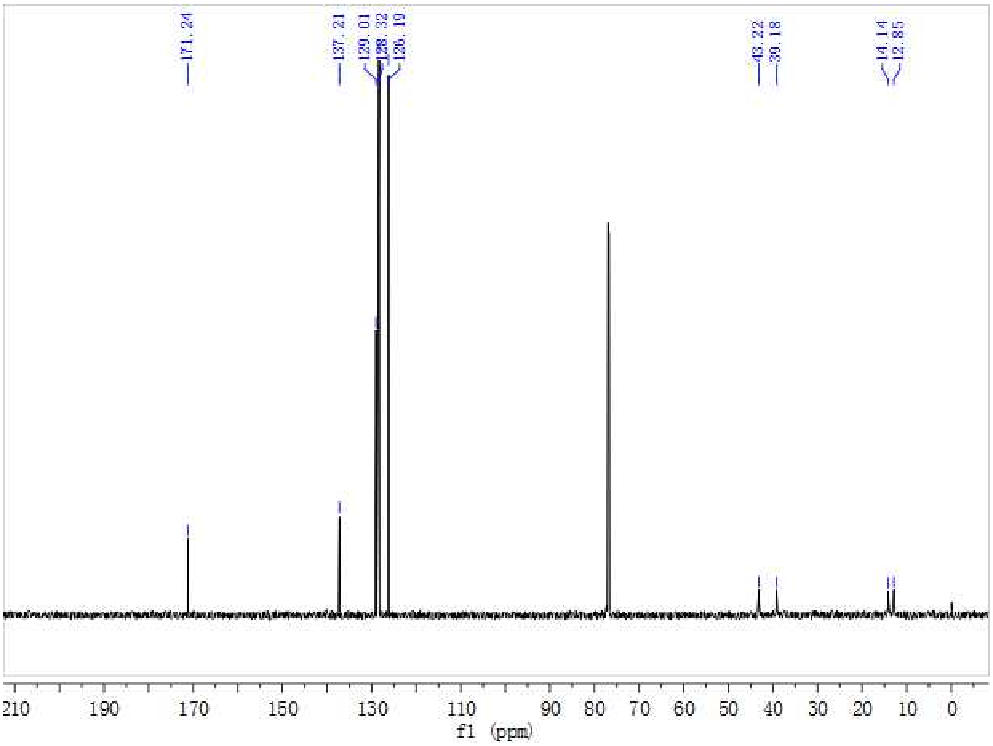

Methyl benzoyl-L-leucinate (CAS: 3005-60-5)

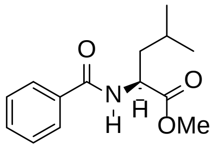

40

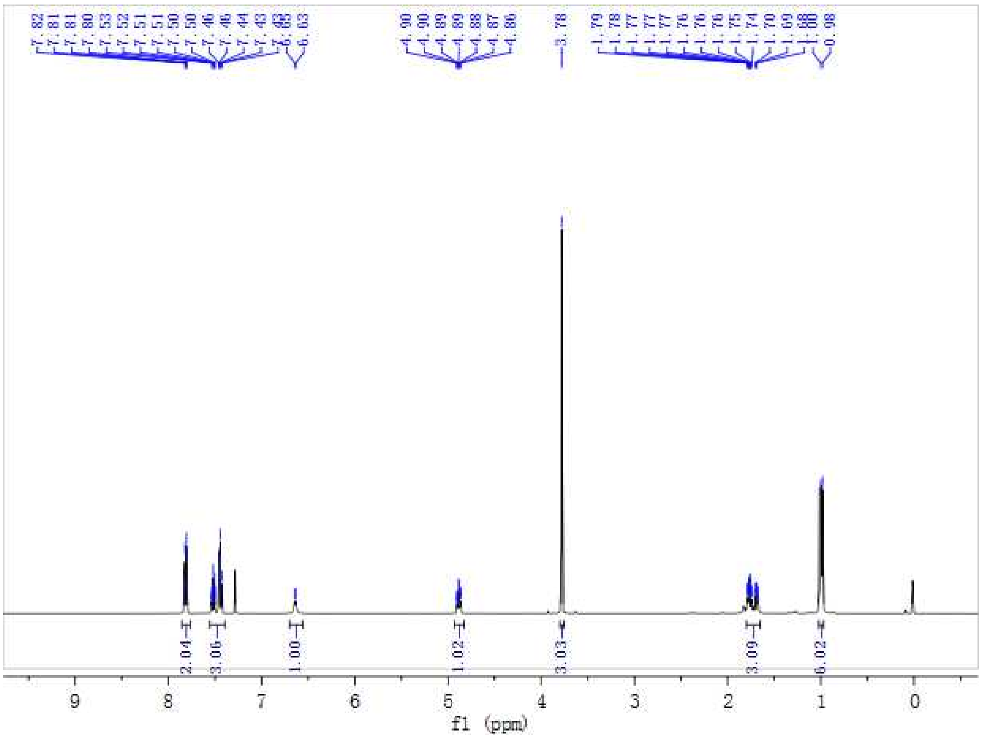

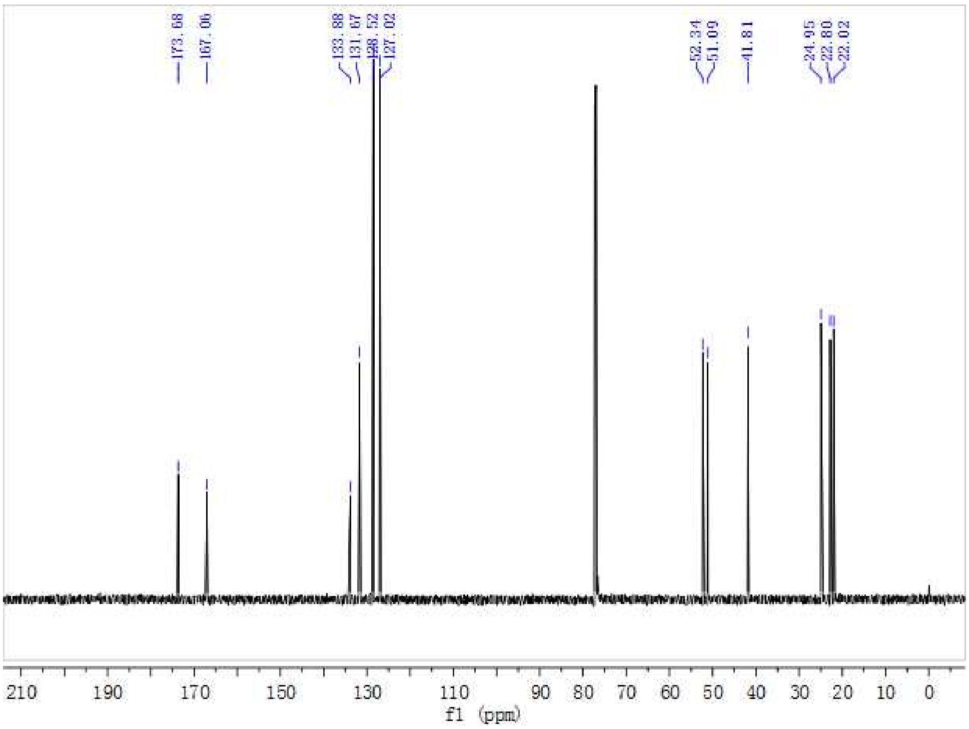

Supplement: File 1 — Experimental procedures, analytical data and NMR spectra. [file Beilstein_J_Org_Chem-11-2158-s001.pdf]
